# Impact on child acute malnutrition of integrating a preventive nutrition package into facility-based screening for acute malnutrition during well-baby consultation: A cluster-randomized controlled trial in Burkina Faso

**DOI:** 10.1371/journal.pmed.1002877

**Published:** 2019-08-27

**Authors:** Elodie Becquey, Lieven Huybregts, Amanda Zongrone, Agnes Le Port, Jef L. Leroy, Rahul Rawat, Mariama Touré, Marie T. Ruel

**Affiliations:** Poverty, Health and Nutrition Division, International Food Policy Research Institute, Washington, DC, United States of America; London School of Hygiene and Tropical Medicine, UNITED KINGDOM

## Abstract

**Background:**

Community management of acute malnutrition (CMAM) is a highly efficacious approach for treating acute malnutrition (AM) in children who would otherwise be at significantly increased risk of mortality. In program settings, however, CMAM’s effectiveness is limited because of low screening coverage of AM, in part because of the lack of perceived benefits for caregivers. In Burkina Faso, monthly screening for AM of children <2 years of age is conducted during well-baby consultations (consultation du nourrisson sain [CNS]) at health centers. We hypothesized that the integration of a preventive package including age-appropriate behavior change communication (BCC) on nutrition, health, and hygiene practices and a monthly supply of small-quantity lipid-based nutrient supplements (SQ-LNSs) to the monthly screening would increase AM screening and treatment coverage and decrease the incidence and prevalence of AM.

**Methods and findings:**

We used a cluster-randomized controlled trial and allocated 16 health centers to the intervention group and 16 to a comparison group. Both groups had access to standard CMAM and CNS services; caregivers in the intervention group also received age-appropriate monthly BCC and SQ-LNS for children >6 months of age. We used two study designs: (1) a repeated cross-sectional study of children 0–17 months old (*n* = 2,318 and 2,317 at baseline and endline 2 years later) to assess impacts on AM screening coverage, treatment coverage, and prevalence; (2) a longitudinal study of 2,113 children enrolled soon after birth and followed up monthly for 18 months to assess impacts on AM screening coverage, treatment coverage, and incidence. Data were analyzed as intent to treat. Level of significance for primary outcomes was α = 0.016 after adjustment for multiple testing. Children’s average age was 8.8 ± 4.9 months in the intervention group and 8.9 ± 5.0 months in the comparison group at baseline and, respectively, 0.66 ± 0.32 and 0.67 ± 0.33 months at enrollment in the longitudinal study. Relative to the comparison group, the intervention group had significantly higher monthly AM screening coverage (cross-sectional study: +18 percentage points [pp], 95% CI 10–26, *P* < 0.001; longitudinal study: +23 pp, 95% CI 17–29, *P* < 0.001). There were no impacts on either AM treatment coverage (cross-sectional study: +8.0 pp, 95% CI 0.09–16, *P* = 0.047; longitudinal study: +7.7 pp, 95% CI −1.2 to 17, *P* = 0.090), AM incidence (longitudinal study: incidence rate ratio = 0.98, 95% CI 0.75–1.3, *P* = 0.88), or AM prevalence (cross-sectional study: −0.46 pp, 95% CI −4.4 to 3.5, *P* = 0.82). A study limitation is the referral of AM cases (for ethical reasons) by study enumerators as part of the monthly measurement in the longitudinal study that may have attenuated the detectable impact on AM treatment coverage.

**Conclusions:**

Adding a preventive package to CMAM delivered at health facilities in Burkina Faso increased participation in monthly AM screening, thus overcoming a major impediment to CMAM effectiveness. The lack of impact on AM treatment coverage and on AM prevalence and incidence calls for research to address the remaining barriers to uptake of preventive and treatment services at the health center and to identify and test complementary approaches to bring integrated preventive and CMAM services closer to the community while ensuring high-quality implementation and service delivery.

**Trial registration:**

ClinicalTrials.gov NCT02245152.

## Abstract 2

Elodie Becquey and colleagues show increased participation in acute malnutrition screening when included in well-baby clinics in Burkina Faso.

## Author summary

**Why was this study done?:**

- Fifty-two million children <5 years old worldwide are affected by acute malnutrition (AM).
- Community management of AM (CMAM), which consists of regular screening in the community to detect AM in young children and of treatment of AM cases at home, could save 435,000 lives each year if 90% of AM children were covered.
- In most locations with a high burden of AM, CMAM coverage is below 50%, mainly because of the lack of community awareness and recognition of child AM and the lack of participation in regular screening and treatment seeking due to lack of knowledge of existing CMAM programs, previous rejection from a CMAM program, and/or high travel and opportunity costs for caregivers of those participating in CMAM treatment.

**What did the researchers do and find?:**

- We tested the impact of incorporating preventive nutrition interventions into the AM screening conducted at health centers in Burkina Faso to assess whether it increased attendance at screening (and therefore the percentage of children screened and treated) and reduced the prevalence and incidence of AM.
- The preventive intervention included nutrition, health, and hygiene education for caregivers of children 0–23 months of age and the provision of a lipid-based nutrition supplement containing energy and essential micronutrients for children 6–23 months of age.
- Sixteen health centers were randomly allocated to the intervention arm and were compared to 16 health centers that offered the usual screening services.
- In each arm, we surveyed a sample of approximately 1,152 children 0–17 months of age before the start of the intervention (at baseline) and another sample of the same age and size 2 years later (at endline). We also enrolled a sample of roughly 1,056 children at birth in each arm and followed them monthly until they reached 18 months of age.
- The proportion of children screened for AM was 18–23 percentage points higher in the intervention compared to the comparison arm. However, we found no impact on the proportion of children who received appropriate treatment for AM or in the prevalence or incidence of AM.

**What do these findings mean?:**

- Preventive services, especially the nutritional supplement, were a powerful incentive for caregivers to bring their children to screening sessions.
- There are, however, many more barriers that need to be addressed to achieve full coverage of preventive services and full child AM screening coverage in Burkina Faso and to ensure that once children have been identified as suffering from AM, they are effectively referred to the CMAM program, receive full treatment, and recover.
- More research is needed to identify solutions to boost utilization of health center–based CMAM services where they are operational and to test complementary approaches to bring integrated preventive and AM treatment services closer to the community while ensuring good quality.

## Introduction

Wasting, an indicator of acute malnutrition (AM), affects 52 million children globally [1] and is a major cause of death in children under 5 years of age. Scaling up the effective management of AM to 90% coverage would save an estimated 435,000 lives each year [2]. The community management of AM (CMAM) is the World Health Organization’s recommended approach for AM treatment [3,4] and is offered at scale in many low-income countries. It consists of the active screening for AM at the community level and the ambulatory treatment of uncomplicated cases of severe AM (SAM) and moderate AM (MAM) with ready-to-use therapeutic and supplementary foods, respectively. SAM cases with medical complications or without appetite are referred to inpatient treatment. Typically, active screening for AM is done by community health workers or volunteers in the communities. They refer identified cases to the health system to confirm the diagnosis and to enroll them in the CMAM program.

Recent estimates reveal a generally low coverage of CMAM (below 50% in most locations) [5]. Main barriers to treatment include the lack of community awareness and recognition of child AM, the lack of caregiver knowledge of existing CMAM programs, the failure of caregivers to seek treatment at the health center following a previous rejection from a CMAM program (e.g., because the child was not acutely malnourished), and the travel and opportunity costs for caregivers of participating in CMAM treatment [6]. Innovative solutions are urgently needed to incentivize caregivers to seek and maintain contact with CMAM programs, for screening and for treatment when required.

This manuscript reports on a study conducted in Burkina Faso study as part of the Innovative Approaches for the Prevention of Childhood Undernutrition (PROMIS) project carried out in Burkina Faso and Mali. In both countries, the PROMIS project integrated preventive interventions into AM screening to incentivize mothers to participate in screening and to increase CMAM treatment coverage and reduce AM incidence and prevalence [7]. The innovative aspect of the intervention was the combination of approaches to prevent undernutrition (behavior change communication [BCC] on nutrition, health, and hygiene and the provision of small-quantity lipid-based nutrient supplements [SQ-LNSs]) with AM screening and referral. The project thus established a continuum of care—from prevention to treatment—through a single platform. We hypothesized that integrating prevention into the screening for and referral of AM cases would reduce child AM prevalence through reductions in incidence (a consequence of the nutritional benefits of the preventive intervention) and reductions in episode length (due to better AM case detection and referral).

This paper presents findings from the study in Burkina Faso, which used a health facility platform to integrate the preventive intervention package into the CMAM program. Results from the study in Mali, where the preventive intervention was integrated into a community-based screening platform, are presented in the companion paper [8].

## Methods

The study protocol was published previously [7]. A summary of the methods is provided here.

### Study context

The study was carried out in the Gourcy health district of the Zondoma Province in Burkina Faso’s Northern Region. This area has a high burden of child AM. At the time the study was designed in 2013, the prevalence of AM in children 0–59 months old in Zondoma was 13.0% (versus 8.2% at the national level), and the prevalence of SAM was 3.0% (versus 1.7% nationally) [9]. The area includes 114 villages and about 42,500 inhabitants below 5 years of age [10].

### CMAM program in Burkina Faso

Burkina Faso launched its first national protocol for the management of AM in 2007 and included CMAM in its 2014 protocol [11]. CMAM is part of the national health strategy and is coordinated at the national level by the ministry of health’s Directorate for Nutrition. It covers children 0–59 months old and uses both community and health facility platforms. At the community level, CMAM is implemented by community health workers and volunteers. Activities include raising malnutrition and CMAM awareness and active AM screening through exhaustive door-to-door screening campaigns up to four times per year. Community health workers and volunteers are also expected to carry out passive screening in the community when they are in contact with young children for any purpose. Children with a mid-upper arm circumference (MUAC) below 125 mm or bilateral pitting edema (criteria used at community level to detect AM cases) are referred to the health center for future screening and enrollment in CMAM.

At health facilities, passive AM screening is expected to be done during every contact between health workers and children under 5 years of age, including during sick child consultations, hospitalization, postnatal consultations, vaccination, or monthly well-baby consultations (“consultation du nourrisson sain” [CNS] in French) for children aged 1–23 months. The CNS typically includes the following services: growth monitoring; group counseling on hygiene, health, and/or nutrition practices; vitamin A supplementation; vaccination; and overall health status monitoring. Given that the CNS offers the most comprehensive package of preventive services for children aged 1–23 months, and the fact that the implementing partner had made a commitment to boost health center attendance, we selected the CNS at health facilities as the target platform for the PROMIS intervention.

During passive screening at health centers and when confirming the diagnosis of a child referred for AM, health facility staff assesses MUAC, weight for length, and the presence of bilateral pitting edema. MAM is defined as having a weight-for-length z-score (WLZ) between −2 and −3 (all ages) or an MUAC between 115 mm and 125 mm (children above 6 months of age); SAM is defined as having a WLZ below −3 (all ages), an MUAC below 115 mm (children above 6 months of age), or bilateral pitting edema (all ages) [11]. Children suffering from SAM with edema, medical complications, or loss of appetite or who are younger than 6 months of age are enrolled for inpatient treatment, most often at the main district hospital. All other children are enrolled in the CMAM program and given a CMAM beneficiary card. Children with MAM receive a 14-day supply of ready-to-use supplementary food at a consultation at the health center every 2 weeks. Children with SAM receive a weekly supply of ready-to-use therapeutic food at weekly consultations. Community-level CMAM activities include case follow-up to improve treatment adherence. The food is supplied until complete recovery, which is defined for both conditions as a WLZ ≥ −2 (all ages) and an MUAC ≥ 125 mm (children above 6 months of age) at two consecutive visits.

### Intervention and theory of change

The PROMIS project was implemented by Helen Keller International (HKI) from April 2015 to June 2017 and targeted children 0–59 months of age. The project’s theory of change is shown in Box 1. Some PROMIS activities were implemented in both the intervention and comparison groups; others were limited to the intervention group.

**Box 1. Theory of change of the PROMIS project**

**Fig 1:**
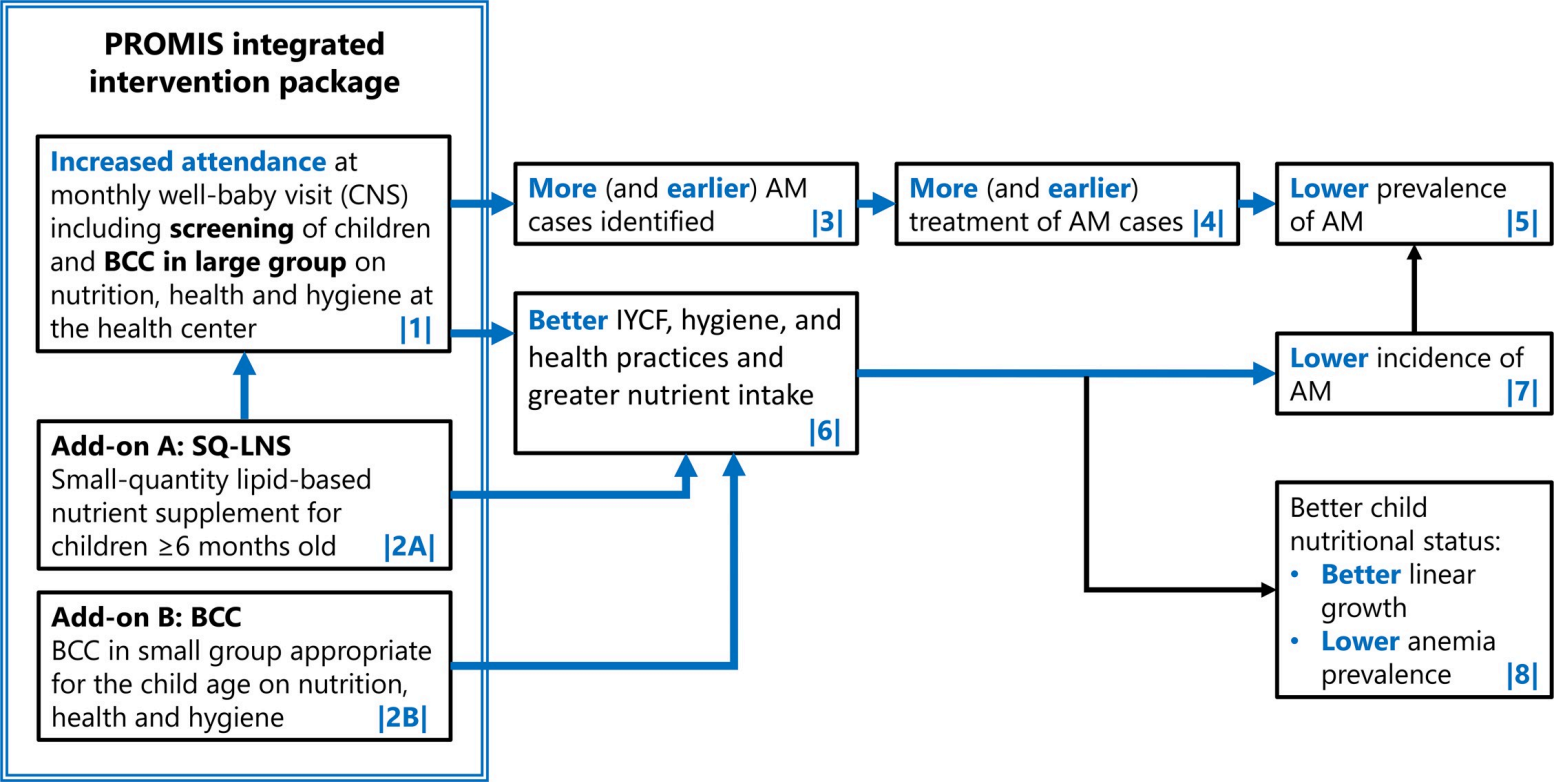
Theory of change of the integration of a preventive package at the well-baby visit. Hypothesized impacts of the intervention presented in this paper are shown in blue. AM, acute malnutrition; BCC, behavior change communication; IYCF, infant and young child feeding; PROMIS, Innovative Approaches for the Prevention of Childhood Undernutrition; SN-LNS, small-quantity lipid-based nutrition supplement.

The preventive nutrition package integrated into AM screening was hypothesized to lead to increased AM screening and a reduction in AM prevalence and incidence through two main pathways: (1) greater likelihood of (early) detection and treatment (referred to as the “treatment pathway”) and (2) better AM prevention (referred to as the “prevention” pathway). Along the treatment pathway, it was expected that more caregivers would bring their children to the CNS (box 1 in Fig 1) because of the incentive value of the preventive package (2A and 2B) and the requirement of AM screening in order to receive SQ-LNS (2A). This, in turn, was anticipated to lead to a higher proportion of AM cases identified earlier (3), a higher proportion of AM children being referred and enrolled for treatment (4), a higher AM recovery rate, and, as a result, a lower prevalence of MAM and SAM (5). Along the prevention pathway, SQ-LNS (2A) was expected to improve the energy and micronutrient adequacy of the diet of eligible children. Together with age-appropriate, small-group BCC (2B), it was expected to improve nutrition, hygiene, and health practices (6). Better practices were expected to lead to lower AM incidence (7), resulting in an overall lower AM prevalence (5). Besides the impact on AM, better practices were also expected to lead to higher linear growth and lower anemia prevalence (8); results from these outcomes are not presented here.

#### PROMIS activities in the intervention and comparison groups

The PROMIS project strengthened implementation of the national CMAM protocol described above through technical support by HKI with training, formative supervision, and advocacy targeted to health staff and authorities across all health centers and all villages in the Gourcy health district. Specifically, this included the following activities: technical support for quality implementation of consultations for young children at the health center (CNS, sick child consultation, and SAM and MAM consultations), financial and technical support for quarterly community door-to-door AM screening campaigns, and the mobilization of key community members to strengthen CMAM at community level. At both the health center level and the community level, training and supportive supervision were provided for CMAM, AM screening, and BCC (including negotiation techniques for behavior change).

#### Additional PROMIS activities in the intervention group

In addition to the activities described above, a preventive package targeted to children under 2 years of age and their caregivers was integrated into the existing monthly CNS AM screening and referral and implemented by the existing health system in randomly selected health centers (see Study design section below). Caregivers who visited CNS in these health centers received (1) monthly age-appropriate BCC on nutrition, health, and/or hygiene practices [12,13] organized in groups of maximum seven caregivers and (2) a monthly supply of 20-g sachets of SQ-LNS (Nutributter, Nutriset, Malaunay, France) intended for daily use. This preventive supplement was provided to children above 6 months of age not suffering from AM and not under AM treatment. Age-appropriate BCC topics included exclusive breastfeeding, infant and young child feeding (IYCF), use of nutrition supplements and fortified foods, use of preventive health services, child feeding during illness, hygiene and sanitation, diet and nutrition during pregnancy and lactation, and appropriate use of SQ-LNS (one sachet per day in addition to complementary feeding for non-AM children 6–24 months old). Further details on the BCC approach and SQ-LNS composition are provided elsewhere [7]. Receiving the preventive package was conditional upon participating in the monthly AM screening at CNS.

### Study design

A two-arm, cluster-randomized, nonblinded, effectiveness trial was used to assess the impact of the integrated preventive package (i.e., age-appropriate BCC in small groups on nutrition, health, and hygiene practices and SQ-LNS added to the existing monthly CNS AM screening and group BCC) as compared to the standard of care (i.e., monthly CNS AM screening and group BCC). A cluster design was used because individual randomization of the health facility–based intervention was not feasible.

The health center catchment area was used as the unit of randomization. Allocation to intervention or comparison group was performed through a public lottery. All rural health centers in the Gourcy district (*n* = 32) were eligible. Sixteen health centers were randomly assigned to the intervention group and an equal number to the comparison group. All children below 2 years of age living in the catchment area of the intervention health centers received a PROMIS beneficiary card.

To assess the impact of the intervention, we used a dual evaluation design, including a repeated cross-sectional study and a longitudinal study. The cross-sectional study was used to assess the overall impact of the intervention on AM screening coverage, treatment coverage, and prevalence after 2 years of exposure to the program. Baseline and endline surveys were conducted at the same time of year to minimize the effect of seasonality. The longitudinal study followed a closed cohort of children from soon after birth to 18 months of age and assessed the impact of the PROMIS project on AM screening coverage, treatment coverage, and AM incidence as well as secondary outcomes (described below).

### Sampling design and sample size calculations

For the cross-sectional study, assuming a coefficient of intercluster (i.e., between health center catchment areas) variation k of 0.25, a nonresponse rate of 15%, a type I error of 5%, and a statistical power of 80%, we calculated that with an average cluster (i.e., health center catchment area) size of 72 children, 32 clusters (i.e., an overall sample size of 2,304 children) were needed for each survey round to detect a decrease in the prevalence of AM of 5.4 percentage points (pp) assuming a baseline prevalence of 16%. This sample size allowed to detect a difference in AM screening coverage of 7.5 pp and a difference in AM treatment coverage of 20 pp between study arms assuming baseline values of 25% for both outcomes.

For the longitudinal study, assuming a coefficient of intercluster variation k of 0.2, a dropout rate of 20%, a type I error of 5%, and a statistical power of 80%, we needed to recruit 66 children in each of the 32 clusters (i.e., an overall sample size of 2,112 children total) to detect a 23.5% reduction in the incidence of AM over 18 months of follow-up, assuming a baseline incidence of 0.52 cases per child-year. This sample size allowed detection of a difference in AM screening coverage of 5.9 pp and a difference in treatment coverage of 9.0 pp during the 18 months of follow-up assuming an incidence of 0.52 cases per child-year and baseline values of 25% for both outcomes.

Detailed sampling design is described elsewhere [7]. In each health center catchment area, a census was conducted 1 month prior to the cross-sectional baseline and endline surveys to identify all pregnant women and eligible children. A random sample of children was drawn from the census list. The endline survey occurred 2 years after baseline in the same communities.

Eligibility criteria for the cross-sectional and the longitudinal study are presented in Table 1. Recruitment of newborn children in the longitudinal study happened after completion of the cross-sectional baseline survey, so no children were included in both surveys. The cross-sectional endline survey was conducted when the longitudinal study children were 14 to 21 months old. Therefore, some of the children in the endline cross-sectional survey (i.e., those 14 to 17 months old at the time) may also be included in the longitudinal study.

**Table 1:** Inclusion criteria and primary and secondary study outcomes for the cross-sectional and longitudinal studies.

	Cross-sectional study	Longitudinal study
Inclusion criteria		At study enrollment:
	(1) being a singleton child 0–17.9 months (±1 week) of age	(1) being a singleton child 0–6 weeks of age
	(2) not having congenital malformations that hinder growth and/or anthropometric measurements	(2) not having congenital malformations that hinder growth and/or anthropometric measurements
	(3) child’s principal caregiver having lived in the study area since the child was born	(3) child’s principal caregiver planning to reside in the village for the next year
	** **	(4) child not suffering from AM at enrollment, defined as WLZ < −2 at enrollment and the first follow-up (to avoid normal postnatal weight loss to result in exclusion for AM)
Primary study outcomes	(1) AM screening coverage (the number of children screened for AM in the past month over the total number of study children)	(1) AM screening coverage (the number of children screened for AM in the past month over the total number of study children considering all monthly visits over the 18-month follow-up)^a^
	(2) AM treatment coverage (number of children with AM under appropriate treatment for their condition [SAM or MAM] in the past month over the total number of AM cases identified at the time of the survey in the study sample)	(2) AM treatment coverage in children enrolled in the CMAM program (the number of AM episodes for which MAM or SAM treatment was received until discharged or recovery over the total number of AM episodes enrolled in a CMAM program over the 18-month follow-up)
	(3) AM prevalence (the number of cases of AM at survey time over the total number of study children)	(3) incidence of the first AM episode over the 18-month follow-up^b^
Secondary study outcomes	Program participation and coverage in the month preceding the survey:	Program participation and coverage over 18 months of follow-up:
	CNS coverage in the month preceding the survey;	CNS coverage
		change in CNS coverage over time
	AM screening coverage through CNS ^c^	AM screening coverage through CNS^c^
		change in AM screening coverage over time;
	BCC coverage (BCC delivered at CNS and through any channel)^c^	BCC coverage (BCC delivered at CNS and through any channel)^c^
		change in BCC coverage over time
	total SQ-LNS coverage	total SQ-LNS coverage
		change in SQ-LNS coverage over time
	AM:	AM:
	prevalence of MAM (−3 ≤ WLZ < −2 or 115 mm ≤ MUAC < 125 mm in children older than 6 months of age)	longitudinal prevalence of AM (defined as the total time the child was with AM over the total follow-up time)
	prevalence of SAM (WLZ < −3 or MUAC < 115 mm in children older than 6 months of age or presence of bilateral pitting edema)	longitudinal prevalence of MAM and SAM (total time the child was with MAM or SAM over the total follow-up time, respectively)
	AM status at the time of SQ-LNS distribution, as reported on the PROMIS beneficiary card or by the caregiver in the absence of PROMIS beneficiary card	change in AM prevalence over time
	mean WLZ	change in WLZ over time
	mean MUAC	change in MUAC over time
		Treatment enrollment and coverage:
		AM treatment enrollment and coverage (the number of MAM and SAM episodes in children enrolled in the CMAM program for which MAM- or SAM-appropriate treatment was received);
		MAM and SAM treatment enrollment and coverage (the number of MAM or SAM episodes in children enrolled in the CMAM program for which MAM- or SAM-appropriate treatment was received, respectively).
		Recovery, relapse, and episode length:
		recovery of AM, MAM, and SAM after treatment
		relapse rates of AM, MAM, and SAM
		mean AM, MAM, and SAM episode length

^a^The monthly measurements done by the research team included anthropometry. When children were identified by the research team as having AM, they were referred to the CMAM for ethical reasons. Our measure of screening coverage excludes these measurements, as they were not part of the program implementation activities

^b^We limited the analysis of the incidence to the first episode of AM to assess the impact of the preventive components of the intervention without possible interference of treatment of a previous episode. However, to assess the robustness of our findings, we also carried out the analysis using all episodes as a secondary outcome.

^c^Since AM screening and BCC were offered by multiple actors, we assessed the impact of the intervention on total AM screening and BCC coverage and specifically through the monthly CNS.

Abbreviations: AM, acute malnutrition; BCC, behavior change communication; CMAM, community management of AM; CNS, well-baby consultation; MAM, moderate AM; MUAC, mid-upper arm circumference; SAM, severe AM; SQ-LNS, small-quantity lipid-based nutrition supplement; WLZ, weight-for-length z-score

### Primary and secondary outcomes

The cross-sectional and longitudinal study both had three primary outcomes (Table 1): two outcomes related to AM screening and treatment and one related to AM, which was defined as WLZ < −2 (all ages), MUAC < 125 mm (children above 6 months of age), or the presence of bilateral pitting edema (all ages).

To complement the primary outcome findings, we also present results for all of the secondary outcomes that are directly related to AM (Table 1). The impact on the other secondary outcomes (IYCF knowledge and practices, linear growth, and anemia) will be published separately.

### Measurements and indicator creation

All household survey data were collected at home by trained enumerators. The caregiver of the index child was interviewed to collect data on the caregiver and the child. The primary respondent for questions related to the household was the household head. All interviews were conducted in the respondent’s language.

For all surveys, the child was considered to have been screened and the caregiver to have received BCC and/or SQ-LNS for the target child if either the caregiver recall or the systematic check of the PROMIS beneficiary card confirmed receipt of these respective services over the past month. Screening was defined as MUAC having been measured in a child ≥6 months old and/or weight and length measured (children of all ages).

Date of birth was collected from each child’s health booklet and, if not available, from a vaccination card or any other written record. When no written record was available (6.0% at baseline, 2.3% at endline), date of birth was estimated by the enumerator using a detailed calendar of local events.

All anthropometric measurements and checking of bilateral pitting edema were systematically performed by a trained and standardized anthropometrist with the help of a trained assistant. Standardization of weight, length, and MUAC measurements against the measurements of an anthropometry expert team was conducted prior to the start of fieldwork and every 2 months during the longitudinal study [14]. Child length and MUAC (in children above 6 months of age) were measured twice to the nearest millimeter, and child weight was measured twice to the nearest 100 g. A third measurement was taken if the difference between the two repeated measurements was >5 mm for length and MUAC and >300 g for weight. The average of the two (or three) repeated measurements was used for the calculation of nutritional status indicators. WLZ was calculated using the “zscore06” command in Stata [15], which uses the World Health Organization’s growth standard [16].

In the longitudinal study, an AM episode was defined as follows: it started when a child was found to be acutely malnourished at the monthly survey and ended when a child did not suffer from AM at one monthly measurement. MAM and SAM episodes were defined the same way, except that children who qualified as MAM during the SAM recovery process were still considered SAM and not MAM. AM relapse was defined as a new episode of AM, MAM, or SAM after an initial episode of AM, MAM, or SAM, respectively. Recovery from AM, MAM, or SAM was defined as achieving a normal nutritional status (defined as MUAC > 125 mm and WLZ > −2 and absence of edema) within 3 months after the initial diagnosis by the study field team.

To measure treatment coverage, we used the number of AM cases (cross-sectional study) or AM episodes enrolled in a CMAM program (longitudinal study) as denominator, based on the anthropometric data collected during the respective studies. CMAM enrollment was based on caregiver recall. Receipt of Plumpy’Nut (Nutriset, Malaunay, France) (as per caregiver recall or mentioned on the CMAM beneficiary card) or inpatient treatment (as per caregiver recall) in the past month were considered appropriate SAM treatments. Appropriate treatments for children with MAM included receipt in the past month (as per caregiver recall or mentioned on a beneficiary card) of either appropriate SAM treatment (since children with MAM might be recovering from SAM) or MAM treatment (including Plumpy’Sup or any micronutrient-fortified blended flour or gruel).

Principal component analysis was used to generate a household wealth status score [17]. We included household-level data on materials used in housing roofs, floors, and walls; occupational status of the housing; the main source of light; and assets owned by 5%–95% of the study sample in the baseline cross-sectional study or at enrollment in the longitudinal study. Tertiles of loadings on the principal component with the highest eigenvalue were used in the analyses. Separate scores were created for the two studies.

### Statistical analysis

All analyses were done using a full intent-to-treat approach. For the program’s impact on the six primary outcomes, we calculated a corrected critical *P* value of 0.016 using the Benjamini–Hochberg method to account for the possibility of false discovery when doing multiple tests with an uncorrected 5% significance level, using the uncorrected *P* values obtained for the six primary outcomes [18]. For all other analyses, the statistical significance was set at 5%. All tests were two-sided. Statistical analyses were conducted using Stata 15.0 (Statacorp, College Station, TX, United States). Following the CONSORT guidelines, we did not statistically test for differences between study groups at baseline (cross-sectional study) or at enrollment (longitudinal study) [19].

#### Cross-sectional study

We used linear and linear probability mixed-effects regression models with robust estimation of standard errors to assess the impact of the intervention on continuous and binary outcomes, respectively [20]. Health center catchment area was used as a random effect. We adjusted all analyses for child sex and age, whether child was the first live birth, and the cluster means of the outcome at baseline. We tested the robustness of our results by rerunning all regression analyses, adjusting for covariates that appeared unbalanced at baseline.

#### Longitudinal study

Since nonlinear associations were found between child age and AM screening (primary outcome), CNS coverage, BCC coverage, SQ-LNS coverage, AM prevalence, and ponderal growth, we used mixed-effects regression models with restricted cubic splines, adjusting for health center catchment area and child as random effects and for month of inclusion, child sex, age splines, whether child was the first live birth, and intervention as fixed effects. We used a chunk (omnibus) Wald test to assess whether the intervention modified the association between the aforementioned outcomes and age by jointly testing the “intervention × cubic age spline” interaction terms.

We purposively placed a knot at 6 months of age to account for the change in intervention and screening criteria for AM at this age. To identify the position of additional knots, we plotted the first derivative of a kernel-weighted local polynomial function against child age and looked at where local maxima and minima appeared. We then plotted fitted values from models using these knots against 95% confidence bands of kernel-weighted local polynomial smoothed values of the observed data. We considered the model with the minimum number of knots for which fitted values remained inside the 95% confidence bands. Knots at 3 and 6 months were required for both AM prevalence and WLZ analyses, and an additional knot at 12 months was required for the analysis of WLZ only. For AM screening, CNS coverage, BCC coverage, and SQ-LNS coverage, we could not define adequate knots with this method and needed to let the model automatically generate up to seven knots.

Impact on AM incidence (primary outcome), MAM and SAM incidence, relapse rate, and longitudinal prevalence were estimated using a mixed-effects Poisson regression model with robust estimation of standard errors, with health center as random effect and with child sex, whether the child was a first live birth, month of inclusion, and intervention as fixed effects.

We used linear probability mixed-effects regression models with robust estimation of standard errors to assess the impact of the intervention on the binary AM treatment outcomes (including AM treatment coverage, primary outcome) and a linear mixed-effects regression model to assess the impact of the intervention on episode length [20]. Impact on MUAC gain was estimated using a linear mixed-effects regression model, testing the interaction of intervention with age. All these models included the health center catchment area and the child as a random effects and month of study enrollment, child sex, child age, whether the child was the first live birth, and intervention as fixed effects. In cases when the model did not converge, we removed the child random effect.

We conducted multiple imputation of missing longitudinal outcome data using a 2-fold fully conditional specification algorithm [21,22], which imputes missing values under the missing at random assumption, respecting the temporal ordering of observations. This extension of the standard fully conditional specification was developed for longitudinal study designs with repeated measurements [23]. The information on which we conditioned the imputed values consisted of health center catchment area, child sex, whether the child was the first live birth, child age, and maternal height. The longitudinal analyses were conducted on imputed datasets generated by 50 iterations using the “mi estimate” commands in Stata. As for the cross-sectional analyses, we tested the robustness of our results by further adjusting regression analyses for covariates that appeared unbalanced at enrollment.

#### Exploratory analyses

We conducted subgroup analyses in children below and above 6 months of age because this was the age at which infants became eligible to receive the SQ-LNS and the age at which the use of MUAC for AM screening starts. Separate analyses were also conducted for the MAM and SAM treatment outcomes because two distinct treatment protocols are used in Burkina Faso for these two conditions. These analyses were meant to help explain AM treatment outcome results but were not powered to show statistical significance for potentially meaningful differences. All exploratory analyses were conducted for both the repeated cross-sectional study and the longitudinal study.

### Study registration and ethics

The study was registered on clinicaltrials.org (NCT02245152, September 16, 2014), and the protocol was published [7]. The study protocol (as well as an amendment adjusting the eligible age for the longitudinal study prior to starting enrollment for this study) were approved by the National Ethics Committee of the Ministry of Health in Burkina Faso (#2014-9-113) and the institutional review board at the International Food Policy Research Institute (IRB #00007490).

Prior to study inclusion in the baseline and endline surveys and prior to the longitudinal study, information on the study was given orally and in writing to caregivers of potentially eligible children, and informed consent was documented through signature, or fingerprint for illiterate caregivers. Children with danger signs requiring immediate medical care (such as altered consciousness, continuous vomiting, refusal to eat and drink, convulsions) and children with AM, anemia (measured during cross-sectional surveys only), or malaria (measured during longitudinal survey only) at the time of the survey were referred to the nearest health center. Related treatment costs, if any, were paid by the study team.

## Results

### Cross-sectional study

#### Participants’ trial profile and characteristics

**Fig 2:**
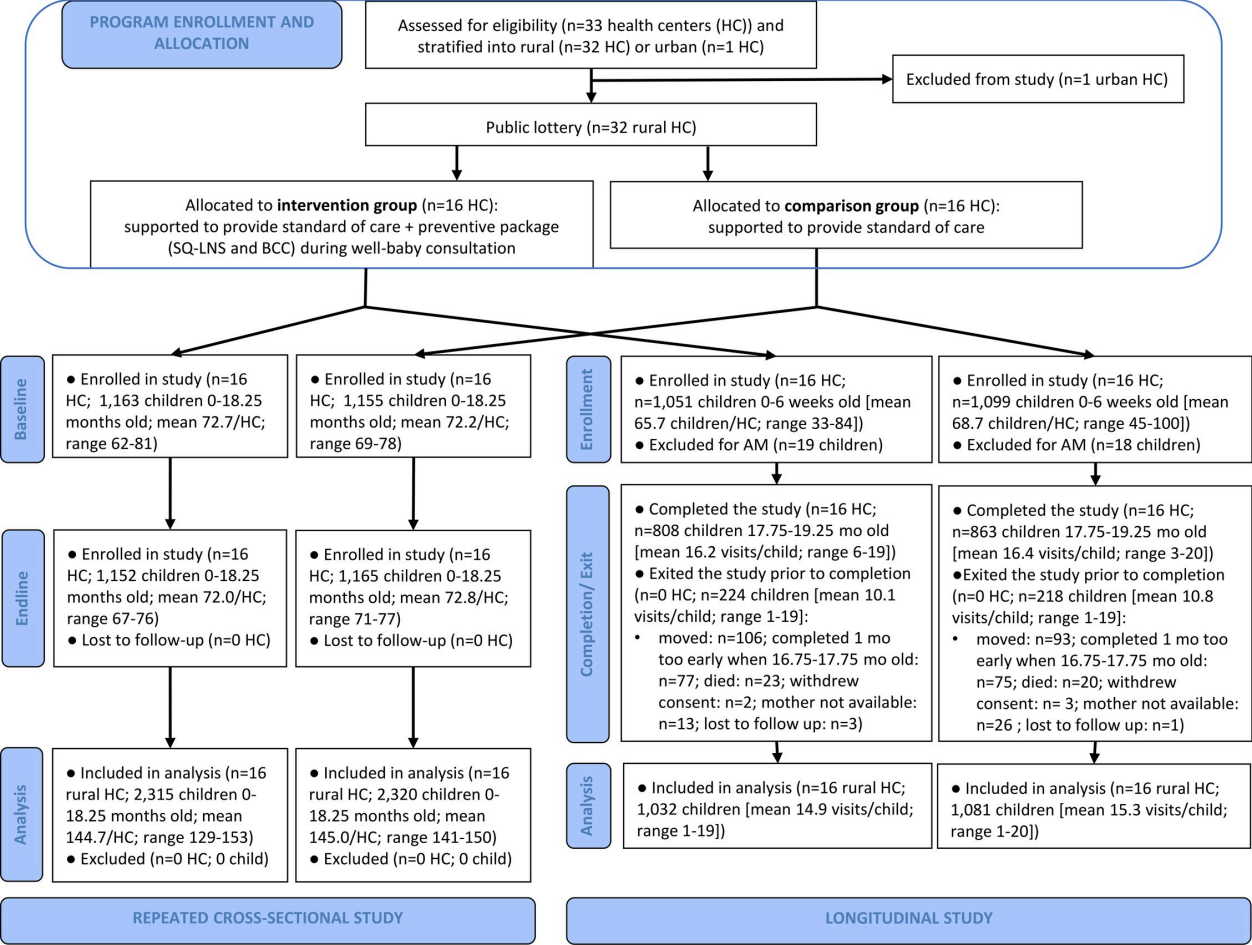
Trial profile for repeated cross-sectional study and longitudinal study. AM was defined as WLZ < −2 at enrollment and at the first follow-up. AM, acute malnutrition; BCC, behavior change communication; HC, health center; SQ-LNS, small-quantity lipid-based nutrient supplement; WLZ, weight-for-length z-score.

The baseline and endline cross-sectional surveys were conducted from October to December 2014 and from October to December 2016, respectively. The cross-sectional study enrollment met the expected number of children per study group at both baseline and endline (Fig 2). None of the enrolled clusters were lost to follow-up. Overall, baseline characteristics were balanced across study groups (Table 2), with a few exceptions. On average, children in the intervention study group lived 2 km closer to the health center than control children. They were also less likely to have been exclusively breastfed (44% intervention versus 54% comparison) or to have consumed iron-rich foods (14% intervention versus 19% comparison) in the previous 24 hours.

**Table 2:** Baseline (cross-sectional study) and enrollment (longitudinal study) sample characteristics by study group.

		Cross-sectional study		Longitudinal study	
		Comparison	Intervention	Comparison	Intervention
Cluster characteristics		*n* = 16	*n* = 16	*n* = 16	*n* = 16
Number of villages per cluster		3.4 ± 1.6	3.1 ± 1.6	3.4 ± 1.6	3.1 ± 1.6
Household characteristics		*n* = 1,106	*n* = 1,120	*n* = 1,065	*n* = 1,012
Linear distance from household to health center, km^a^		5.2 ± 6.5	3.0 ± 2.8	4.6 ± 6.1	2.8 ± 2.4
Household size, members		7.1 ± 3.7	7.1 ± 3.6	6.5 ± 3.2	6.7 ± 3.4
Relative wealth level					
	Lower	356 (32%)	386 (34%)	316 (30%)	377 (37%)
	Average	370 (34%)	372 (33%)	358 (34%)	334 (33%)
	Higher	379 (34%)	362 (32%)	391 (37%)	301 (30%)
Household food insecurity^b^		538 (49%)	506 (45%)	421 (40%)	418 (41%)
Water and sanitation^c^					
	Improved primary water source	599 (54%)	554 (49%)	567 (53%)	504 (50%)
	Improved sanitation facility	535 (48%)	511 (46%)	638 (60%)	453 (45%)
Head of household characteristics		*n* = 1,106	*n* = 1,120	*n* = 1,065	*n* = 1,012
Age, years		38 ± 11	38 ± 11	37 ± 9.5	37 ± 10
Male		1,102 (100%)	1,115 (100%)	1,061 (100%)	991 (98%)
Never attended formal school		858 (78%)	868 (78%)	843 (79%)	777 (77%)
Main caregiver characteristics		*n* = 1,154	*n* = 1,163	*n* = 1,081	*n* = 1,032
Age, years		26 ± 6.3	26 ± 6.4	26 ± 6.2	26 ± 6.6
Married living with spouse		1,048 (91%)	1,068 (92%)	993 (92%)	938 (91%)
Never attended formal school		976 (85%)	998 (86%)	925 (86%)	864 (84%)
Number of food groups consumed^d^		4.0 ± 1.1	3.9 ± 1.1	3.6 ± 1.2	3.4 ± 1.1
Minimum dietary diversity^e^		321 (28%)	302 (26%)	203 (19%)	154 (15%)
Child characteristics		*n* = 1,155	*n* = 1,163	*n* = 1,081	*n* = 1,032
Age, months		8.9 ± 5.0	8.8 ± 4.9	0.67 ± 0.33	0.66 ± 0.32
Male		593 (51%)	611 (53%)	559 (52%)	517 (50%)
First liveborn		236 (21%)	216 (19%)	205 (19%)	195 (19%)
Initiation of breastfeeding within 24 hours^f^		1,040 (90%)	1,077 (93%)	820 (95%)	810 (95%)
		*n* = 392	*n* = 388		
Exclusively breastfed^g^		213 (54%)	172 (44%)	NA	NA
		*n* = 188	*n* = 209		
Timely introduction of (semi-)solid and soft foods^h^		40 (21%)	54 (26%)	NA	NA
		*n* = 762	*n* = 771		
Minimum dietary diversity^i^		120 (16%)	97 (13%)	NA	NA
Minimum meal frequency^j^		331 (43%)	350 (45%)	NA	NA
Minimum acceptable diet^k^		107 (14%)	90 (12%)	NA	NA
Consumption of iron-rich or iron-fortified foods^l^		146 (19%)	105 (14%)	NA	NA
		*n* = 958	*n* = 993		
Anemic^m^		836 (87%)	855 (86%)	NA	NA

Data are mean ± SD or *n* (%).

^a^Calculated using Global Positioning System coordinates collected at household and health center level.

^b^Assessed by Household Food Insecurity Access Scale [24].

^c^Protected well, borehole, pipe, and rain were considered improved water sources, and improved sanitation facility consisted of pit latrine with slab.

^d^Out of the 10 standard food groups used for the minimum dietary diversity for women standard indicator [25].

^e^Consumption of minimally five out of 10 food groups over the past 24 hours, minimum dietary diversity for women standard indicator [25].

^f^Child breastfed within 24 hours after delivery.

^g^Exclusive breastfeeding over the past 24 hours, measured in the subsample of children 0–5 months old [26].

^h^Introduction of (semi)solid or soft foods over the past 24 hours, measured in the subsample of children 6–8 months old [26].

^i^Consumption of minimally four out of seven food groups over the past 24 hours, measured in the subsample of children ≥6 months old [26].

^j^Minimum meal frequency as appropriate for age and breastfeeding status, measured in the subsample of children ≥6 months old [26].

^k^Composite indicator that combines achievement of the minimum dietary diversity and age-appropriate minimum meal frequency, measured in the subsample of children ≥6 months old [26].

^l^Defined by consumption of flesh foods or food fortified with iron over past 24 hours, measured in the subsample of children ≥6 months old [26].

^m^Anemia defined as hemoglobin < 11 g/dL, measured in the subsample of children ≥3 months old.

Abbreviations: NA, not applicable.

#### Impact on coverage of AM screening and preventive services

A significantly greater proportion of children in the intervention compared to the comparison group were screened for AM in the month preceding the endline survey (+18 pp; 95% CI 10–26; *P* < 0.001; primary study outcome) (Table 3).

**Table 3:** Effect of intervention on coverages of AM screening, BCC, and SQ-LNSs in the past month assessed by cross-sectional and longitudinal study.

	Cross-sectional study (endline)					Longitudinal study				
	Comparison	Intervention	Δ^a^ (pp)	95% CI	*P* value	Comparison	Intervention	Δ^b^ (pp)	95% CI	*P* value
All study children	*n* = 1,165^c^	*n* = 1,152^c^				*n* = 18,757^d^	*n* = 17,867^d^			
AM screening coverage (primary outcome)	354 (30%)	550 (48%)	18	10–26	<0.001*	2,275 (12%)	6,200 (35%)	23	17–29	<0.001*
CNS coverage	326 (28%)	566 (49%)	22	13–31	<0.001	2,380 (13%)	6,402 (36%)	23	17–30	<0.001
AM screening coverage through CNS	168 (14%)	458 (40%)	26	16–35	<0.001	1,334 (7.1%)	5,593 (31%)	25	19–31	<0.001
BCC coverage	178 (15%)	313 (27%)	12	4.3–20	0.003	502 (2.7%)	1,665 (9.3%)	6.6	2.6–11	0.002
BCC coverage through CNS	89 (7.6%)	246 (21%)	14	6.7–21	<0.001	313 (1.7%)	1,521 (8.5%)	6.8	2.9–11	0.001
Children <6 months of age	*n* = 379^c^	*n* = 374^c^				*n* = 4,939^d^	*n* = 4,735^d^			
AM screening coverage	46 (12%)	50 (13%)	1.6	−7.4 to 10	0.73	268 (5.4%)	375 (7.9%)	2.5	−2.0 to 7.0	0.27
CNS coverage	140 (37%)	112 (30%)	−6.6	−18 to 4.8	0.26	977 (20%)	949 (20%)	−0.99	−9.8 to 7.8	0.82
AM screening coverage through CNS	35 (9.2%)	43 (12%)	2.4	−6.5 to 11	0.60	215 (4.4%)	329 (7.0%)	2.7	−1.4 to 6.7	0.20
BCC coverage	47 (12%)	47 (13%)	0.65	−6.1 to 7.4	0.85	141 (2.9%)	169 (3.6%)	0.78	−1.0 to 2.6	0.39
BCC coverage through CNS	28 (7.4%)	34 (9.1%)	1.8	−3.4 to 7.1	0.49	99 (2.0%)	140 (3.0%)	0.92	−0.66 to 2.5	0.25
Children ≥6 months of age	*n* = 786^c^	*n* = 778^c^				*n* = 13,818^d^	*n* = 13,132^d^			
AM screening coverage	308 (39%)	500 (64%)	25	16–35	<0.001	2,007 (15%)	5,825 (44%)	30	22–38	<0.001
CNS coverage	186 (24%)	454^e^ (58%)	35	26–45	<0.001	1,403 (10%)	5,453^f^ (42%)	32	25–39	<0.001
AM screening coverage through CNS	133 (17%)	415 (53%)	37	26–47	<0.001	1,119 (8.1%)	5,264 (40%)	33	26–40	<0.001
BCC coverage	131 (17%)	266 (34%)	18	8.6–27	<0.001	361 (2.6%)	1,496 (11%)	8.7	3.5–14	0.001
BCC coverage through CNS	61 (7.8%)	212 (27%)	20	11–29	<0.001	214 (1.6%)	1,381 (11%)	8.9	3.9–14	0.001
SQ-LNS coverage	11 (1.4%)	367 (47%)	46	38–54	<0.001	39 (0.28%)	4,863 (37%)	39	33–45	<0.001
SQ-LNS coverage through CNS	10 (1.3%)	362^g^ (47%)	45	37–54	<0.001	8 (0.06%)	4,835^h^ (37%)	39	33–45	<0.001

Data are *n* (%) unless specified otherwise.

*Statistically significant when considering the critical *P* value calculated using the Benjamini–Hochberg method to account for multiple testing of primary outcomes (*P*_critical_ = 0.016). ICCs for primary outcomes are presented in S1 Table.

^a^Difference between intervention and comparison group in pp analyzed using a mixed-effects linear probability regression model with robust estimation of standard errors, with health center catchment area as random effect.

^b^Difference between intervention and comparison group in pp analyzed using a mixed-effects regression model with robust estimation of standard errors, with restricted cubic spline, with seven knots automatically generated. Models were adjusted for health center catchment area and child as random effects and month of inclusion, age splines, and intervention as fixed effects.

^c^Number of study children.

^d^Number of child visits.

^e^Of whom 353 children were not acutely malnourished at the time of the survey.

^f^Of whom 4,809 child visits were not associated with AM at the time of the visit by the field team.

^g^Of whom 312 children were not acutely malnourished at the time of the survey.

^h^Of whom 4,479 child visits were not associated with AM at the time of the visit by the field team.

Abbreviations: AM, acute malnutrition; BCC, behavior change communication; CNS, well-baby consultation; ICC, intracluster correlation coefficient; pp, percentage points; SQ-LNS, small-quantity lipid-based nutrient supplement.

These results were supported by the observed greater attendance at CNS (+22 pp; 95% CI 13–31; *P* < 0.001), the higher BCC coverage (+12 pp; 95% CI 4.3–20; *P* = 0.003), and the higher SQ-LNS coverage (+46 pp; 95% CI 38–54; *P* = 0.001 in children 6 months or older, as per protocol) in the intervention group compared to the comparison group (Table 3). Subgroup analyses by child age revealed that the differences in all coverage indicators, including AM screening, were statistically significant only in the subsample of children older than 6 months of age, although by design, SQ-LNS was the only intervention restricted to children older than 6 months of age. As per design, SQ-LNS was distributed through the CNS. Although children and their caregivers could be reached by multiple channels and platforms for AM screening and BCC, most of them were reached through the CNS platform.

#### Impact on AM treatment

There was no impact of the intervention on AM treatment coverage (+8.0 pp; 95% CI 0.09–16; *P* = 0.047, inferior to critical *P* value of 0.016; primary study outcome) (Table 4). Subgroup analyses by type of AM showed a small significant impact of the program on treatment coverage of MAM cases, with any CMAM product (+8.6 pp; 95% CI 0.17–17; *P* = 0.045) or with an MAM product (+9.0 pp; 95% CI 0.78–17; *P* = 0.032), but that there was no statistically significant effects on SAM treatment coverage. It is worth mentioning that in the cross-sectional endline survey, 28% of the children with MAM and three of the 23 children with SAM in the intervention group had consumed SQ-LNSs in the month preceding the survey rather than, or in addition to, the recommended MAM- or SAM-specific treatment (S2 Table).

**Table 4:** Effect of the intervention on AM treatment coverage assessed by cross-sectional study.

	Baseline		Endline		Δ^a^ (pp)	95% CI	*P* value
	Comparison	Intervention	Comparison	Intervention			
Children with AM at the time of the survey	*n* = 141	*n* = 191	*n* = 149	*n* = 147			
Treatment coverage (primary outcome)^b^	32 (23%)	54 (28%)	28 (19%)	36 (24%)	8.0	0.09–16	0.047*
Received an MAM and/or SAM treatment product in the past month	33 (23%)	57 (30%)	32 (21%)	40 (27%)	7.4	−0.57 to 15	0.069
Children with MAM at the time of the survey	*n* = 117	*n* = 160	*n* = 124	*n* = 124			
Treatment coverage^b^	27 (23%)	47 (29%)	25 (20%)	33 (27%)	8.6	0.17–17	0.045
Received an MAM treatment product	18 (15%)	28 (18%)	19 (15%)	29 (23%)	9.0	0.78–17	0.032
Received an SAM treatment product	12 (10%)	25 (16%)	7 (5.7%)	6 (4.8%)	0.5	−4.0 to 5.0	0.83
Children with SAM at the time of the survey	*n* = 24	*n* = 31	*n* = 25	*n* = 23			
Treatment coverage^b^	5 (21%)	7 (23%)	3 (12%)	3 (13%)	-0.11	−17 to 17	0.99
Received MAM and/or SAM treatment product	6 (25%)	10 (32%)	7 (28%)	7 (30%)	1.8	−22 to 26	0.88

Data are *n* (%) or mean ± SD.

*Not statistically significant when considering the critical *P* value calculated using the Benjamini–Hochberg method to account for multiple testing of primary outcomes (*P*_critical_ = 0.016). ICCs for primary outcomes are presented in S1 Table.

^a^Difference between intervention and comparison group expressed in pp analyzed using a mixed-effect linear probability model with robust estimation of standard errors, with health center as random effect and child sex, child age, whether the child was a first live birth, intervention, and the cluster means of the outcome at baseline as fixed effects.

^b^Treatment coverage is defined by children with MAM receiving an MAM treatment product or an SAM treatment product and children with SAM receiving an SAM treatment product in the past month.

Abbreviations: AM, acute malnutrition; ICC, intracluster correlation coefficient; MAM, moderate AM; pp, percentage points; SAM, severe AM.

#### Impact on child AM

The intervention did not have a significant impact on the prevalence of either AM (primary outcome), MAM, SAM, weight for length, or MUAC (Table 5).

**Table 5:** Effect of the intervention on AM outcomes assessed by cross-sectional study.

	Baseline		Endline		Δ	95% CI	*P* value
	Comparison	Intervention	Comparison	Intervention			
	*n* = 1,153	*n* = 1,160	*n* = 1,165	*n* = 1,151			
AM prevalence (primary outcome)	141 (12%)	191 (16%)	149 (13%)	147 (13%)	−0.46^a^	−4.4 to 3.5	0.82*
MAM prevalence	117 (10%)	160 (14%)	124 (11%)	124 (11%)	0.39^a^	−3.3 to 4.1	0.84
SAM prevalence	24 (2.1%)	31 (2.7%)	25 (2.2%)	23 (2.0%)	−0.34^a^	−1.4 to 0.77	0.55
WLZ	−0.58 ± 1.1	−0.76 ± 1.2	−0.57 ± 1.1	−0.65 ± 1.1	−0.014^b^	−0.12 to 0.09	0.79
	*n* = 746	*n* = 759	*n* = 770	*n* = 764			
MUAC^c^, mm	138 ± 11	135 ± 11	136 ± 10	136 ± 10	−0.13^b^	−1.8 to 1.6	0.88

Data are *n* (%) or mean ± SD.

*Not statistically significant when considering the critical *P* value calculated using the Benjamini–Hochberg method to account for multiple testing of primary outcomes (*P*_critical_ = 0.016). ICCs for primary outcomes are presented in S1 Table.

^a^Difference between intervention and comparison group expressed in percentage points analyzed using a mixed-effect linear probability model with robust estimation of standard errors, with health center as random effect and child sex, child age, whether the child was a first live birth, intervention, and the cluster means of the outcome at baseline as fixed effects.

^b^Difference between intervention and comparison analyzed using a linear mixed model with health center as random effect and child sex, child age, whether the child was a first live birth, intervention, and the cluster means of the outcome at baseline as fixed effects.

^c^Measured in the subsample of children ≥6 months old.

Abbreviations: AM, acute malnutrition; ICC, intracluster correlation coefficient; MAM, moderate AM; MUAC, mid-upper arm circumference; SAM, severe AM; WLZ, weight-for-length z-score

#### Robustness analyses

After adjusting the analyses for distance to health center (the characteristic that appeared to be unbalanced between groups at baseline), the small impacts of the program on treatment coverage of MAM cases with any CMAM product or with an MAM product were not significant anymore (S3 Table). The other findings were not altered (S3 Table, S4 Table, S5 Table).

### Longitudinal study

#### Participants’ trial profile and characteristics at enrollment

Starting in March 2015, the study enrolled all newborns until the expected sample size was reached (August 2015). The longitudinal study was completed in February 2017. The enrollment fell short by 24 children in the intervention group, but 25 more children were enrolled in the comparison group (Fig 2). None of the enrolled clusters were lost to follow-up. A third of the attrition (*n* = 152, 7.2% of the study sample) was due to children exiting the study just 1 month prior to study completion, because of a misunderstanding by the study field team at early stages of the exit process. The rest of the attrition (*n* = 290, 14% of the study sample) was mostly due to moving out of the study area (*n* = 199) or child death (*n* = 43). The proportion of enrolled children lost to follow-up was balanced across study groups. The study team conducted, on average, 15 out of the 19 expected visits per child. Baseline characteristics were generally well balanced across study groups (Table 2). Children in the intervention group lived 2 km closer to the health center than children in the comparison group. Intervention households were slightly less well-off compared to the comparison group households (37% versus 30% belonged to the lower wealth level).

#### Impact on coverage of AM screening and preventive services

**Fig 3:**
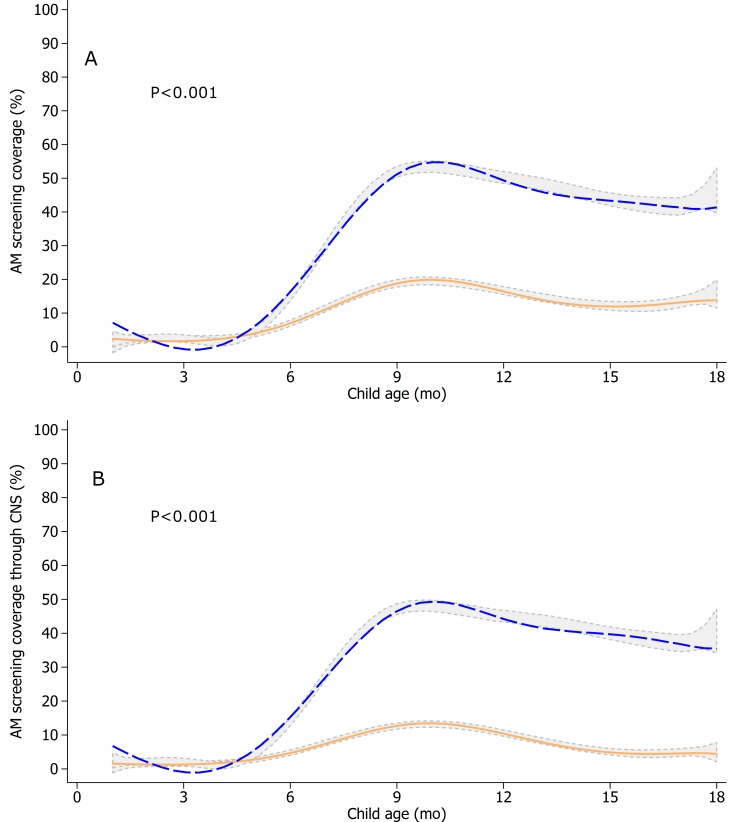
AM screening coverage by any actor (A) and through CNS (B) in the longitudinal study by age and by study group. Monthly screening was defined as MUAC measured (≥6 months old) and/or weight and length measured (all ages) in the past month, as per caregiver recall. The orange solid line represents fitted values for the comparison group. The blue dashed line represents fitted values for the intervention group. Gray areas represent 95% confidence bands of kernel-weighted local polynomial smoothened values by study group using the observed data. Both analyses were based on *n* = 18,757 child visits in the comparison group and *n* = 17,867 child visits in the intervention group. Mixed-effects regression models with restricted cubic splines (seven knots automatically generated) were used, with health center catchment area and child as random intercepts and month of inclusion, child sex, age splines, whether the child was a first live birth, and intervention as fixed effects. A chunk Wald test was used to test the “age splines × intervention” interaction terms (*P* values shown). AM, acute malnutrition; MUAC, mid-upper arm circumference.

The intervention significantly increased monthly screening for AM (+23 pp; 95% CI 17–29; *P* < 0.001, primary outcome) (Table 3), but as documented in the cross-sectional study, subgroup analyses revealed that the effect was limited to children 6 months and older (Fig 3).

Also as shown in the cross-sectional study, the AM coverage impacts were supported by the significant increases in participation in CNS (+23 pp; 95% CI 17–30; *P* < 0.001) and the greater BCC (+6.6 pp; 95% CI 2.6–11; *P* = 0.002) and SQ-LNS coverage (+39 pp in children above 6 months of age; 95% CI 33–45; *P* < 0.001) (Table 3, S1 Fig, S2 Fig, S3 Fig). Impacts on all the coverage outcomes were limited to children aged 6 months and older. Nearly all SQ-LNS was received by intervention children older than 6 months through the CNS, as per design. Consistent with cross-sectional study findings, the majority of children received AM screening, and their caregivers received BCC at the CNS.

#### Impact on AM treatment

Similar to the findings from the cross-sectional study, the impact of the intervention on AM treatment coverage was not statistically significant (+7.7 pp; 95% CI −1.2 to 17; *P* = 0.090; primary outcome) in the longitudinal study (Table 6). On secondary outcomes, the intervention had a statistically significant positive impact on the initiation of treatment in the subsample of AM episodes for which the child was enrolled in a CMAM program (+13 pp; 95% CI 1.3–24; *P* = 0.029) but no impact on recovery or episode length. Subgroup analyses revealed that treatment initiation for children with MAM enrolled in a CMAM program was significantly higher in the intervention group relative to the comparison group (+12 pp; 95% CI 0.090–24; *P* = 0.048), but treatment coverage, though slightly higher, was not significant (+7.0 pp; 95% CI −0.39 to 14; *P* = 0.064). No program impact was found for SAM.

**Table 6:** Effect of the intervention on CMAM enrollment, treatment, and recovery outcomes for AM episodes in the longitudinal study.

	Comparison	Intervention	Δ^a^ (pp)	95% CI	*P* value
AM episodes	*n* = 1,401	*n* = 1,275			
Enrolled in CMAM	424 (30%)	398 (31%)	0.32	−6.2 to 6.8	0.92
	*n* = 424	*n* = 398			
Treatment coverage (primary outcome)^b^	93 (22%)	118 (30%)	7.7	−1.2 to 17	0.090*
Treatment initiated^c^	239 (56%)	276 (69%)	13	1.3–24	0.029
Recovery within 3 months after enrollment	350 (83%)	328 (82%)	−0.15	−6.3 to 6.0	0.96
Length of enrolled episodes, days	73 ± 61	67 ± 51	−5.9^e^	−15 to 3.3	0.21
MAM episodes	*n* = 1,143	*n* = 1,021			
Enrolled in CMAM	338 (30%)	318 (31%)	1.1	−5.8 to 8.0	0.76
	*n* = 338	*n* = 318			
MAM treatment coverage^b^	40 (12%)	61 (19%)	7.0	−0.39 to 14	0.064
MAM treatment initiated^c^	123 (37%)	155 (49%)	12	0.090–24	0.048
Recovery within 3 months after enrollment	301 (89%)	280 (88%)	−0.74^d^	−6.9 to 5.4	0.81
Length of enrolled episodes, days	57 ± 48	56 ± 40	−1.3^e^	−11 to 8.3	0.79
SAM episodes	*n* = 370	*n* = 329			
Enrolled in CMAM	131 (35%)	119 (36%)	1.1	−11 to 13	0.85
	*n* = 131	*n* = 119			
SAM treatment coverage^b^	15 (11%)	14 (12%)	0.42	−9.5 to 10	0.93
SAM treatment initiated^c^	47 (36%)	51 (43%)	5.7^d^	−8.9 to 20	0.44
Recovery within 3 months after enrollment	99 (76%)	88 (74%)	−1.7	−15 to 11	0.80
Length of enrolled episodes, days	75 ± 60	75 ± 51	−0.37^e^	−19 to 18	0.97

Data are *n* (%) or mean ± SD.

*Not statistically significant when considering the critical *P* value calculated using the Benjamini–Hochberg method to account for multiple testing of primary outcomes (*P*_critical_ = 0.016). ICCs for primary outcomes are presented in S1 Table.

^a^Difference between intervention and comparison group expressed in pp analyzed using a mixed-effect linear probability regression model with robust estimation of standard errors, with health center and child as random effects and child sex, child age at the start of the episode, whether the child was a first live birth, and month of inclusion as fixed effects, unless specified otherwise.

^b^Treatment coverage defined as the proportion of children with AM, MAM, or SAM that received continuous treatment from CMAM enrollment onwards over the total number of children with AM, MAM, or SAM, respectively, enrolled in CMAM.

^c^Treatment initiated implies that children with AM received either an MAM or SAM treatment, children with MAM received MAM treatment, and children with SAM received SAM treatment.

^d^Child random effect was removed from the model to solve convergence issues.

^e^Difference in mean episode length (days) between intervention and comparison group analyzed using a linear mixed-effects regression model with health center as random effect and child sex, child age at the start of the episode, whether the child was a first live birth, and month of inclusion as fixed effects. Child random effect was removed from the model to solve convergence issues.

Abbreviations: AM, acute malnutrition; CMAM, community management of AM; ICC, intracluster correlation coefficient; MAM, moderate AM; pp, percentage points; SAM, severe AM

#### Impact on child AM

There was no statistically significant impact of the intervention on the incidence of AM (Table 7), irrespective of whether we considered the first episode (primary outcome) or all episodes over the study period. Similarly, there was no statistically significant impact on the relapse rate of AM. This lack of impact remained when restricting these analyses to children with MAM or SAM (S6 Table).

**Table 7:** Effect of the intervention on the incidence, relapse, and longitudinal prevalence of AM assessed by longitudinal study.

	Comparison	Intervention	IRR/RR	95% CI	*P* value
First episode of AM^a^					
*N* of children	1,081	1,032			
*N* of first episodes/time at risk^b^, child-years	719/1,001	675/948			
Incidence (primary outcome)	0.72	0.71	0.98^c^	0.75–1.3	0.88*
All episodes of AM					
*N* of children	1,081	1,032			
*N* of all episodes/time at risk^d^, child-years	1,401/1,389	1,275/1,338			
Incidence	1.0	0.95	0.90^c^	0.69–1.2	0.45
Relapse episodes of AM					
*N* of children	675	639			
*N* of relapse episodes/time at risk^e^, child-years	682/389	600/390			
Relapse incidence	1.8	1.5	0.88^c^	0.71–1.1	0.21
Longitudinal prevalence AM					
*N* of children	1,081	1,032			
Time with AM/follow-up time, child-years	187/1,577	167/1,505			
Prevalence	12	11	0.91^f^	0.76–1.1	0.32

*Not statistically significant when considering the critical *P* value calculated using the Benjamini–Hochberg method to account for multiple testing of primary outcomes (*P*_critical_ = 0.016). ICCs for primary outcomes are presented in S1 Table.

^a^AM defined by weight-for-length z-score < −2 (all ages), mid-upper arm circumference < 125 mm (≥6 months old), or presence of bilateral pitting edema (all ages).

^b^Time at risk included all consecutive days before the first episode of AM.

^c^IRR analyzed using a mixed-effects Poisson regression model with health center as random effect and child sex, whether the child was a first live birth, month of inclusion, and intervention as fixed effects.

^d^Time at risk included all consecutive days before, between, and after episodes of AM.

^e^Time at risk included all consecutive days before, between, and after episodes of AM, starting after a first episode of AM.

^f^RR analyzed using a mixed-effects Poisson regression model with health center as random effect and child sex, whether the child was a first live birth, month of inclusion, and intervention as fixed effects.

Abbreviations: AM, acute malnutrition; ICC, intracluster correlation coefficient; IRR, incidence rate ratio; RR, risk ratio

We did not find a detectable impact of the intervention on the longitudinal prevalence of AM, MAM, and SAM (Table 7, S6 Table). On average, AM prevalence appeared to decrease faster with age in children 10 months and older in the intervention group relative to the comparison group, but the interaction between the intervention and age on AM prevalence was not statistically significant (*P* = 0.28) (S4 Fig). We found similar results for changes in WLZ increments (*P* = 0.058; S5 Fig) but statistically significant positive effects on MUAC gain (*P* < 0.001; S6 Fig) in children in the intervention group relative to the comparison group.

#### Robustness analyses

Adjusting further for distance to the health center and relative wealth (the characteristics that appeared to be unbalanced between groups at enrollment) led to similar findings (S4 Table, S7 Table, S8 Table), with the exception of the impact estimate for treatment coverage of children with MAM: the coefficient changed only slightly, but the estimate was statistically significant (+7.4 pp; 95% CI 0.53–14; *P* = 0.035; S7 Table).

#### Harms

As stated above, the intervention did not have any significant negative impacts on AM incidence or longitudinal prevalence (Table 7). The proportion of enrolled children who died during the study appeared similar in the intervention group (23 of 1,051) and in the comparison group (20 of 1,099; Fig 2). We did not have the statistical power to assess differences in this outcome.

## Discussion

Integrating a preventive package (age-appropriate BCC on nutrition, health, and hygiene practices and SQ-LNS) into screening for AM at CNS at health centers in Burkina Faso had a large significant impact on AM screening coverage. This was observed in both the cross-sectional (+18 pp) and the longitudinal study (+23 pp). Impacts on AM treatment coverage (about 8 pp), however, were not statistically significant in either study. No impact was found on overall AM prevalence or incidence.

We observed positive impacts on several secondary indicators, including coverage of CNS, BCC, and SQ-LNS in the intervention group among children older than 6 months (both studies). The longitudinal study also revealed a significant impact on the initiation of AM treatment (+13 pp) but no impact on recovery, episode length, and relapse rate. A significant impact was also observed in the longitudinal study on MUAC gain.

### Impacts on coverage of AM screening, BCC, and SQ-LNS

Our results show that incorporating preventive interventions into AM screening acted as a powerful incentive for caregivers to participate in AM screenings, thus confirming our study hypothesis. The program’s preventive package resulted in a much higher CNS coverage and, as a result, greater AM screening, BCC, and SQ-LNS coverage. This is in line with previous studies showing that demand-side incentives are effective in increasing health-seeking for children in low- and middle-income countries [27,28]. The impact on AM screening coverage was statistically significant in our sample as a whole, but subgroup analyses revealed that the impact was limited to children 6 months or older, which corresponds to the age at which they became eligible for SQ-LNS. This finding confirms the importance of the SQ-LNS supplement as an incentive to participate in AM screening. The findings are also consistent with results from a randomized controlled trial on MAM treatment effectiveness in Burkina Faso, which showed that the provision of food supplements (fortified corn soy blend or locally produced fortified spread) with generic nutrition advice at the health center resulted in a significantly lower proportion of children who missed four or more consecutive visits (4.0% and 6.8%, respectively) than providing intensive, enhanced BCC without food supplements (18.5%) [29]. In addition, the conditionality of AM screening for the child to receive SQ-LNS (if found non-AM) is likely to have contributed to the near-universal AM screening at CNS in the intervention group. For example, AM screening was reported at 97% of all CNS visits documented in the intervention group in the longitudinal study (5,264 out of 5,453 CNS visits, Table 3), whereas the percentage in the comparison group was 80%. Also, health staff largely followed the study protocol for SQ-LNS distribution as shown in the longitudinal study, in which 93% of caregivers of non-AM children 6 months of age or older received SQ-LNS when attending CNS (4,479 out of 4,809 visits, longitudinal study, Table 3, footnotes f and h). Of potential concern, however, is the finding that 28% of children with MAM reportedly received SQ-LNS at CNS in the month preceding the endline survey. From information available on the beneficiary cards, we were able to verify that most of these children had not been diagnosed with AM at the time of their CNS visit when they were screened and given the SQ-LNS (S2 Table). Given the time lag between AM screening at CNS and the anthropometric assessment by the research team (0–30 days), we cannot ascertain whether the children identified as MAM by our research team were misdiagnosed as non-MAM at the time of measurement by the health worker because of measurement error or whether the child was indeed non-MAM at the time of the CNS visit and had become MAM during the interval between the CNS visit and our research team measurement. We are therefore unable to determine whether the receipt of SQ-LNS by these AM children was the result of a measurement error or a deviation from protocol.

Implementation of the age-appropriate, small-group BCC preventive intervention appears to have been a lot less successful than SQ-LNS distribution. Overall, fewer than half of all caregivers received any type of BCC while attending the CNS. The impact of the program on BCC coverage was thus smaller compared to that for SQ-LNS (+12 to 14 pp in the cross-sectional and +7 pp in the longitudinal study, respectively). Low incorporation of BCC into the CNS might have been due to health workers’ high workloads, poor motivation, poor supervision, and/or lack of appropriate incentives to do the work. These factors have been shown to contribute to poor BCC implementation in other contexts [30]. Another factor that may have affected the health workers’ motivation to organize BCC at the CNS is low caregiver demand. Experience from large-scale counseling projects for IYCF suggests that caregivers do not always appreciate the importance of nutrition and health counseling, and promotional activities to stimulate demand for this type of information are recommended [31]. Radio broadcasts and village nutrition committees established and trained by HKI in both study groups were intended to raise community awareness about the importance of attending the CNS but might not have sufficiently addressed the importance of BCC at the CNS sessions. This could have increased demand and helped with holding health staff accountable to organize BCC.

Even though the preventive package increased CNS coverage and as a result increased the BCC and SQ-LNS coverage and AM screening, the intervention did not overcome all barriers to the regular use of preventive services at the health facility. Every month, approximately half of the caregivers of SQ-LNS-eligible children failed to attend the CNS, resulting in limited exposure of children to the combined preventive and screening services. Even more severe is the problem with caregivers of young infants (<6 months of age) whose attendance at CNS ranged from 20% to 37% (Table 3). Half of these caregivers believed that it was not necessary for their child to attend the CNS (S9 Table), demonstrating a lack of awareness of CNS services and their importance for monitoring the health and nutrition of young infants. Coverage of AM screening among children under 6 months of age who did attend the CNS was also low (e.g., only 43 out of 112 children or 38% who attended CNS in the intervention group at endline were screened for AM, Table 3). Health workers may have prioritized screening for AM in children older than 6 months during CNS for a variety of reasons. They may have considered AM too rare a condition in this age group to be worth screening for. Health workers may also have been confused by the national protocol, which does not provide any concrete guidance for MAM treatment for children younger than 6 months (ready-to-use supplementary food is not recommended for children in this age group); in addition, in case SAM is detected, the protocol prescribes referring these young infants to the district hospital for inpatient treatment [11]. Finally, health workers may have felt that the diagnostic for AM in this age group was too complex, as it requires measuring both weight and length.

### Impact on AM treatment

In spite of the large impact of the intervention on AM screening coverage, we saw only a small and nonsignificant impact of approximately 8 pp on AM treatment coverage in both the cross-sectional and the longitudinal studies. The longitudinal study also showed significant but small impacts on AM (+13 pp) and MAM (+12 pp) treatment initiation and on MAM treatment coverage in the cross-sectional study (+8.6 pp, not significant anymore in robustness analysis), and no impacts on other AM treatment outcomes such as length of treatment, recovery rate, or relapse rate. These results were unexpected, given our main hypothesis that greater AM screening coverage would lead to a positive impact on treatment coverage through appropriate referral, follow-up, treatment initiation, treatment uptake, and treatment completion. The limited impact on AM treatment indicators shows that major barriers to treatment remained largely unaddressed by the PROMIS intervention and that key problems with implementation of CMAM programs persisted [6]. Through its technical support to the effective implementation of the national CMAM protocol at the health center and community level, the project was designed to address some of the key barriers to AM treatment in both study groups. For example, BCC and community engagement aimed to address the lack of community and caregiver recognition of child AM and awareness of the CMAM program. The CMAM-related training and supervision of health staff was implemented to address the potential failure by health staff to identify and enroll children with AM who were seeking treatment at the health center [6]. In addition, the preventive package in the intervention group sought to address the barrier related to the perceived absence of attention provided at the health center to caregivers whose child was identified as well-nourished and therefore not eligible for CMAM enrollment and treatment. On the other hand, the cost of transportation to the health center and the opportunity cost of weekly or fortnightly visits to CMAM consultation were not addressed by PROMIS. This could have been done by bringing services closer to villages or organizing transportation or through monetary compensation. The opportunity cost was apparently high in the study area; lack of time ranked among the highest reasons reported by caregivers of children 6 months or older for not attending the monthly CNS visits at the health center (S9 Table). CMAM treatment for SAM or MAM children also requires, respectively, weekly and fortnightly visits to the health center. This can pose a significant time and/or financial burden on caregivers, especially if health centers are far from their home. A study in Mali showed that bringing SAM treatment to the community considerably increased treatment coverage and reduced the proportion of cases who missed two or more consecutive appointments by half [32].

Further research is needed to test solutions to reduce opportunity costs and other barriers to AM treatment and especially to ensure that improvements in screening translate into higher rates of AM treatment completion and reductions in AM prevalence and incidence.

### Impact on child AM

The preventive intervention package did not lower AM incidence or prevalence, with the exception of a small, statistically significant positive impact on MUAC gain. A reduction in AM prevalence could have occurred through a reduction in AM incidence and, to a lesser extent, through a shortening of AM episode length. Reducing episode length requires effective AM treatment, but we found only a small, nonsignificant impact of the intervention on AM treatment.

An important pathway for lowering AM prevalence would be through reductions in AM incidence (prevention pathway). Lowering AM incidence would have required the prevention of new AM cases through ensuring adequate dietary intake and the prevention and treatment of infectious diseases. As discussed above, exposure to age-appropriate BCC on nutrition, health, and hygiene practices was low in the intervention group, even after children reached 6 months of age (34%, cross-sectional study; 11%, longitudinal study). Consequently, the impact of the intervention on nutrition, health, and hygiene knowledge and practices and associated prevention of child illnesses appears to have been limited. Detailed impact results on these indicators will be published separately.

The second hypothesized pathway to lower AM incidence was through the preventive effect of SQ-LNS itself (see Fig 1). A recent meta-analysis showed that SQ-LNS with complementary feeding reduces AM prevalence by 15% [33]. However, impact studies using SQ-LNS in similar populations of similar age in Burkina Faso showed mixed effects of SQ-LNS on AM indicators; one study (included in the meta-analysis cited above) found a 4.8 pp (36%) reduction of wasting when SQ-LNS was combined with treatment of diarrhea and malaria [34]; and a second study found no impact of SQ-LNS on AM prevalence or WLZ [35]. In the Mali companion study to our Burkina Faso evaluation, SQ-LNS was responsible for a 29% reduction in AM incidence, mostly during the peak of AM observed between 6 and 12 months of age, with an SQ-LNS coverage of 60%–70% at that age [8]. In our study, fewer than half of the caregivers of children older than 6 months reported receiving SQ-LNS in the past month (47%, cross-sectional study; 37%, longitudinal study), and thus, it is possible that for the majority of children, the SQ-LNS did not make a large enough contribution to the nutrient adequacy of their diet to prevent AM.

### Using CNS at the health center to integrate preventive services into AM screening

Taken together, the findings from our study and its companion study in Mali [8] highlight the trade-offs of using community- versus health center–based services for the delivery of AM services. Choosing the CNS as the intervention platform for strengthening AM screening offered several advantages. First, scales and length board were available on site, which allowed health staff to screen children below 6 months of age and to use the full set of criteria for AM screening in children 6 months and older (WLZ, MUAC, and edema). About one-third of children older than 6 months with AM were identified as such by the WLZ criterion only, which implies that these children would have been missed if only MUAC and edema had been used for screening. This is a problem with current indicators used for screening at the community level, as shown in the Mali study.

A second advantage of using the health facility CNS platform for integrating preventive interventions into AM screening is the involvement of professional health staff, which in our study is likely to have contributed to the correct distribution of the SQ-LNS (i.e., to nonmalnourished children 6 months and older). In the study in Mali, much of the SQ-LNS was distributed outside of the monthly meetings, thus missing opportunities for children to be screened. On the other hand, the impact on BCC coverage was two to three times larger in Mali than in Burkina Faso. This suggests that delivery-side barriers to effective implementation of BCC on nutrition, health, and hygiene topics may be more difficult to overcome in the context of health center–based compared to community-based services. This limitation was recognized in Burkina Faso’s current national policy for the scale-up of BCC on optimal IYCF practices, which largely relies on the community approach (mother groups led by community health workers) [36].

A third advantage of using the CNS platform for AM screening is to generate a “one-stop shop,” whereby caregivers who attend can benefit from a variety of other preventive and curative clinical services in one visit to the health center. A potential downside is the risk of overloading the system and possibly lowering the quality of services as a result. In 2010, Burkina Faso only had 6.12 doctors, midwives, and nurses for every 10,000 people [37], far below the recommended number of 44.5 per 10,000 people to ensure universal health coverage and the health targets of the sustainable development goals [38]. These daunting statistics highlight the importance of continuing to assess the operational capacity and feasibility of integrating preventive interventions into CMAM services in the context of CNS platforms operating at health facilities.

### Strengths and limitations

Our study had several strengths. It used a rigorous cluster-randomized design to assess the impact of an intervention implemented through the existing health system. Close collaboration between program implementers and researchers and a strong commitment from the main donor and implementer to document impact allowed us to combine an intervention at scale with the strongest possible study design (a randomized controlled trial) and to produce much-needed strong evidence on strategies to improve CMAM effectiveness. Using a dual cross-sectional and longitudinal study design allowed us to assess the impact on both AM prevalence and incidence and to study the time and age dynamics of outcomes such as program exposure, AM treatment, and recovery. The cross-sectional and longitudinal studies used independently drawn samples, which strengthens the validity of the impacts found across the two designs. Also, the samples were population-based (rather than clinic-based) and representative of the rural population of children aged 0–18 months in the Gourcy health district.

The longitudinal study, however, also had inherent limitations. First, it was a closed cohort enrolled over 6 months, which does not allow us to fully separate age and seasonal effects. This does not bias the impact estimates, however, because both study groups were enrolled over the same time period. Second, the monthly anthropometric measurements by the enumerators and the subsequent referral of children with AM for ethical reasons meant that a screening and referral intervention was effectively implemented in both arms by the research team. This might have attenuated the impact estimates on AM treatment coverage by increasing AM screening and referral in both groups, making it harder to detect the true impact of the intervention on treatment coverage. However, estimates of AM treatment coverage in the two groups were of similar magnitude in the cross-sectional (19%) and longitudinal (22%) studies, as was the program’s impact on AM treatment (around 8% in both studies).

### Conclusion

To our knowledge, no previous study tested and evaluated, using a rigorous research design, the potential benefits of bringing prevention into CMAM programming. Our findings show the incentive power of preventive services, especially SQ-LNS, in increasing AM screening coverage using a health facility–based CNS platform. The lack of impact on AM treatment coverage and AM incidence and prevalence, however, indicates that important barriers to the optimal delivery and utilization of these health center–based CMAM services remained in the study areas. These unaddressed barriers limited the potential of the preventive package to reduce AM incidence and prevented the observed improvements in screening coverage to translate into higher rates of AM treatment and related declines in AM prevalence.

Health center–based services have some advantages over community-based services in that they allow the centralization of multiple services, including bringing AM screening and treatment together, and can potentially improve the accuracy of AM diagnostic and quality of protocol implementation. Given their centralized location, however, health center–based services may reduce caregivers’ participation because of distance and related travel and opportunity costs.

In a context in which health center–based CMAM services are established and viable, research should focus on testing innovative approaches to tackle the key barriers to utilization of the services and to strengthen the BCC component of the intervention to sensitize the population about the prevention and treatment of AM. More research is also needed on complementary approaches to bring integrated preventive and AM treatment services closer to the community while ensuring optimal quality of implementation and service delivery.

## Supporting information

S1 CONSORT Checklist CONSORT extension for cluster trials checklist. (DOCX)

S1 Table Intracluster correlation coefficients for primary outcomes. (DOCX)

S2 Table Recorded or reported AM status at the time of SQ-LNS supply for children identified with AM, MAM, or SAM at the time of the survey, cross-sectional study. AM, acute malnutrition; MAM, moderate AM; SAM, severe AM; SQ-LNS, small-quantity lipid-based nutrition supplement.
(DOCX)

S3 Table Effect of the intervention on AM treatment coverage assessed by cross-sectional study (robustness analysis adjusting further for distance to health center). AM, acute malnutrition.
(DOCX)

S4 Table Effect of intervention on coverages of AM screening, BCC, and SQ-LNSs in the past month assessed by cross-sectional and longitudinal study (robustness analysis adjusting further for distance to health center). AM, acute malnutrition; BCC, behavior change communication; SQ-LNS, small-quantity lipid-based nutrient supplement.
(DOCX)

S5 Table Effect of the intervention on AM outcomes assessed by cross-sectional study (robustness analysis adjusting further for distance to health center). AM, acute malnutrition.
(DOCX)

S6 Table Effect of the intervention on the incidence, relapse, and longitudinal prevalence of moderate and severe AM, longitudinal study. AM, acute malnutrition.
(DOCX)

S7 Table Effect of the intervention on CMAM enrollment, treatment, and recovery outcomes for AM episodes in the longitudinal study (robustness analysis adjusting further for distance to health center and relative wealth status). AM, acute malnutrition; CMAM, community management of acute malnutrition.
(DOCX)

S8 Table Effect of the intervention on the incidence, relapse, and longitudinal prevalence of AM assessed by longitudinal study (robustness analysis adjusting further for distance to health center and relative wealth status). AM, acute malnutrition.
(DOCX)

S9 Table Reasons for not attending CNS in the past month, by age range and study group. CNS, well-baby consultation (consultation du nourrisson sain).
(DOCX)

S1 Fig Monthly CNS coverage in the longitudinal study according to age, by study group. Gray areas represent 95% confidence bands of kernel-weighted local polynomial smoothed values by study group using the observed data. The orange solid line represents fitted values for the comparison group. The blue dashed line represents fitted values for the intervention group. Analysis was based on *n* = 18,757 child visits in comparison group and *n* = 17,867 child visits in intervention group. Mixed-effects regression model with restricted cubic splines (7 knots automatically generated) was used, with health center catchment area and child as random intercepts and month of inclusion, child sex, age splines, whether the child was a first live birth, and intervention as fixed effects. A chunk Wald test was used to test the “age splines × intervention” interaction terms (*P* value shown). CNS, well-baby consultation (consultation du nourrisson sain).
(TIF)

S2 Fig Monthly SQ-LNS coverage in the longitudinal study according to the age, by study group. The orange solid line represents fitted values for the comparison group. The blue dashed line represents fitted values for the intervention group. Gray areas represent 95% confidence bands of kernel-weighted local polynomial smoothed values by study group using the observed data. Analysis was based on *n* = 18,757 child visits in comparison group and *n* = 17,867 child visits in intervention group. Mixed-effects regression model with restricted cubic splines (7 knots automatically generated) was used, with health center catchment area and child as random intercepts and month of inclusion, child sex, age splines, whether the child was a first live birth, and intervention as fixed effects. A chunk Wald test was used to test the “age splines × intervention” interaction terms (*P* value shown). SQ-LNS, small-quantity lipid-based nutrition supplement.
(TIF)

S3 Fig Monthly BCC coverage by any actor (A) and through CNS (B) in the longitudinal study according to the age, by study group. The orange solid line represents fitted values for the comparison group. The blue dashed line represents fitted values for the intervention group. Gray areas represent 95% confidence bands of kernel-weighted local polynomial smoothed values by study group using the observed data. Both analyses were based on *n* = 18,757 child visits in comparison group and *n* = 17,867 child visits in intervention group. Mixed-effects regression models with restricted cubic splines (7 knots automatically generated) were used, with health center catchment area and child as random intercepts and month of inclusion, child sex, age splines, whether the child was a first live birth, and intervention as fixed effects. A chunk Wald test was used to test the “age splines × intervention” interaction terms (*P* values shown). BCC, behavior change communication; CNS, well-baby consultation (consultation du nourrisson sain)
(TIF)

S4 Fig Effect modification of the intervention by age on AM prevalence in the longitudinal study. AM is defined by a weight-for-length z-score < −2 (all ages) or mid-upper arm circumference < 125 mm (≥6 months old) or presence of bilateral pitting edema (all ages). The orange solid line represents fitted values for the comparison group. The blue dashed line represents fitted values for the intervention group. Gray areas represent 95% confidence bands of kernel-weighted local polynomial smoothed values by study group using the observed data. Analysis was based on *n* = 18,090 child visits in comparison group and *n* = 17,266 child visits in intervention group. Mixed-effects regression model with robust estimation of standard errors and with restricted cubic splines (knots at 3 and 6 months) was used, with health center catchment area and child as random intercepts and month of inclusion and child sex, age splines, whether the child was a first live birth, and intervention as fixed effects. A chunk Wald test was used to test the “age splines × intervention” interaction terms (*P* value shown). AM, acute malnutrition.
(TIF)

S5 Fig Effect modification of the intervention by age on WLZ in the longitudinal study. The orange solid line represents fitted values for the comparison group. The blue dashed line represents fitted values for the intervention group. Gray areas represent 95% confidence bands of kernel-weighted local polynomial smoothed values by study group using the observed data. Analysis was based on *n* = 18,090 child visits in comparison group and *n* = 17,266 child visits in intervention group. Mixed-effects regression model with restricted cubic splines (knots at 3, 6, and 12 months) was used, with health center catchment area and child as random intercepts and month of inclusion, child sex, age splines, whether the child was a first live birth, and intervention as fixed effects. A chunk Wald test was used to test the “age splines × intervention” interaction terms (*P* value shown). WLZ, weight-for-length z-score.
(TIF)

S6 Fig Effect modification of the intervention by age on MUAC in the longitudinal study. The orange solid line represents fitted values for the comparison group. The blue dashed line represents fitted values for the intervention group. Gray areas represent 95% confidence bands of kernel-weighted local polynomial smoothed values by study group using the observed data. Analysis was based on *n* = 13,073 child visits in comparison group and *n* = 12,485 child visits in intervention group. Linear mixed-effects regression model was used, with health center catchment area and child as random intercepts and month of inclusion, child sex, age splines, whether the child was a first live birth, and intervention as fixed effects. We tested the “age × intervention” interaction term (*P* value shown). MUAC, mid-upper arm circumference.
(TIF)

S1 Text Study protocol for the PROMIS Burkina Faso study approved by national ethics committee. PROMIS, Innovative Approaches for the Prevention of Childhood Undernutrition.
(PDF)

S2 Text Data analysis plan for PROMIS Burkina Faso. PROMIS, Innovative Approaches for the Prevention of Childhood Undernutrition.
(DOCX)

S3 Text French translation of the abstract. (DOCX)

## Abbreviations

AM: acute malnutrition
BCC: behavior change communication
CMAM: community management of acute malnutrition
CNS: well-baby consultation (consultation du nourrisson sain)
HKI: Helen Keller International
ICC: intracluster correlation coefficient
IRR: incidence rate ratio
IYCF: Infant and young child feeding
MAM: moderate acute malnutrition
MUAC: mid-upper arm circumference
pp: percentage points
PROMIS: Innovative Approaches for the Prevention of Childhood Undernutrition
RR: risk ratio
SAM: severe acute malnutrition
SQ-LNS: small-quantity lipid-based nutrient supplement
WLZ: weight-for-length z-score
