# Supplementary material for: Impact on child acute malnutrition of integrating a preventive nutrition package into facility-based screening for acute malnutrition during well-baby consultation: A cluster-randomized controlled trial in Burkina Faso
Source: PLoS Med. 2019 Aug 27;16(8):e1002877. doi: 10.1371/journal.pmed.1002877 (PMC6711504; doi:10.1371/journal.pmed.1002877)
Supplement: S1 Table — (DOCX) [file pmed.1002877.s002.docx]

**S1 Table: Intra-cluster correlation coefficients for primary outcomes**

|  | Endline study | Longitudinal study |
| --- | --- | --- |
| **Gourcy district** |  |  |
| AM screening coverage | 0.078 | 0.115 |
| AM treatment coverage | 0.0036 | 0.031 |
| AM prevalence | 0.0048 | n/a |
| AM incidence | n/a | 0.028 |
| **Comparison** |  |  |
| AM screening coverage | 0.039 | 0.054 |
| AM treatment coverage | 0.0030 | 0.040 |
| AM prevalence | 0.0044 | n/a |
| AM incidence | n/a | 0.044 |
| **Intervention** |  |  |
| AM screening coverage | 0.057 | 0.042 |
| AM treatment coverage | 0.0023 | 0.035 |
| AM prevalence | 0.0039 | n/a |
| AM incidence | n/a | 0.011 |

n/a, non applicable

Abbreviations: AM, acute malnutrition
