# Supplementary material for: Impact on child acute malnutrition of integrating a preventive nutrition package into facility-based screening for acute malnutrition during well-baby consultation: A cluster-randomized controlled trial in Burkina Faso
Source: PLoS Med. 2019 Aug 27;16(8):e1002877. doi: 10.1371/journal.pmed.1002877 (PMC6711504; doi:10.1371/journal.pmed.1002877)
Supplement: S2 Table — AM, acute malnutrition; MAM, moderate AM; SAM, severe AM; SQ-LNS, small-quantity lipid-based nutrition supplement. (DOCX) [file pmed.1002877.s003.docx]

**S2 Table: Recorded or reported AM status at the time of SQ-LNS supply for children identified with AM, MAM or SAM at the time of the endline survey, cross-sectional study**

|  | AM | MAM | SAM |
| --- | --- | --- | --- |
| **Children with AM at the time of the survey** | *n* = 147 | *n* = 124 | *n* = 23 |
| Consumed SQ-LNS in the past month | 38 (26%) | 35 (28%) | 3 (13%) |
| Received SQ-LNS at CNS in the past month | 41 (28%) | 38 (31%) | 3 (13%) |
| **Children with AM who received SQ-LNS in the past month** | *n* = 41 | *n* = 38 | *n* = 3 |
| No beneficiary card, caregiver does not recall result | 12 | 9 | 3 |
| Healthy status recorded on beneficiary card | 23 | 23 | 0 |
| No beneficiary card, caregiver recalls healthy status | 4 | 4 | 0 |
| MAM status recorded on beneficiary card | 1 | 1 | 0 |
| No beneficiary card, caregiver recalls MAM status | 0 | 0 | 0 |
| SAM status recorded on beneficiary card | 0 | 0 | 0 |
| No beneficiary card, caregiver recalls SAM status | 1 | 1 | 0 |

Data are n(%).

Abbreviations: AM, acute malnutrition; MAM, moderate acute malnutrition; SAM, severe acute malnutrition; SQ-LNS, small quantity lipid-based nutrient supplement
