# Supplementary material for: Impact on child acute malnutrition of integrating a preventive nutrition package into facility-based screening for acute malnutrition during well-baby consultation: A cluster-randomized controlled trial in Burkina Faso
Source: PLoS Med. 2019 Aug 27;16(8):e1002877. doi: 10.1371/journal.pmed.1002877 (PMC6711504; doi:10.1371/journal.pmed.1002877)
Supplement: S3 Table — AM, acute malnutrition. (DOCX) [file pmed.1002877.s004.docx]

**S3 Table: Effect of the intervention on acute malnutrition treatment coverage assessed by cross-sectional (robustness analysis adjusting further for distance to health center)**

|  | **Baseline** | |  | **Endline** | |  | **∆^a^ (pp)** | **95% CI** | ***P*-value** |
| --- | --- | --- | --- | --- | --- | --- | --- | --- | --- |
|  | **Comparison** | **Intervention** |  | **Comparison** | **Intervention** |  |  |  |  |
| **Children with AM at the time of the survey** | *n* = 141 | *n* = 191 |  | *n* = 149 | *n* = 147 |  |  |  |  |
| Treatment coverage (primary outcome) ^b^ | 32 (23%) | 54 (28%) |  | 28 (19%) | 36 (24%) |  | 7.9 | -1.3 to 17 | 0.091* |
| Received a MAM and/or SAM treatment product in the past month | 33 (23%) | 57 (30%) |  | 32 (21%) | 40 (27%) |  | 7.3 | -2.2 to 17 | 0.13 |
| **Children with MAM at the time of the survey** | *n* = 117 | *n* = 160 |  | *n* = 124 | *n* = 124 |  |  |  |  |
| Treatment coverage ^b^ | 27 (23%) | 47 (29%) |  | 25 (20%) | 33 (27%) |  | 8.5 | -2.0 to 19 | 0.11 |
| Received a MAM treatment product | 18 (15%) | 28 (18%) |  | 19 (15%) | 29 (23%) |  | 9.2 | -0.21 to 19 | 0.055 |
| Received a SAM treatment product | 12 (10%) | 25 (16%) |  | 7 (5.7%) | 6 (4.8%) |  | 0.30 | -5.5 to 6.1 | 0.92 |
| **Children with SAM at the time of the survey** | *n* = 24 | *n* = 31 |  | *n* = 25 | *n* = 23 |  |  |  |  |
| Treatment coverage ^b^ | 5 (21%) | 7 (23%) |  | 3 (12%) | 3 (13%) |  | -0.17 | -17 to 17 | 0.99 |
| Received MAM and/or SAM treatment product | 6 (25%) | 10 (32%) |  | 7 (28%) | 7 (30%) |  | 1.8 | -22 to 26 | 0.88 |

Data are n(%) or mean ± SD.

* Not statistically significant when considering the critical p-value calculated using the Benjamini-Hochberg method to account for multiple testing of primary outcomes (*P*_critical_= 0.016). ICC for primary outcomes are presented in supplemental table S10

^a^ Difference between intervention and comparison group expressed in percentage point analyzed using a mixed-effect linear probability model with robust estimation of standard errors, with health center as random effect and child sex, child age, whether the child was a first live birth, intervention, distance to health center and the cluster means of the outcome at baseline as fixed effects

^b^ Treatment coverage is defined by children with MAM receiving a MAM treatment product or a SAM treatment product and children with SAM receiving a SAM treatment product in the past month

Abbreviations: AM, acute malnutrition; ICC, intracluster correlation coefficient; MAM, moderate acute malnutrition; pp, percentage points; SAM, severe acute malnutrition
