# Supplementary material for: Impact on child acute malnutrition of integrating a preventive nutrition package into facility-based screening for acute malnutrition during well-baby consultation: A cluster-randomized controlled trial in Burkina Faso
Source: PLoS Med. 2019 Aug 27;16(8):e1002877. doi: 10.1371/journal.pmed.1002877 (PMC6711504; doi:10.1371/journal.pmed.1002877)
Supplement: S4 Table — AM, acute malnutrition; BCC, behavior change communication; SQ-LNS, small-quantity lipid-based nutrient supplement. (DOCX) [file pmed.1002877.s005.docx]

**S4 Table: Effect of intervention on coverages of acute malnutrition screening, behavior change communication and small quantity lipid-based nutrient supplements in the past month assessed by cross-sectional and longitudinal (robustness analysis adjusting further for distance to health center)**

|  | **Cross-sectional study (endline)** | | | | |  | **Longitudinal study** | | | | |
| --- | --- | --- | --- | --- | --- | --- | --- | --- | --- | --- | --- |
|  | **Compari-son** | **Interven-tion** | **∆^a^ (pp)** | **95% CI** | ***P*-value** |  | **Compari-son** | **Interven-tion** | **∆^b^ (pp)** | **95% CI** | ***P*-value** |
| **All study children** | *n* = 1,165 ^c^ | *n* = 1,152 ^c^ |  |  |  |  | *n* = 18,757 ^d^ | *n* = 17,867 ^d^ |  |  |  |
| AM screening coverage (primary outcome) | 354 (30%) | 550 (48%) | 17 | (9.8, 25) | <0.001* |  | 2,275 (12%) | 6,200 (35%) | 21 | 14–27 | <0.001* |
| CNS coverage | 326 (28%) | 566 (49%) | 21 | (13, 30) | <0.001 |  | 2,380 (13%) | 6,402 (36%) | 20 | 13–28 | <0.001 |
| AM screening coverage through CNS | 168 (14%) | 458 (40%) | 25 | (16, 34) | <0.001 |  | 1,334 (7.1%) | 5,593 (31%) | 23 | 16–29 | <0.001 |
| BCC coverage | 178 (15%) | 313 (27%) | 12 | (4.4, 19) | 0.0018 |  | 502 (2.7%) | 1,665 (9.3%) | 6.3 | 2.3–10 | 0.002 |
| BCC coverage through CNS | 89 (7.6%) | 246 (21%) | 14 | (6.9, 20) | <0.001 |  | 313 (1.7%) | 1,521 (8.5%) | 6.1 | 2.3–10 | 0.002 |
|  |  |  |  |  |  |  |  |  |  |  |  |
| **Children <6 months of age** | *n* = 379 ^c^ | *n* = 374 ^c^ |  |  |  |  | *n* = 4,939 ^d^ | *n* = 4,735 ^d^ |  |  |  |
| AM screening coverage | 46 (12%) | 50 (13%) | 1.1 | (-7.7, 10) | 0.80 |  | 268 (5.4%) | 375 (7.9%) | 1.3 | -3.2 to 5.9 | 0.57 |
| CNS coverage | 140 (37%) | 112 (30%) | -7.2 | (-18, 4.0) | 0.21 |  | 977 (20%) | 949 (20%) | -4.8 | -15 to 5.6 | 0.38 |
| AM screening coverage through CNS | 35 (9.2%) | 43 (12%) | 2.0 | (-6.8, 11) | 0.65 |  | 215 (4.4%) | 329 (7.0%) | 1.5 | -2.7 to 5.7 | 0.48 |
| BCC coverage | 47 (12%) | 47 (13%) | 0.30 | (-6.1, 6.7) | 0.93 |  | 141 (2.9%) | 169 (3.6%) | 0.38 | -1.3 to 2.0 | 0.65 |
| BCC coverage through CNS | 28 (7.4%) | 34 (9.1%) | 1.5 | (-3.4, 6.4) | 0.54 |  | 99 (2.0%) | 140 (3.0%) | 0.49 | -1.1 to 2.1 | 0.54 |
|  |  |  |  |  |  |  |  |  |  |  |  |
| **Children ≥6 months of age** | *n* = 786 ^c^ | *n* = 778 ^c^ |  |  |  |  | *n* = 13,818 ^d^ | *n* = 13,132 ^d^ |  |  |  |
| AM screening coverage | 308 (39%) | 500 (64%) | 25 | (16, 34) | <0.001 |  | 2,007 (15%) | 5,825 (44%) | 28 | 20–36 | <0.001 |
| CNS coverage | 186 (24%) | 454 (58%) | 35 | (26, 44) | <0.001 |  | 1,403 (10%) | 5,453 (42%) | 29 | 22–37 | <0.001 |
| AM screening coverage through CNS | 133 (17%) | 415 (53%) | 36 | (26, 47) | <0.001 |  | 1,119 (8.1%) | 5,264 (40%) | 30 | 22–38 | <0.001 |
| BCC coverage | 131 (17%) | 266 (34%) | 17 | (8.7, 26) | <0.001 |  | 361 (2.6%) | 1,496 (11%) | 8.3 | 3.3–13 | 0.001 |
| BCC coverage through CNS | 61 (7.8%) | 212 (27%) | 19 | (11, 28) | <0.001 |  | 214 (1.6%) | 1,381 (11%) | 8.2 | 3.3–13 | 0.001 |
| SQ-LNS coverage | 11 (1.4%) | 367 (47%) | 46 | (37, 54) | <0.001 |  | 39 (0.28%) | 4,863 (37%) | 37 | 31–43 | <0.001 |
| SQ-LNS coverage through CNS | 10 (1.3%) | 362 (47%) | 45 | (37, 54) | <0.001 |  | 8 (0.06%) | 4,835 (37%) | 37 | 31–43 | <0.001 |

Data are n (%) unless specified otherwise.

* Statistically significant when considering the critical p-value calculated using the Benjamini-Hochberg method to account for multiple testing of primary outcomes (*P*_critical_= 0.016). ICC for primary outcomes are presented in supplemental table S10

^a^ Difference between intervention and comparison group in percentage point analyzed using a mixed-effects linear probability regression mode with robust estimation of standard errors, with health center catchment area as random effect and distance to health center as fixed effect

^b^ Difference between intervention and comparison group in percentage point analyzed using a mixed-effects regression model with robust estimation of standard errors, with restricted cubic spline, with 7 Knots automatically generated. Models were adjusted for health center catchment area and child as random effects and month of inclusion, age splines distance to health center, relative wealth status and intervention as fixed effects.

^c^ Number of study children

^d^ Number of child-visits

Abbreviations: AM, acute malnutrition; BCC, behavior change communication; CNS, well-baby consultation; ICC, intracluster correlation coefficient; pp, percentage points; SQ-LNS, small quantity lipid-based nutrient supplement
