# Supplementary material for: Impact on child acute malnutrition of integrating a preventive nutrition package into facility-based screening for acute malnutrition during well-baby consultation: A cluster-randomized controlled trial in Burkina Faso
Source: PLoS Med. 2019 Aug 27;16(8):e1002877. doi: 10.1371/journal.pmed.1002877 (PMC6711504; doi:10.1371/journal.pmed.1002877)
Supplement: S5 Table — AM, acute malnutrition. (DOCX) [file pmed.1002877.s006.docx]

**S5 Table: Effect of the intervention on acute malnutrition outcomes assessed by cross-sectional study (robustness analysis adjusting further for distance to health center)**

|  | **Baseline** | |  | **Endline** | | **∆** | **95% CI** | ***P*-value** |
| --- | --- | --- | --- | --- | --- | --- | --- | --- |
|  | **Comparison** | **Intervention** |  | **Comparison** | **Intervention** |  |  |  |
|  | *n* = 1,153 | *n* = 1,160 |  | *n* = 1,165 | *n* = 1,151 |  |  |  |
| AM prevalence (primary outcome) | 141 (12%) | 191 (16%) |  | 149 (13%) | 147 (13%) | -0.32^a^ | -4.3 to 3.6 | 0.87* |
| MAM prevalence | 117 (10%) | 160 (14%) |  | 124 (11%) | 124 (11%) | 0.50 ^a^ | -3.2 to 4.2 | 0.79 |
| SAM prevalence | 24 (2.1%) | 31 (2.7%) |  | 25 (2.2%) | 23 (2.0%) | -0.34 ^a^ | -1.4 to 0.77 | 0.55 |
| Weight-for-length Z-score | -0.58 ± 1.1 | -0.76 ± 1.2 |  | -0.57 ± 1.1 | -0.65 ± 1.1 | -0.010^b^ | -0.12 to 0.090 | 0.82 |
|  | *n* = 746 | *n* = 759 |  | *n* = 770 | *n* = 764 |  |  |  |
| MUAC ^c^, mm | 138 ± 11 | 135 ± 11 |  | 136 ± 10 | 136 ± 10 | -0.14^b^ | -1.9 to 1.6 | 0.88 |

Data are n (%) or mean ± SD.

* Not statistically significant when considering the critical p-value calculated using the Benjamini-Hochberg method to account for multiple testing of primary outcomes (*P*_critical_= 0.016). ICC for primary outcomes are presented in supplemental table S10

^a^ Difference between intervention and comparison group expressed in percentage point analyzed using a mixed-effect linear probability model with robust estimation of standard errors, with health center as random effect and child sex, child age, whether the child was a first live birth, intervention, distance to health center and the cluster means of the outcome at baseline as fixed effects

^b^ Difference between intervention and comparison analyzed using a linear mixed model with health center as random effect and child sex, child age, whether the child was a first live birth, intervention, distance to health center and the cluster means of the outcome at baseline as fixed effects

^c^ MUAC, mid-upper arm circumference, measured in the subsample of children ≥6 months old

Abbreviations: AM, acute malnutrition; ICC, intracluster correlation coefficient; MAM, moderate acute malnutrition; SAM, severe acute malnutrition
