# Supplementary material for: Impact on child acute malnutrition of integrating a preventive nutrition package into facility-based screening for acute malnutrition during well-baby consultation: A cluster-randomized controlled trial in Burkina Faso
Source: PLoS Med. 2019 Aug 27;16(8):e1002877. doi: 10.1371/journal.pmed.1002877 (PMC6711504; doi:10.1371/journal.pmed.1002877)
Supplement: S6 Table — AM, acute malnutrition. (DOCX) [file pmed.1002877.s007.docx]

**S6 Table: Effect of the intervention on the incidence, relapse and longitudinal prevalence of moderate and severe acute malnutrition, longitudinal study**

|  | Comparison | Intervention | IRR/RR | 95% CI | *P*-value |
| --- | --- | --- | --- | --- | --- |
| **First episode of MAM^a^** |  |  |  |  |  |
| *n* of children | 1,081 | 1,032 |  |  |  |
| *n* of first episodes / time at risk^b^, child-years | 627/1,089 | 587/1,046 |  |  |  |
| Incidence | 0.58 | 0.56 | 0.96 ^c^ | 0.70–1.3 | 0.81 |
| **All episodes of MAM** |  |  |  |  |  |
| *n* of children | 1,081 | 1,032 |  |  |  |
| *n* of all episodes / time at risk^d^, child-years | 1,143/1,443 | 1,021/1,386 |  |  |  |
| Incidence | 0.79 | 0.74 | 0.88 ^c^ | 0.66–1.2 | 0.37 |
| **Relapse rate of MAM** |  |  |  |  |  |
| *n* of children | 589 | 556 |  |  |  |
| *n* of relapse episodes / time at risk^e^, child-years | 516/355 | 433/341 |  |  |  |
| Relapse incidence | 1.45 | 1.27 | 0.87 ^c^ | 0.67–1.1 | 0.30 |
|  |  |  |  |  |  |
| **Longitudinal prevalence MAM** |  |  |  |  |  |
| *n* of children | 1,081 | 1,032 |  |  |  |
| Time being MAM / follow-up time, child-years | 133/1,577 | 118/1,505 |  |  |  |
| Prevalence | 8.4 | 7.8 | 0.90 ^f^ | 0.75–1.1 | 0.30 |
|  |  |  |  |  |  |
| **First episode of SAM^g^** |  |  |  |  |  |
| *n* of children | 1,081 | 1,032 |  |  |  |
| *n* of first episodes / time at risk^h^, child-years | 299/1,373 | 274/1,303 |  |  |  |
| Incidence | 0.22 | 0.21 | 0.97 ^c^ | 0.72–1.3 | 0.83 |
| **All episodes of SAM** |  |  |  |  |  |
| *n* of children | 1,081 | 1,032 |  |  |  |
| *n* of all episodes / time at risk^i^, child-years | 370/1,525 | 329/1,458 |  |  |  |
| Incidence | 0.24 | 0.23 | 0.93 ^c^ | 0.69–1.3 | 0.64 |
| **Relapse rate of SAM** |  |  |  |  |  |
| *n* of children | 266 | 242 |  |  |  |
| *n* of relapse episodes / time at risk^j^, child-years | 70/152 | 55/155 |  |  |  |
| Relapse incidence | 0.46 | 0.35 | 0.77 ^c^ | 0.32–1.9 | 0.55 |
| **Longitudinal prevalence SAM** |  |  |  |  |  |
| *n* of children | 1,081 | 1,032 |  |  |  |
| Time being SAM / follow-up time, child-years | 53/1,577 | 48/1,505 |  |  |  |
| Prevalence | 3.3 | 3.2 | 0.93 ^f^ | 0.72–1.2 | 0.61 |

^a^ MAM defined by -3≤weight-for-length Z-score<-2 (all ages) or 115 mm ≤mid-upper arm circumference <125 mm and ( ≥6 mo old), and absence of bilateral pitting edema (all ages) ; Children who suffered from SAM and qualified as MAM during the recovery process were not included in the MAM incidence estimation

^b^ time at risk included all consecutive days before the first episode of MAM

^c^ Incidence rate ratio (IRR) analyzed using a mixed effects Poisson regression model with health center as random effect and child sex, whether the child was a first live birth, month of inclusion and intervention as fixed effects

^d^ time at risk included all consecutive days before, between and after episodes of MAM

^e^ time at risk included all consecutive days before, between and after episodes of MAM, starting after a first episode of MAM

^f^ Risk ratio (RR) analyzed using a mixed-effects Poisson regression model with health center as random effect and child sex, whether the child was a first live birth, month of inclusion and intervention as fixed effects

^g^ SAM defined by weight-for-length<-3 (all ages), mid-upper arm circumference<115 mm (≥6 mo old) or presence of bilateral pitting edema (all ages)

^h^ time at risk included all consecutive days before the first episode of SAM

^i^ time at risk included all consecutive days before, between and after episodes of SAM

^j^ time at risk included all consecutive days before, between and after episodes of SAM, starting after a first episode of SAM

Abbreviations: IRR, incidence rate ratio; MAM, moderate acute malnutrition; RR, risk ratio; SAM, severe acute malnutrition
