# Supplementary material for: Impact on child acute malnutrition of integrating a preventive nutrition package into facility-based screening for acute malnutrition during well-baby consultation: A cluster-randomized controlled trial in Burkina Faso
Source: PLoS Med. 2019 Aug 27;16(8):e1002877. doi: 10.1371/journal.pmed.1002877 (PMC6711504; doi:10.1371/journal.pmed.1002877)
Supplement: S7 Table — AM, acute malnutrition; CMAM, community management of acute malnutrition. (DOCX) [file pmed.1002877.s008.docx]

**S7 Table: Effect of the intervention on CMAM enrollment, treatment and recovery outcomes for acute malnutrition episodes in the longitudinal study (robustness analysis adjusting further for distance to health center and relative wealth status)**

|  | **Comparison** | **Intervention** | **Δ ^a^ (pp)** | **95% CI** | ***P*-value** |
| --- | --- | --- | --- | --- | --- |
| **AM episodes** | n = 1,401 | *n* = 1,275 |  |  |  |
| Enrolled in CMAM | 424 (30%) | 398 (31%) | 0.29 | -6.3 to 6.9 | 0.93 |
|  | *n* = 424 | *n* = 398 |  |  |  |
| Treatment coverage (primary outcome) ^b^ | 93 (22%) | 118 (30%) | 8.1 | -0.61 to 17 | 0.068* |
| Treatment initiated ^c^ | 239 (56%) | 276 (69%) | 12 | 0.73–24 | 0.037 |
| Recovery within 3 months after enrollment | 350 (83%) | 328 (82%) | -0.23 ^d^ | -6.6 to 6.2 | 0.94 |
| Length of enrolled episodes, d | 73 ± 61 | 67 ± 51 | -5.9 ^e^ | -15 to 3.3 | 0.21 |
|  |  |  |  |  |  |
| **MAM episodes** | *n* = 1,143 | *n* = 1,021 |  |  |  |
| Enrolled in CMAM | 338 (30%) | 318 (31%) | 0.65 | -6.3 to 7.5 | 0.85 |
|  | *n* = 338 | *n* = 318 |  |  |  |
| MAM treatment coverage ^b^ | 40 (12%) | 61 (19%) | 7.4 | 0.53–14 | 0.035 |
| MAM treatment initiated ^c^ | 123 (37%) | 155 (49%) | 12 | 0.54–24 | 0.040 |
| Recovery within 3 months after enrollment | 301 (89%) | 280 (88%) | -0.44^d^ | -6.7 to 5.8 | 0.89 |
| Length of enrolled episodes, d | 57 ± 48 | 56 ± 40 | -0.66 ^e^ | -9.8 to 8.5 | 0.88 |
|  |  |  |  |  |  |
| **SAM episodes** | *n* = 370 | *n* = 329 |  |  |  |
| Enrolled in CMAM | 131 (35%) | 119 (36%) | 1.5 | -11 to 14 | 0.82 |
|  | *n* = 131 | *n* = 119 |  |  |  |
| SAM treatment coverage ^b^ | 15 (11%) | 14 (12%) | 2.7 | -6.4 to 12 | 0.56 |
| SAM treatment initiated ^c^ | 47 (36%) | 51 (43%) | 7.2 ^d^ | -7.8 to 22 | 0.94 |
| Recovery within 3 months after enrollment | 99 (76%) | 88 (74%) | -1.0 | -15 to 13 | 0.88 |
| Length of enrolled episodes, d | 75 ± 60 | 75 ± 51 | -1.4 ^e^ | -20 to 17 | 0.88 |

Data are n(%) or mean ± SD.

* Not statistically significant when considering the critical p-value calculated using the Benjamini-Hochberg method to account for multiple testing of primary outcomes (*P*_critical_= 0.016). ICC for primary outcomes are presented in supplemental table S10

^a^ Difference between intervention and comparison group expressed in percentage point analyzed using a mixed-effect linear probability regression model with robust estimation of standard errors, with health center and child as random effects and child sex, child age at the start of the episode, whether the child was a first live birth, month of inclusion, distance to health center, and relative wealth status as fixed effect, unless specified otherwise

^b^ Treatment coverage defined as the proportion of children with AM, MAM or SAM that received continuous treatment from CMAM enrollment onwards over the total number of children with AM, MAM or SAM respectively enrolled in CMAM.

^c^ Treatment initiated implies that children with AM received either a MAM or SAM treatment, children with MAM received MAM treatment and children with SAM received SAM treatment

^d^ child random effect was removed from the model to solve convergence issues

^e^ Difference in mean episode length (days) between intervention and comparison group analyzed using a linear mixed-effects regression model with health center as random effect and child sex, child age at the start of the episode, whether the child was a first live birth and month of inclusion as fixed effect. Child random effect was removed from the model to solve convergence issues

Abbreviations: AM, acute malnutrition; CMAM, community-based management of acute malnutrition; ICC, intracluster correlation coefficient; MAM, moderate acute malnutrition; pp, percentage points; SAM, severe acute malnutrition
