# Supplementary material for: Impact on child acute malnutrition of integrating a preventive nutrition package into facility-based screening for acute malnutrition during well-baby consultation: A cluster-randomized controlled trial in Burkina Faso
Source: PLoS Med. 2019 Aug 27;16(8):e1002877. doi: 10.1371/journal.pmed.1002877 (PMC6711504; doi:10.1371/journal.pmed.1002877)
Supplement: S8 Table — AM, acute malnutrition. (DOCX) [file pmed.1002877.s009.docx]

**S8 Table: Effect of the** **intervention on the incidence, relapse and longitudinal prevalence of acute malnutrition assessed by longitudinal study (robustness analysis adjusting further for distance to health center and relative wealth status)**

|  | Comparison | Intervention | IRR/RR | 95% CI | *P*-value |
| --- | --- | --- | --- | --- | --- |
| **First episode of AM^a^** |  |  |  |  |  |
| *n* of children | 1,081 | 1,032 |  |  |  |
| *n* of first episodes/time at risk^b^, child-years | 719/1,001 | 675/948 |  |  |  |
| Incidence (primary outcome) | 0.72 | 0.71 | 1.0^c^ | 0.73–1.4 | 0.98* |
| **All episodes of AM** |  |  |  |  |  |
| *n* of children | 1,081 | 1,032 |  |  |  |
| *n* of all episodes / time at risk^d^, child-years | 1,401/1,389 | 1,275/1,338 |  |  |  |
| Incidence | 1.0 | 0.95 | 0.40^c^ | 0.73–1.2 | 0.65 |
| **Relapse episodes of AM** |  |  |  |  |  |
| *n* of children | 675 | 639 |  |  |  |
| *n* of relapse episodes/time at risk^e^, child-years | 682/389 | 600/390 |  |  |  |
| Relapse incidence | 1.8 | 1.5 | 0.90^c^ | 0.69–1.2 | 0.42 |
| **Longitudinal prevalence AM** |  |  |  |  |  |
| *n* of children | 1,081 | 1,032 |  |  |  |
| Time being AM / follow-up time, child-years | 187/1,577 | 167/1,505 |  |  |  |
| Prevalence | 12 | 11 | 0.94^f^ | 0.79–1.1 | 0.51 |

* Not statistically significant when considering the critical p-value calculated using the Benjamini-Hochberg method to account for multiple testing of primary outcomes (*P*_critical_= 0.016). ICC for primary outcomes are presented in supplemental table S10

^a^ AM defined by weight-for-length Z-score<-2 (all ages), mid-upper arm circumference <125 mm (≥6 mo old) or presence of bilateral pitting edema (all ages)

^b^ time at risk included all consecutive days before the first episode of AM

^c^ Incidence rate ratio (IRR) analyzed using a mixed effects Poisson regression model with health center as random effect and child sex, whether the child was a first live birth, month of inclusion, intervention, distance to health center, and relative wealth status as fixed effects

^d^ time at risk included all consecutive days before, between and after episodes of AM

^e^ time at risk included all consecutive days before, between and after episodes of AM, starting after a first episode of AM

^f^ Risk ratio (RR) analyzed using a mixed-effects Poisson regression model with health center as random effect and child sex, whether the child was a first live birth, month of inclusion, intervention, distance to health center, and relative wealth status as fixed effects

Abbreviations: AM, acute malnutrition; ICC, intracluster correlation coefficient; IRR, incidence rate ratio; RR, risk ratio
