# Supplementary material for: Impact on child acute malnutrition of integrating a preventive nutrition package into facility-based screening for acute malnutrition during well-baby consultation: A cluster-randomized controlled trial in Burkina Faso
Source: PLoS Med. 2019 Aug 27;16(8):e1002877. doi: 10.1371/journal.pmed.1002877 (PMC6711504; doi:10.1371/journal.pmed.1002877)
Supplement: S9 Table — CNS, well-baby consultation (consultation du nourrisson sain). (DOCX) [file pmed.1002877.s010.docx]

**S9 Table: Reasons for not attending CNS in the past month, by age range and study group**

|  |  | children 0-5 months old | | | |  | children 6-18 months old | | | |
| --- | --- | --- | --- | --- | --- | --- | --- | --- | --- | --- |
|  |  | Comparison *n* = 239 | | Intervention *n* = 261 | |  | Comparison *n* = 596 | | Intervention *n* = 322 | |
|  | *n* | % | rank | % | rank |  | % | rank | % | rank |
| **Not necessary yet** | 455 | **116 (49%)** | **1** | **146 (56%)** | **1** |  | **132 (22%)** | **1** | **61 (19%)** | **2** |
| **Lack of time** | 211 | 10 (4.2%) | 7 | 12 (4.6%) | 5 |  | **105 (18%)** | **2** | **84 (26%)** | **1** |
| **No appointment** | 169 | **35 (15%)** | **2** | **46 (18%)** | **2** |  | 53 (8.9%) | 4 | **35 (11%)** | **3** |
| **CNS has not taken place** | 114 | 23 (9.6%) | 3 | 18 (6.9%) | 4 |  | 38 (6.4%) | 9 | **35 (11%)** | **4** |
| No vaccination needed | 110 | 15 (6.3%) | 5 | 22 (8.4%) | 3 |  | 52 (8.7%) | 6 | 21 (6.5%) | 7 |
| **Not informed** | 99 | 18 (7.5%) | 4 | 6 (2.3%) | 7 |  | **66 (11%)** | **3** | 9 (2.8%) | 9 |
| Other reasons | 95 | 7 (2.9%) | 9 | 9 (3.4%) | 6 |  | 48 (8.1%) | 8 | 31 (9.6%) | 5 |
| Too far | 93 | 13 (5.4%) | 6 | 4 (1.5%) | 8 |  | 52 (8.7%) | 5 | 24 (7.5%) | 6 |
| Don't know | 75 | 4 (1.7%) | 11 | 2 (0.77%) | 11 |  | 50 (8.4%) | 7 | 19 (5.9%) | 8 |
| Not well received | 47 | 9 (3.8%) | 8 | 2 (0.77%) | 10 |  | 27 (4.5%) | 11 | 9 (2.8%) | 10 |
| Does not see the need | 40 | 1 (0.4%) | 14 | 4 (1.5%) | 9 |  | 29 (4.9%) | 10 | 6 (1.9%) | 11 |
| Child not malnourished | 22 | 3 (1.3%) | 12 | 1 (0.38%) | 12 |  | 14 (2.3%) | 12 | 4 (1.2%) | 12 |
| No resources | 15 | 3 (1.3%) | 13 | 0 (0.0%) | 14 |  | 8 (1.3%) | 13 | 4 (1.2%) | 13 |
| Not allowed | 7 | 4 (1.7%) | 10 | 0 (0.0%) | 13 |  | 2 (0.3%) | 15 | 1 (0.3%) | 15 |
| Product shortage | 4 | 0 (0.0%) | 15 | 0 (0.0%) | 15 |  | 2 (0.3%) | 14 | 2 (0.6%) | 14 |

Data are n(%) or ranks. Caregivers could report multiple reasons for not attending. Reasons for not attending CNS which were cited by more than 10% of caregivers in an age range and study group are in bold

Abbreviations: CNS, well-baby consultation
