# Supplementary figures and images for: Impact on child acute malnutrition of integrating a preventive nutrition package into facility-based screening for acute malnutrition during well-baby consultation: A cluster-randomized controlled trial in Burkina Faso
Source: PLoS Med. 2019 Aug 27;16(8):e1002877. doi: 10.1371/journal.pmed.1002877 (PMC6711504; doi:10.1371/journal.pmed.1002877)

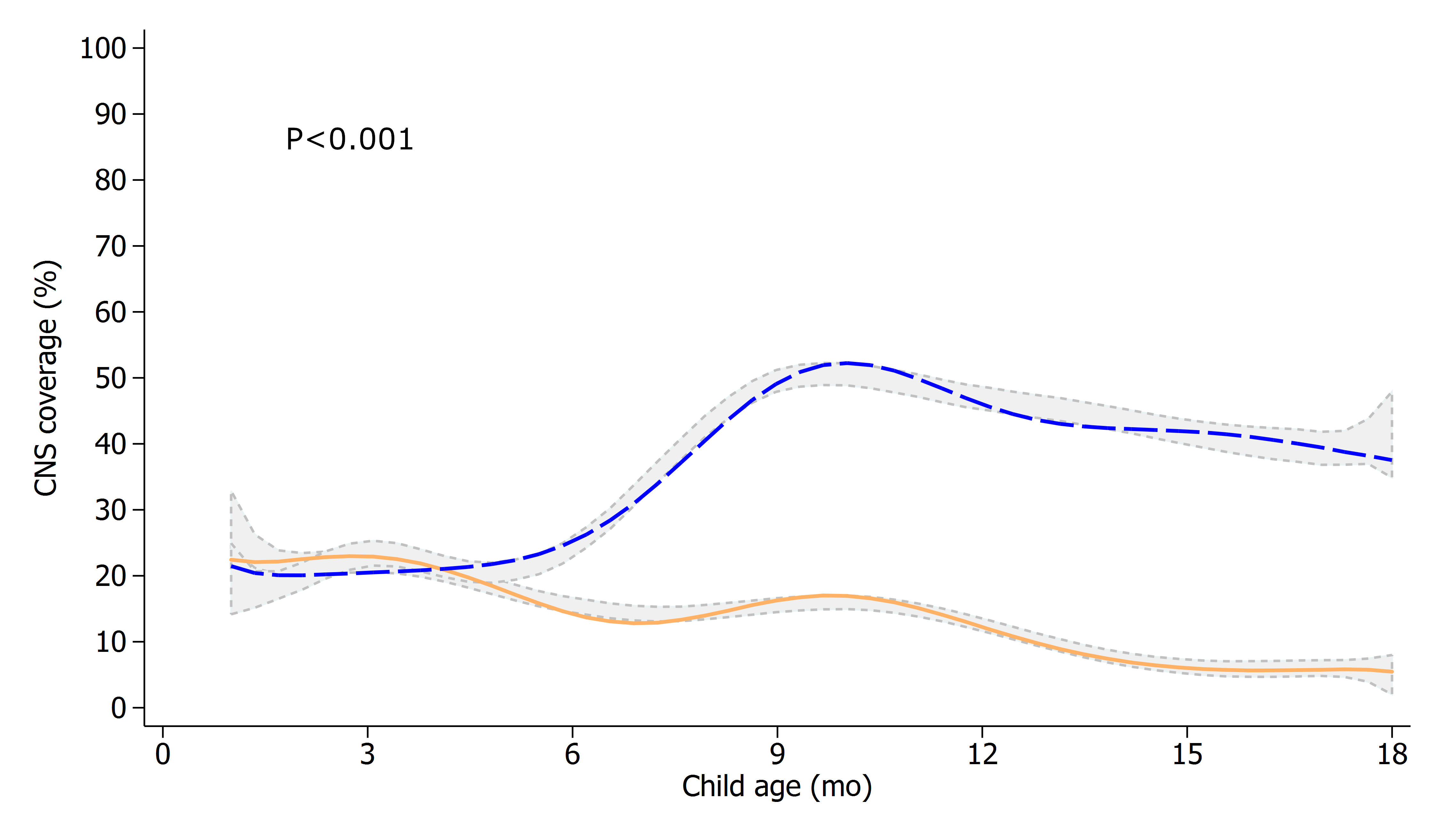

Supplement: S1 Fig — Gray areas represent 95% confidence bands of kernel-weighted local polynomial smoothed values by study group using the observed data. The orange solid line represents fitted values for the comparison group. The blue dashed line represents fitted values for the intervention group. Analysis was based on n = 18,757 child visits in comparison group and n = 17,867 child visits in intervention group. Mixed-effects regression model with restricted cubic splines (7 knots automatically generated) was used, with health center catchment area and child as random intercepts and month of inclusion, child sex, age splines, whether the child was a first live birth, and intervention as fixed effects. A chunk Wald test was used to test the “age splines × intervention” interaction terms (P value shown). CNS, well-baby consultation (consultation du nourrisson sain). (TIF) [file pmed.1002877.s011.tif]

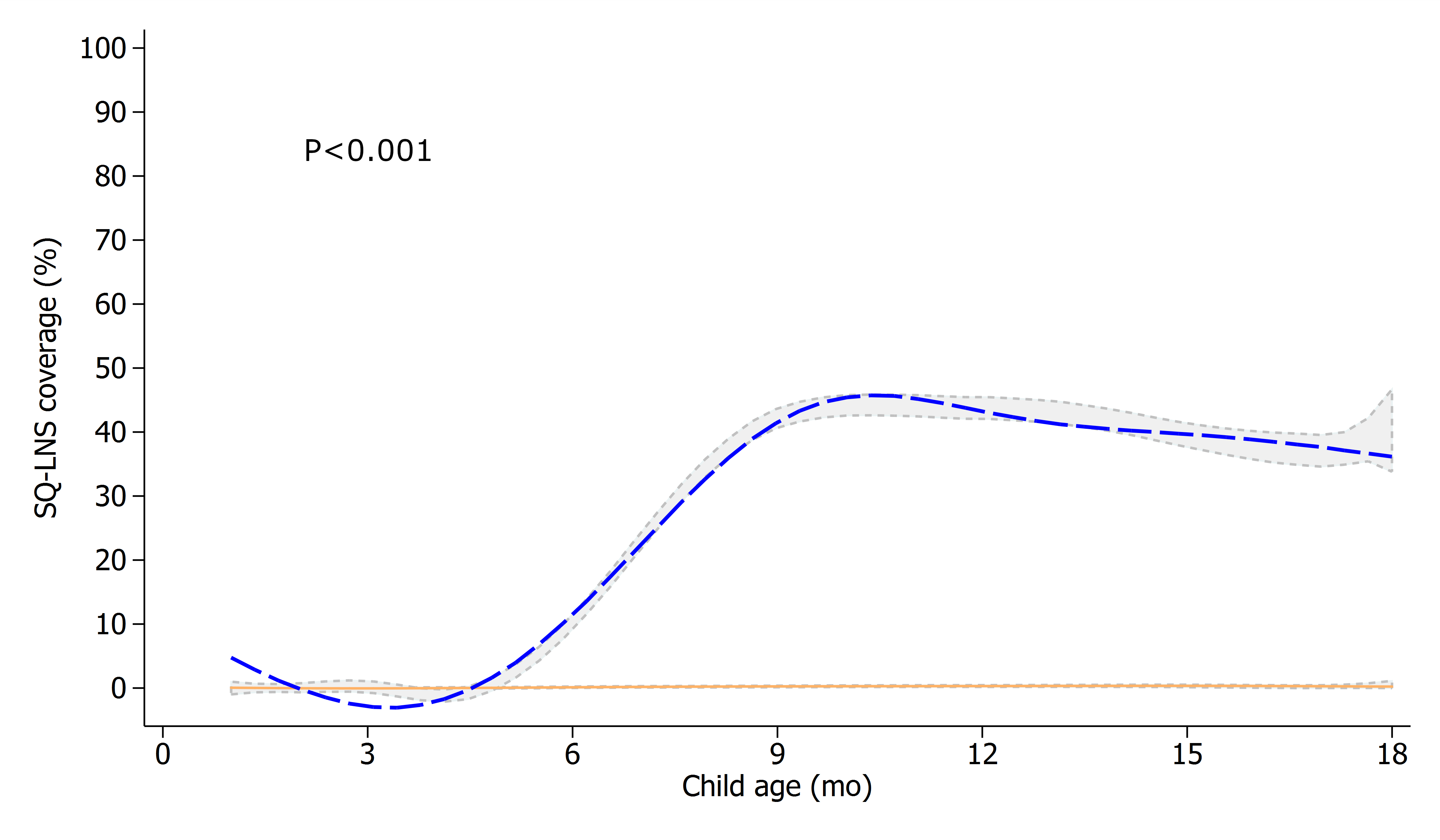

Supplement: S2 Fig — The orange solid line represents fitted values for the comparison group. The blue dashed line represents fitted values for the intervention group. Gray areas represent 95% confidence bands of kernel-weighted local polynomial smoothed values by study group using the observed data. Analysis was based on n = 18,757 child visits in comparison group and n = 17,867 child visits in intervention group. Mixed-effects regression model with restricted cubic splines (7 knots automatically generated) was used, with health center catchment area and child as random intercepts and month of inclusion, child sex, age splines, whether the child was a first live birth, and intervention as fixed effects. A chunk Wald test was used to test the “age splines × intervention” interaction terms (P value shown). SQ-LNS, small-quantity lipid-based nutrition supplement. (TIF) [file pmed.1002877.s012.tif]

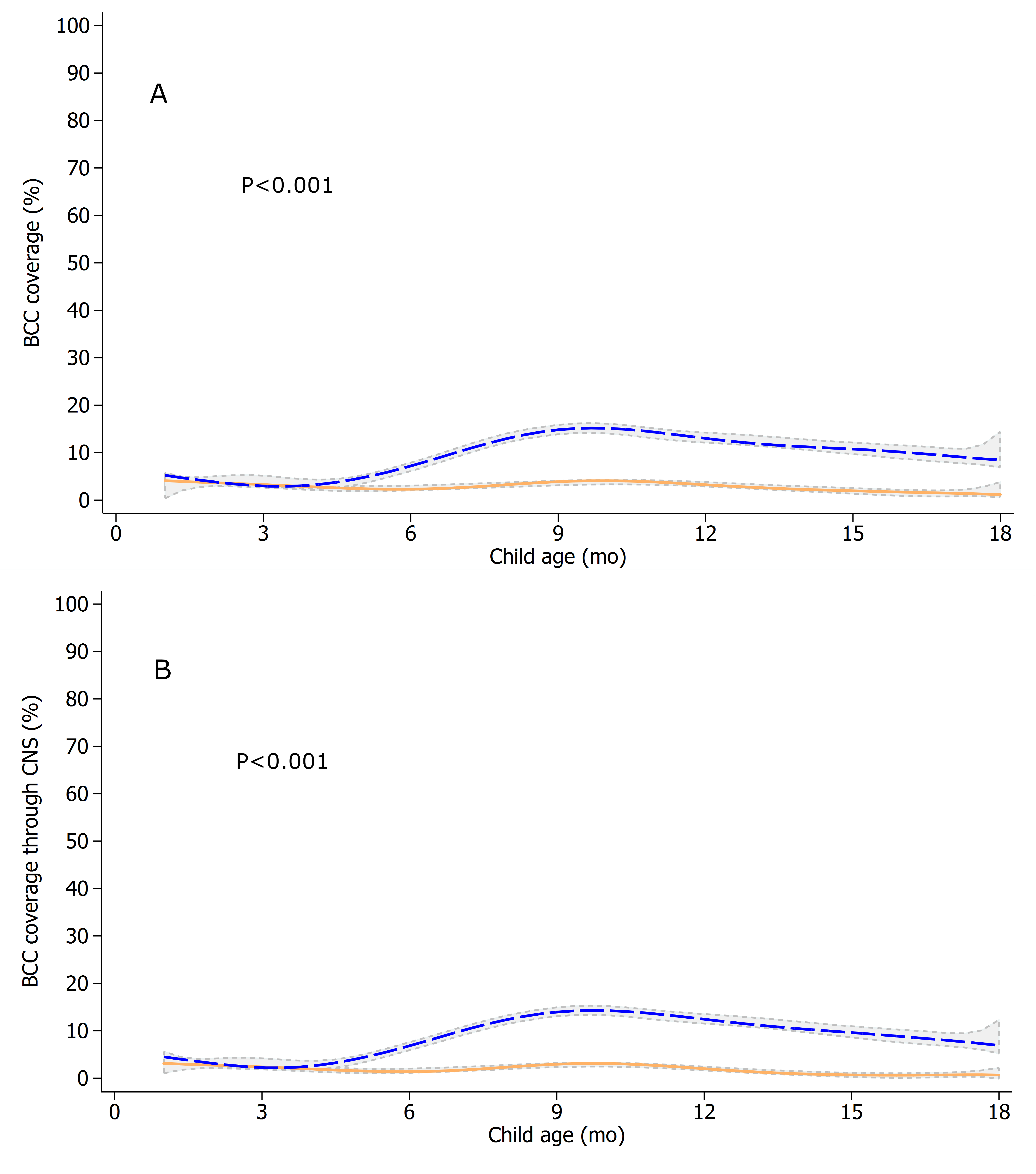

Supplement: S3 Fig — The orange solid line represents fitted values for the comparison group. The blue dashed line represents fitted values for the intervention group. Gray areas represent 95% confidence bands of kernel-weighted local polynomial smoothed values by study group using the observed data. Both analyses were based on n = 18,757 child visits in comparison group and n = 17,867 child visits in intervention group. Mixed-effects regression models with restricted cubic splines (7 knots automatically generated) were used, with health center catchment area and child as random intercepts and month of inclusion, child sex, age splines, whether the child was a first live birth, and intervention as fixed effects. A chunk Wald test was used to test the “age splines × intervention” interaction terms (P values shown). BCC, behavior change communication; CNS, well-baby consultation (consultation du nourrisson sain) (TIF) [file pmed.1002877.s013.tif]

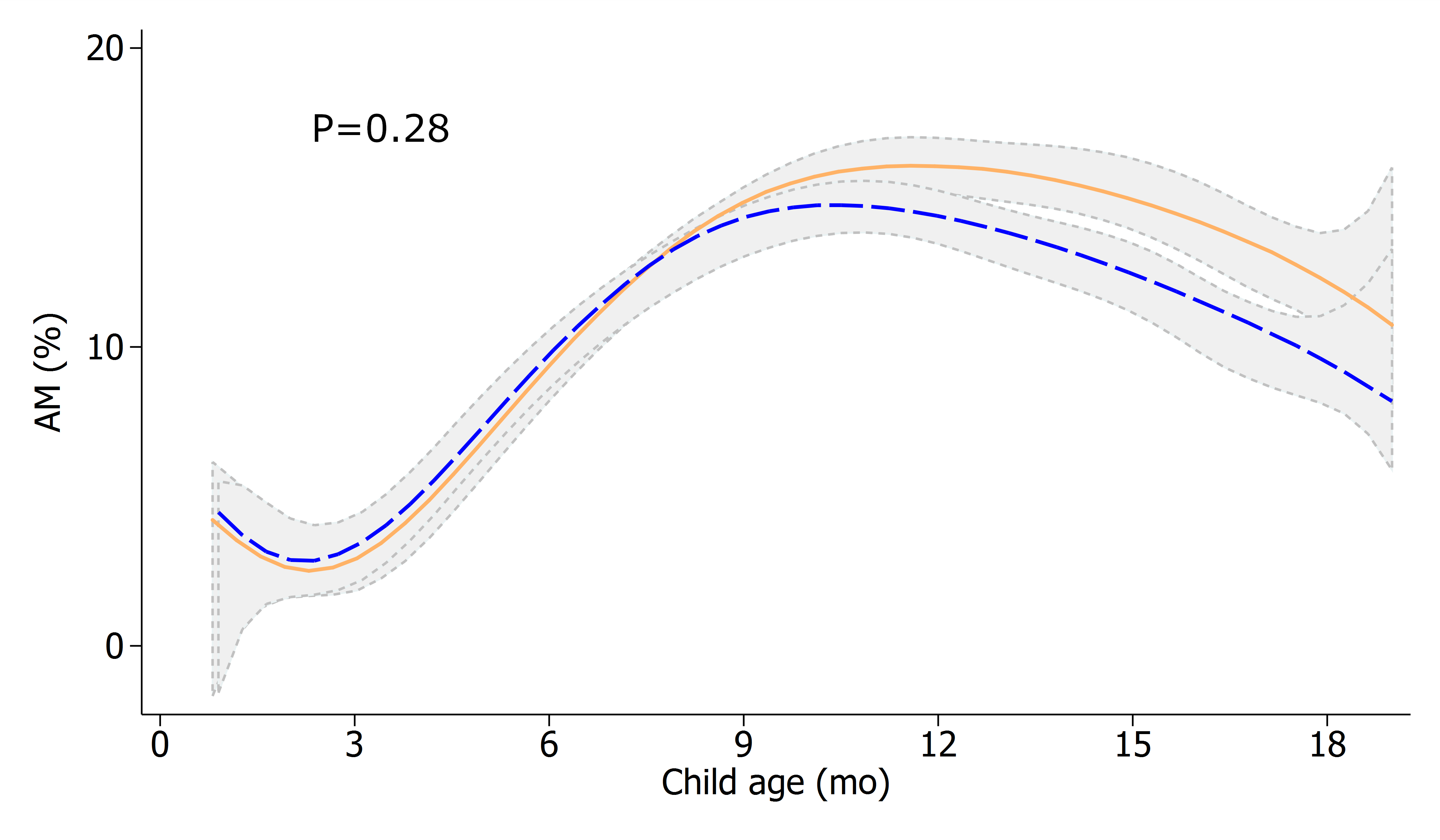

Supplement: S4 Fig — AM is defined by a weight-for-length z-score < −2 (all ages) or mid-upper arm circumference < 125 mm (≥6 months old) or presence of bilateral pitting edema (all ages). The orange solid line represents fitted values for the comparison group. The blue dashed line represents fitted values for the intervention group. Gray areas represent 95% confidence bands of kernel-weighted local polynomial smoothed values by study group using the observed data. Analysis was based on n = 18,090 child visits in comparison group and n = 17,266 child visits in intervention group. Mixed-effects regression model with robust estimation of standard errors and with restricted cubic splines (knots at 3 and 6 months) was used, with health center catchment area and child as random intercepts and month of inclusion and child sex, age splines, whether the child was a first live birth, and intervention as fixed effects. A chunk Wald test was used to test the “age splines × intervention” interaction terms (P value shown). AM, acute malnutrition. (TIF) [file pmed.1002877.s014.tif]

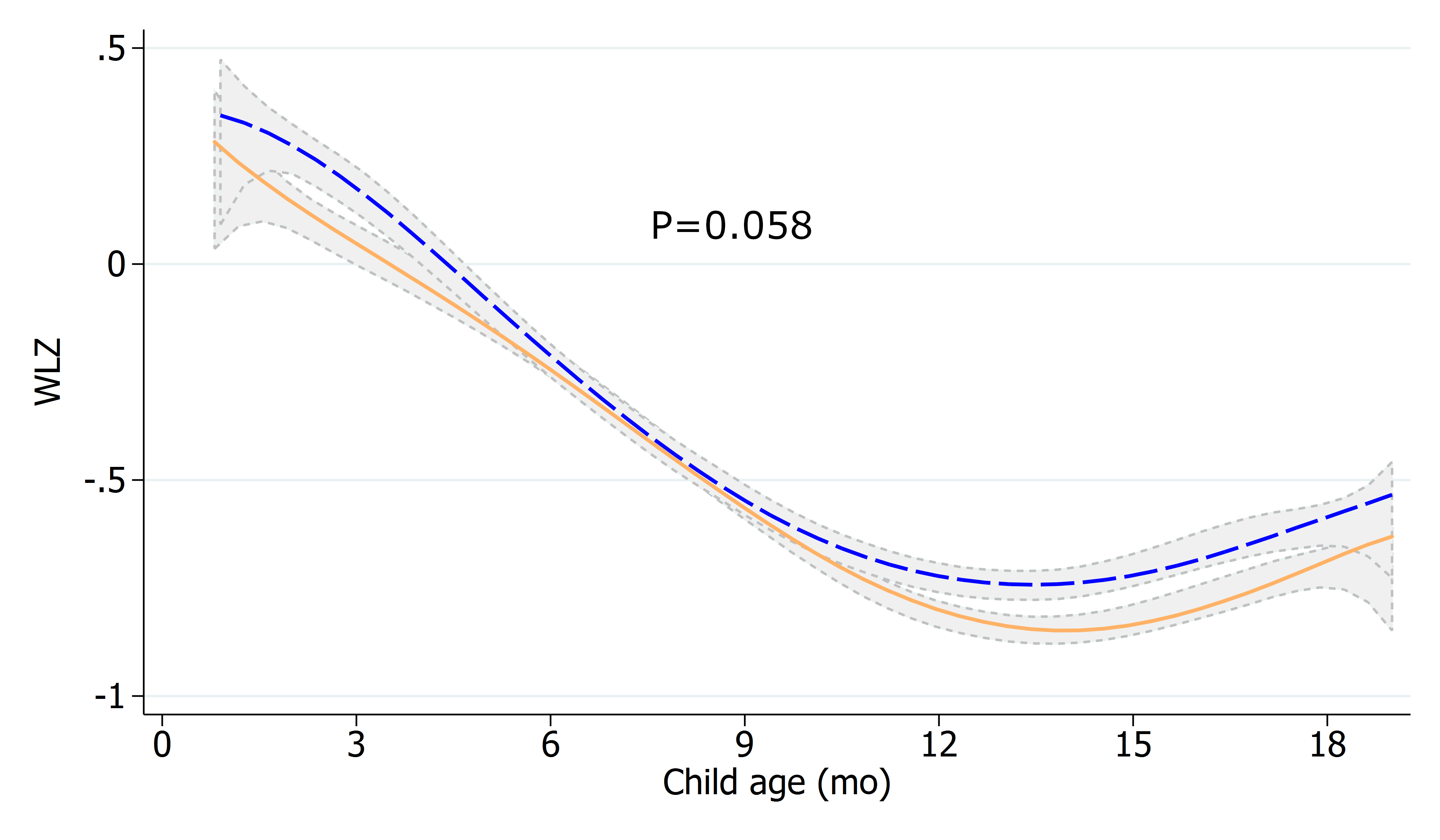

Supplement: S5 Fig — The orange solid line represents fitted values for the comparison group. The blue dashed line represents fitted values for the intervention group. Gray areas represent 95% confidence bands of kernel-weighted local polynomial smoothed values by study group using the observed data. Analysis was based on n = 18,090 child visits in comparison group and n = 17,266 child visits in intervention group. Mixed-effects regression model with restricted cubic splines (knots at 3, 6, and 12 months) was used, with health center catchment area and child as random intercepts and month of inclusion, child sex, age splines, whether the child was a first live birth, and intervention as fixed effects. A chunk Wald test was used to test the “age splines × intervention” interaction terms (P value shown). WLZ, weight-for-length z-score. (TIF) [file pmed.1002877.s015.tif]

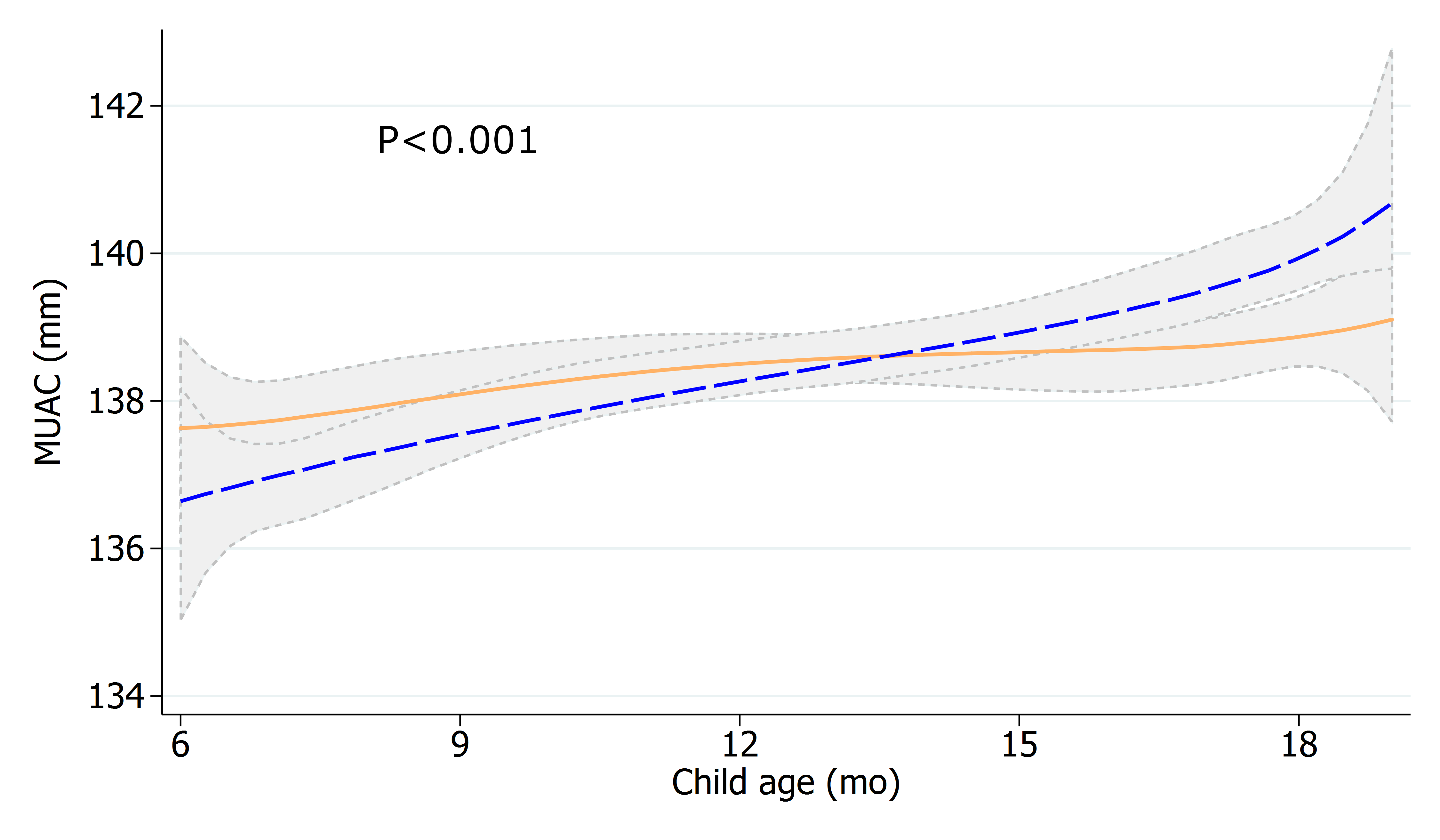

Supplement: S6 Fig — The orange solid line represents fitted values for the comparison group. The blue dashed line represents fitted values for the intervention group. Gray areas represent 95% confidence bands of kernel-weighted local polynomial smoothed values by study group using the observed data. Analysis was based on n = 13,073 child visits in comparison group and n = 12,485 child visits in intervention group. Linear mixed-effects regression model was used, with health center catchment area and child as random intercepts and month of inclusion, child sex, age splines, whether the child was a first live birth, and intervention as fixed effects. We tested the “age × intervention” interaction term (P value shown). MUAC, mid-upper arm circumference. (TIF) [file pmed.1002877.s016.tif]
