# Supplementary material for: Impact on child acute malnutrition of integrating a preventive nutrition package into facility-based screening for acute malnutrition during well-baby consultation: A cluster-randomized controlled trial in Burkina Faso
Source: PLoS Med. 2019 Aug 27;16(8):e1002877. doi: 10.1371/journal.pmed.1002877 (PMC6711504; doi:10.1371/journal.pmed.1002877)
Supplement: S1 Text — PROMIS, Innovative Approaches for the Prevention of Childhood Undernutrition. (PDF) [file pmed.1002877.s017.pdf]

The PROMIS study

The effect of integrated prevention and treatment on child malnutrition and health in Burkina Faso: a cluster randomized intervention study

IFPRI & Helen Keller International

THE PROMIS PROJECT

SUMMARY SHEET

**Country:**	Burkina Faso
**Type of project:**	Research project<br>Cluster-randomized, non-masked, community-based, effectiveness trial
**Project Title:**	**The effect of integrated prevention and treatment on child malnutrition in Burkina Faso**
**Version:**	Version 1.0, dated 17-July-2014
**Project codes:**	PROMIS-01<br>ClinTrials.Gov: registration to be completed once IRB and EC clearance is obtained
**Project summary:**	The overall objective of this project is to lower the prevalence of acute and chronic malnutrition through an integration of preventive BCC/SQ-LNS and enhanced contact coverage and treatment referral and adherence in the Gourcy health district, Northern region, Burkina Faso, West-Africa.<br>The evaluation will be based on a cluster randomized controlled trial randomizing 34 health center catchment areas and will adopt 2 different evaluation designs: i) a comparison of the control (n=1,020) and intervention (n=1,020) groups through a longitudinal follow-up of 18 months of program participation; ii) an endline comparison between intervention (n=1,190) and control study groups (n=1,190) on primary and secondary outcomes.<br>The main study outcomes are:<br>- Prevalence of child acute malnutrition (wasting) at the end of the program in a sample of 1-17 months old children;<br>- Incidence of child acute malnutrition over 18 months of program intervention;<br>- Coverage of case-finding and treatment of child acute malnutrition over 18 months of program intervention and at the end of the program<br>- Cost-effectiveness of the newly proposed program
**Project Duration:**	30 months
**Starting Date:**	September 2014

**Completion Date:**	February 2017
**Executing Agency:**	Helen Keller International Burkina Faso
**Sponsor :**	International Food Policy Research Institute
**Project Contacts:**	Dr Marie Ruel (PI) – IFPRI – m.ruel@cgiar.org<br>Dr Rahul Rawat (PI) – IFPRI– r.rawat@cgiar.org<br>Dr Jean-Célestin Somda(PI) – HKI - Jsomda@hki.org

STATEMENT OF COMPLIANCE & CONFIDENTIALITY

By signing this protocol, the Principal Investigator and the Sponsor acknowledge and agree:

This protocol contains all necessary information for conducting this study. The Principal Investigators will conduct this study as detailed herein and will make every reasonable effort to complete the study within the time designated. The Principal Investigators commits to carry out the study in compliance with the protocol, amendments, applicable procedures and other study-related documents provided by the Sponsor, and in compliance with applicable ethical and regulatory requirements.

The protocol and all relevant information will be made available to all relevant government institutions of Burkina Faso who participate in conducting this study.

This document may contain information that is privileged or confidential. As such, it may not be disclosed to any other than involved research staff, research partner institutions and the concerned Ethics Committee(s) and IRB, unless specific permission is granted in writing by the International Food Policy Research Institute and Helen Keller International, or such disclosure is required by federal or other laws or regulations. In any event, persons to whom the information is disclosed must be informed that the information is privileged and confidential and may not be further disclosed by them.

Principal Investigator International Food Policy Research Institute

Name: Dr Marie Ruel Date: 18 July 2014

Signed:

Principal Investigator International Food Policy Research Institute

Name: Dr Rahul Rawat Date: 18 July 2014

Signed:

Principal Investigator Helen Keller International

Name: Dr Jean-Célestin Somda Date: 18 July 2014

Signed:

TABLE OF CONTENTS

1 PROBLEM STATEMENT AND STUDY JUSTIFICATION 1

2 OBJECTIVES, RESEARCH QUESTIONS AND HYPOTHESES 3

2.1 OVERALL OBJECTIVE 3
2.2 RESEARCH QUESTIONS 3
2.3 RESEARCH HYPOTHESES 4

3 RESEARCH METHODS 5

3.1 STUDY POPULATION 5
3.2 PROGRAMME DESCRIPTION 6
3.2.1 BACKGROUND ON THE NATIONAL CMAM PROGRAM 6
3.2.2 CMAM ACTIVITIES IMPLEMENTED BY HKI FOR PROMIS IN GOURCY DISTRICT 8
3.3 PROGRAMME IMPACT EVALUATION 11
3.3.1 STUDY DESIGN 11
3.3.2 RANDOMIZATION 12
3.3.3 REPEATED CROSS-SECTIONAL STUDY 12
3.3.4 LONGITUDINAL STUDY 14
3.3.5 MEASUREMENT AND PROCEDURES 16
3.3.6 DATA MANAGEMENT AND ANALYSIS 18

4 ETHICAL APPROVAL 20

5 PARTNERSHIP AND MANAGEMENT 21

6 BUDGET 22

7 TIMELINE 23

8 REFERENCE LIST 23

List of Abbreviations

ABC	Activity-Based Costing
ARI	Acute Respiratory Infections
BCC	Behavior change communication
CHA	Community Health Associations
CHD	Child Health Days
CHV	Community health volunteers
CHW	Community Health Workers
CMAM	Community-based Management of Acute Malnutrition
CNS	Consultation du Nourrisson Sain
CREN	Centre de Réhabilitation et d'Education Nutritionelle
CSPS	Centre de Santé et de Promotion Sociale
DFATD	Department of Foreign Affairs, Trade and Development
DHT	District Health team
DMO	District Medical Officer
EHFP	Enhanced Homestead Food Production
ENA	Essential Nutrition Actions
EPI	Expanded Program on Immunization
FGD	Focus Group Discussion
HAZ	Height-for-Age Z-score
Hb	Hemoglobin
HKI	Helen Keller International
IFPRI	International Food Policy Research Institute
IMCI	Integrated Management of Childhood Illness
IYCF	Infant and Young Child Feeding Indicators
MAM	Moderate Acute Malnutrition
MNP	Multiple micronutrient powder
MOH	Ministry Of Health
MUAC	Mid-Upper Arm Circumference
NGO	Non Governmental Organization
ORS	Oral Rehydration Salts
RUSF	Ready-to-Use Supplementary Food
RUTF	Ready-to-Use Therapeutic Food
SAM	Severe Acute Malnutrition
SQ-LNS	Small Quantity Lipid Based Nutrient Supplement
UNICEF	United Nations Children's Fund
VAS	Vitamin A Supplements
WASH	Water, Sanitation & Hygiene
WAZ	Weight-for-Age Z-Score
WHO	World Health Organization
WHZ	Weight-for-Height Z-Score
WFP	Word Food Programme

1 Problem statement and study justification

Globally, child undernutrition is underlying cause for 3.1 million deaths of children younger than 5 years, representing about 45% of all deaths in this group. Within this context, 18.7 million children under five years of age suffer from severe acute malnutrition (SAM), defined by a Weight-for-age Z-score less than -3 or a MUAC<115 mm. An additional 33 million children under five are at risk of becoming SAM being in a stage of moderate acute malnutrition (defined by a WHZ<-2 or a MUAC <125mm). This situation is aggravated by the presence of child chronic malnutrition expressed by stunted linear growth that affects 163 million children worldwide and important micronutrient deficiencies of which the most problematic are vitamin A, zinc, iron and iodine [1].

The landscape for treatment of child severe acute malnutrition has changed dramatically in the last 10 years with the advent of ready-to-use therapeutic foods (RUTFs) that are easier to manage in field conditions, and the adoption of treatment protocols that promote treatment of cases of SAM without complications in outpatient programmes supported by community-based and primary health care delivery platforms through an approach called Community-based Management of Acute Malnutrition (CMAM).

However, in contrast to an isolated management of SAM, the CMAM model consists of an integrated approach for the management of both SAM and MAM. In 2012, the WHO formulated a set of recommendations for supplementary feeding programs specifically aimed at MAM that are currently being implemented in 89 countries worldwide under the umbrella of CMAM [2]. Access to services for treatment of MAM/SAM has expanded rapidly and has proven to be highly effective for saving the lives of acutely malnourished children.

In Sub-Sahara African countries, like Burkina Faso, which have increased availability of MAM/SAM services, a major challenge remains in ongoing, effective screening and referral coverage to optimize early detection and uptake of these services. A recent survey in neighboring Mali quantified screening coverage of child acute malnutrition by bi-annual mass-screening campaigns (Child Health Days), community health volunteers or health center staff [3]. Whereas participation in Child Health Days was quite high (82% of 1543 eligible children), only 52% of the participating children were screened using the MUAC criterion. Routine screening performed even worse with only 22% and less than 5% of eligible children screened by community health volunteers and health center staff respectively. An even more worrisome finding was that only 57% of the identified cases during the CHD actually visited a health center for a confirmatory diagnosis and the initiation of treatment.

Preventing all forms of child undernutrition requires ensuring optimal infant and young child feeding practices and preventing and treating infectious diseases. Exclusive breastfeeding is recommended for the first 6 months of a child's life, after which complementary foods are needed to complement breast milk and ensure adequate intake of essential nutrients.
In most resource-constrained environments, especially in Sub-Saharan Africa, households lack the means to ensure a nutritionally adequate diet for their 6–23 month old children; hence a more limited impact by BCC interventions only on child undernutrition should be expected. The advent of specialized products, however, such as multiple micronutrient powders (MNPs) and small quantity lipid-based nutrient supplements (SQ-LNS) provides an opportunity to combine behavior change communication (BCC) programs that promote optimal Essential Nutrition Actions (ENA) and Infant and Young Child Feeding (IYCF) practices with access to products that help food-insecure households provide a nutritionally adequate complementary feeding diet to their young children. To date, the evidence on the efficacy of MNP and LNS on child undernutrition is not completely consistent. A recent Cochrane review concluded that MNPs were effective in cutting the risk of iron deficiency in half, but effects on vitamin A and zinc levels were less consistent between studies [4]. Interestingly though, in contrast to MNPs [4–6], embedding the multiple micronutrients in a small quantity of energy dense spread like LNS triggered additional effects on linear growth and the prevention of stunting in several intervention studies[7–9].

There is often poor integration between programs to treat child acute malnutrition and programs focused on the prevention of acute and chronic undernutrition – resulting in many missed opportunities for using prevention platforms to screen and refer SAM children, or for using screening and referral platforms to provide prevention services. Reasons for this discrepancy include the different actors or delivery channels of treatment and prevention of child undernutrition and the different dynamic of these programs or strategies. Whereas the treatment of acute malnutrition evolved from an emergency response and is defined a more limited time frame, preventive BCC interventions might require a longer time frame. In resource-limited settings, a combination of health facility and community-based platforms seems a promising strategy to harmonize both preventive and curative interventions. Employing community-based platforms with lay health workers to promote maternal and child health and development typically results in improved immunization coverage, better breastfeeding practices and reduced child morbidity and mortality compared to usual care [10].

Effective BCC combined with the distribution of specialized products such as RUTF for SAM, RUSF for MAM, and SQ-LNS for the prevention of both acute and chronic undernutrition are key elements of successful programs to tackle the whole spectrum of childhood undernutrition problems – from prevention of chronic

undernutrition to prevention and treatment of MAM/SAM. A variety of program models combining BCC and specialized products have been developed for different contexts, but preventive and treatment programs have generally been implemented in a parallel rather than in an integrated manner.

This project will address two critical gaps related to the integration of preventive and treatment programs: 1) screening and treatment of MAM/SAM have not yet been systematically integrated into routine health-center visits or mainstreamed into community outreach programs; and 2) screening programs do not offer any preventive services for those children found not to be suffering from MAM/SAM at the time of screening; mothers of children identified as non-MAM/SAM case are usually sent home without receiving any health or nutrition inputs and as a result, may fail to come back for screening because they do not see any tangible benefit associated with their participation in the screening. This project will specifically address these gaps by identifying and testing innovative platforms to integrate treatment and prevention interventions for both chronic and acute malnutrition.

2 Objectives, research questions and hypotheses

2.1 Overall objective

The overall study objective is to reduce the prevalence of child malnutrition by integrating preventive and treatment protocols through combined health facility and community based platforms.
This study aims at delivering empirical evidence that the CMAM program implemented by HKI which integrates preventive and treatment schedules related to child undernutrition at different levels (health facility and community based), when integrating also the provision of SQ-LNS and improved BCC through the most frequently used platform for screening, referral and treatment (health facility), is able to improve child nutritional status and IYCF/ENA/WASH knowledge and practices.
In particular, the HKI program integrates i) enhanced case-finding, referral and compliance to treatment; with ii) strong preventive component combining the distribution of a small quantity of LNS and a strengthened nutrition education component. Moreover, a strong focus will be put on the community-based platform of health workers that is already quarterly involved in malnutrition screening and other actors like traditional healers and grandmothers at the community and household level.

2.2 Research Questions

A rigorous program evaluation of HKI's program will be performed by IFPRI. For evaluations purposes, the following research questions will be addressed:

I. Feasibility of integrating preventive services into treatment programs

1) Is provision of SQ-LNS through health facility logistically feasible?
2) Is provision of enhanced IYCF/ENA/WASH BCC through health facility logistically feasible?
3) Does increased referral load because of provision through health facility of SQ-LNS and/or of enhanced IYCF and ENA BCC overload treatment services?
4) Is the SQ-LNS product culturally acceptable and used as recommended for the prevention of child undernutrition?

II. Impacts on coverage, uptake and completion of MAM/SAM screening services

1) Does provision through health facility of enhanced IYCF/ENA/WASH BCC to 0-17 mo old children and of SQ-LNS to 6-18 mo old children increase
    a. MAM/SAM screening coverage?
    b. MAM/SAM outpatient treatment compliance and rate of completion of full treatment?

III. Impacts on incidence and prevalence of acute malnutrition

1) Does provision of enhanced IYCF/ENA/WASH BCC to 0-17 mo old children and of SQ-LNS to 6-17 mo old children reduce:
    a. the incidence of wasting (MAM/SAM)
    b. the prevalence of wasting (through prevention and improved screening/referral/treatment) at study termination?
2) Does provision of enhanced IYCF/ENA/WASH BCC to 0-17 mo old children and of SQ-LNS to 6-17 mo old children reduce:
    a. the incidence of child stunting
    b. the prevalence of stunting at study termination?
3) Does the provision of SQ-LNS and enhanced BCC through health facility result in an improved behavior by the caregiver related child's health and nutrition including improved IYCF practices?
4) What is the cost and cost effectiveness of providing preventive SQ-LNS and enhanced BCC on screening, prevention and treatment of child undernutrition?

2.3 Research hypotheses

We hypothesized that integration of monthly distribution of SQ-LNS and intensified BCC in small groups within health facility, which is the most frequently used malnutrition screening, referral and treatment platform:

1) Reduces the prevalence of acute malnutrition (MAM/SAM) in children 1-17 months of age
2) Reduces the incidence of acute malnutrition (new cases of MAM/SAM) in children 0-17 months of age
3) Reduces the prevalence of chronic malnutrition (stunting) in children 1-17 months of age
4) Reduces the incidence of stunting (new cases) in children 0-17 months of age
5) Increases screening coverage (proportion of children monthly screened / total number of eligible children) for acute malnutrition (MAM/SAM)
6) Increases treatment compliance for MAM cases (number of MAM children adhering to weekly treatment until discharged / number of MAM children starting treatment)
7) Increases blood hemoglobin concentration in children 3-17 months of age
8) Reduces anemia (Hb<11g.dL^{-1}) in children 3-17 months of age
9) Improves IYCF/ENA/WASH knowledge and practices
10) Results in a better cost-effectiveness to reduce the prevalence of acute and chronic malnutrition in children 1-17 months of age.

3 Research Methods

3.1 Study population

Burkina Faso is a low-income, West African country with 14 million inhabitants, 44% of whom live on less than US$1.25/day. The study will be conducted in the Gourcy health district in the North region that includes 28 rural Health Centers (HC) and one urban HC. Population statistics of this district show a total of 46,000 children aged 0-23 months. From the most recent Demographic and Health Survey (2010) a 16.5% prevalence of child acute malnutrition together with a 38.5% prevalence of child chronic malnutrition or stunting was reported for the North region [11]. During the last 5 years, Helen Keller international has supported the implementation of integrated CMAM to tackle the burden of child acute malnutrition by improved screening, referral and treatment of acute malnutrition (eg. Yako district). In the same region, 91% of children between 6 and 59 months of ages suffer from anemia (Hb<11.0g.dL^{-1}) and 13.1% suffers from severe anemia (Hb<7.0 g.dL^{-1}). Malaria is holoendemic in this region with annual transmission peaks during the wet season (July-September).

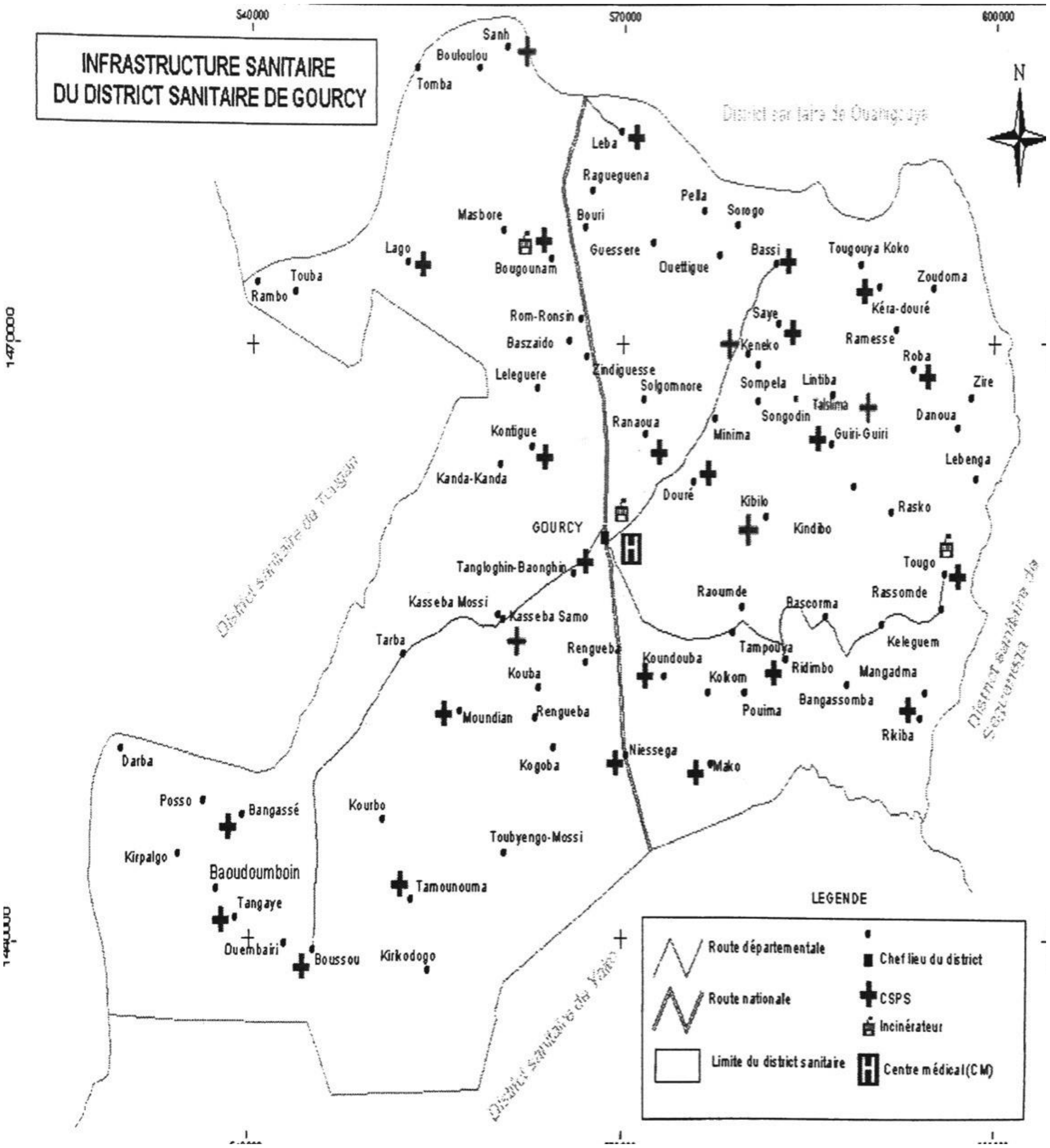

Figure 1 Situation of the Gourcy Health district

3.2 Programme description

3.2.1 Background on the national CMAM program

3.2.1.1 National CMAM strategy

The national CMAM guidelines in Burkina Faso were recently reviewed and updated to conform to international (WHO) standards. Training modules covering CMAM will be reviewed in the near future. Routine CMAM services (management

of MAM and SAM) are available at health facilities throughout the country. However, service quality and availability of supplies varies across regions, depending in part on donor support— particularly WFP for the provision of MAM supplies and UNICEF for the provision of SAM supplies.

3.2.1.2 Screening platforms

Current policy calls for health districts to provide for **acute malnutrition screening at the community level (door-to-door) on a quarterly basis**, but resource constraints prevent full policy implementation/compliance in many districts. Partners often work with districts and pool resources from various sources so that the combined resources can cover the screenings. Even so, some districts are able to afford only two screenings per year, and sometimes integrate them with bi-annual CHDs. Despite the door-to-door approach, malnutrition screening coverage and quality during CHDs is generally low due to the heavy workload placed on health workers and Community Health Workers (CHW) to provide multiple interventions during these events. The availability of funding is an additional limiting factor for quarterly screening.

Child Health Days (CHDs) are centrally organized, donor-funded (mainly by UNICEF), door-to-door events, held twice yearly. They include vaccination, vitamin A supplementation (VAS), de-worming, and acute malnutrition screening for children under 5 years of age, and are sometimes integrated with National Immunization Days against polio. However, in practice, de-worming during CHDs is for children 24 -59 months, due to lack of de-worming medicine for children under 2 years and acute malnutrition screening is not realized everywhere.

Integrated Management of Childhood Illness (IMCI) is a national policy in Burkina Faso and CHWs are responsible for assessing children, treating common illnesses, and referring children with danger signs. Not all communities are covered by IMCI-trained CHWs. CHWs have some commodities available for sale, such as Oral Rehydration Salts (ORS), zinc, and artemisinin-combination therapy. Screening for acute malnutrition is included in the IMCI assessment. Cases identified during IMCI assessments are referred to health facilities.
National policy calls **for monthly well baby visits (CNS-Consultation du Nourrisson Sain)** for vaccination and growth monitoring at health facilities from birth through 2 years of age. In practice, children rarely attend beyond the age of one year (around the time of the measles/yellow fever immunization).

3.2.1.3 Referral and treatment schedules for MAM and SAM

The results of an acute malnutrition screening exercise using MUAC tapes can result in 3 types of diagnosis: i) negative (green code), ii) positive MAM (115mm≤MUAC<125mm; yellow code) or iii) positive SAM (MUAC<115mm or bilateral pitting edema; red code). In the case of SAM, mother-child pairs are

referred to the HC for a confirmatory diagnosis based on MUAC or WHZ criterion (WHZ < -3; WHO 2006 standard) and initiation of treatment. The mother will receive a referral slip (red color) with the MUAC value or edema observation. The community worker will not write down the name of the person but annotates the number of cases referred monthly. For MAM cases, the referral procedure is identical to the SAM procedure. Mother-child pairs are referred to the HC for confirmatory diagnosis and initiation of treatment depends on the type of village.
In case of a confirmatory diagnosis of SAM, the child will be examined further for medical complications. In the absence of such signs and after a successful appetite test, the child is included in an outpatient program with a weekly dose of RUTF (Plumpy Nut®, Nutriset, France). The mother will receive a referral slip with the HC's diagnosis destined for the CHW that ensures a follow-up role in the village. Children are discharged from this treatment schedule if they reach a WHZ≥-2 and a MUAC≥125mm and the absence of bilateral pitting edema for at least two consecutive weekly visits. Complicated cases of SAM or with clear signs of edema are referred for inpatient treatment to the district's hospital that disposes of an inpatient therapeutic feeding center (CREN).
In case of a confirmatory diagnosis of MAM by the HC staff, the child will be enrolled in a supplementary feeding program with Corn-Soy Blend fortified with micronutrients (CSB++) or supplementary Plumpy® (Nutriset, France).

3.2.1.4 Nutrition and health education

Currently in Gourcy, nutritional education group sessions (*causeries*) are organized at the HC level before or after CNS by CHV. These sessions are organized in larger groups and are focusing on delivering general messages about optimal breastfeeding practices, the preparation and introduction of complementary foods, growth monitoring, immunization and danger signs.

3.2.2 CMAM activities implemented by HKI for PROMIS in Gourcy District

3.2.2.1 Support to the national CMAM program

HKI supports the CMAM strategy as prescribed by National Nutrition Policy plan from Burkina Faso. For PROMIS, HKI general activities will include the continuation and expansion of the existing CMAM strategy as outlined above (*see paragraph 3.2.1*), focusing on the following tasks:

- **Capacity building and formative supervision to improve the quality of preventive and curative care at health center level** through nutrition capacity building and formative supervision (in CSPS and CREN for severe acute malnutrition with complications).

Capacity building will include formal trainings in CMAM and in data collection for newly appointed health staff if necessary, using district staff as trainers and following up training with regular supportive supervision by HKI. One of the lessons learned during previous CMAM programs is that formal trainings based on theoretical presentations are not sufficient to increase substantially the knowledge of participants or improve their practices. A successful alternative developed by HKI is the organization of Nutrition Days—two days of exchanges on nutrition for district staff. These capacity building sessions are hands-on and participatory, utilizing case studies, group work, counseling roleplays on specific nutrition topics identified by the staff during supervision, and other active learning methods. Nutrition Days will be organized for health professionals every 6 months to support continuous learning on effective approaches for preventing and treating malnutrition.
HKI supervisors and district health staff will conduct regular formative supervision visits to the 34 health centers and the CREN to improve the quality of nutrition care and data collection. The data collection tools are expected to be amended with the revision of the national protocol, and nurses will likely need additional support to comply with the new requirements. HKI supervisors and district nutrition focal points will coach health agents and provide immediate corrections and feedback until the CMAM services are well established in each health center, mentoring health staff on all components of the program (technical knowledge, counseling, data collection and reporting, and supply management).

- **Support to implementation of quality door-to-door screening for malnutrition each quarter by CHWs:** Coverage and utilization of services is expanded by intensifying structured, community-based strategies for active case finding and routine screening coupled with systematic door-to-door quarterly screenings in the targeted villages. A system of referral/counter-referral of the malnourished children is established through a partnership between the community, the health center and the in-patient nutrition rehabilitation center (CREN) for systematic follow-up of no-shows and drop-outs in the focus areas. This system will be strengthened with the coaching of HKI supervisors. Missed opportunities for early detection of acute malnutrition and ineffective methods for counseling caretakers will be reduced by enhanced supportive supervision for both health and community agents, including the modeling of appropriate techniques for “negotiating ‘doable’ behavior change”. This includes training for CHWs, financial support to the campaigns, and improved supervision by district health authorities. The quarterly screenings may be coupled with bi-annual VAS campaigns (JVA+/CHD) or stand-alone events.

- **Reinforcement of logistics management to strengthen the supply chain** in the district and region reducing the occurrence of stock-outs through an improved communication and collaboration with UNICEF and WFP.

3.2.2.2 Additional activities

- **Reinforcement of community-based CMAM activities:** Building of several previous successful experiences in other districts, HKI will encourage and support communities in the creation of Village Health/Nutrition Committees. Committee members will be selected by the community, and should include CHWs, grandmothers/older women leaders, and other village representatives applicable in the local context. The committee would assist in screening for malnutrition, home visits to follow-up drop-out cases, and social mobilization for BCC sessions, monitoring key health indicators within the community (mortality/cases of malnutrition) and displaying the information on a village “scoreboard”, promoting and facilitating cooking demonstrations of enriched porridge, etc. Each committee member would have a different role according to his/her status in the community. Grandmothers, for example, are more effective at influencing IYCF behaviors.

  In all areas, HKI will continue the strategy of training and regular, supportive supervision to strengthen capacities of community health volunteers (CHVs) for individual counseling and group discussions of ENA, early detection and referral of malnutrition cases, and collaboration with responsible health agents, expanding to cover the entire district. For prevention, HKI will apply its in-depth knowledge of cultural beliefs and practices in Burkina Faso as well as community mobilization tools and key-messages. Interpersonal counseling will be reinforced by radio and theatrical performances. The project will facilitate regular exchanges between CHVs and health agents as well as between CHVs and community leaders. The CHVs will be equipped to conduct active case finding and follow-up of referred cases.

- **Capacity building of traditional healers:** HKI will continue to expand the strategy of engaging traditional healers in support of CMAM. Traditional healers are often the first consultation sought by families with sick children, and if they are properly trained and supervised, they can expand coverage of screening and referral services and even preventive messages.

- **Distribution of SQ-LNS:**
  SQ-LNS will be distributed at the CSPS level when mothers bring their child for the monthly CNS. During CNS or during the BCC in small groups led by CHVs, caregivers of children 6-23 months old will be provided with a monthly dose (considering an average daily doses of 20g) and will be sensitize to the adequate use of SQ-LNS to situate the SQ-LNS in the overall child’s diet.
  During the CNS participating children will be screened for acute malnutrition and enrolled for treatment if needed. Such integration between BCC and screening is expected to have a synergetic impact on the reduction of acute malnutrition prevalence by preventing new cases of MAM/SAM and by

ensuring better coverage, referral and treatment rates of MAM/SAM cases. Indeed, also MAM/SAM cases will continue to participate in these group-counseling sessions allowing CHV to follow-up on their treatment schedule.

- **improved BCC and individual counseling for all children 0-23 months of age during CNS at health centers:**
  Improved BCC and individual counselling will be conducted by health workers at health centers during monthly CNS, which is the most frequent MAM/SAM screening platform for 0-23 month old children. ENA, IYCF, WASH and other health messages adapted to the age of the children will be provided individually or to small groups of expectant mothers or mothers of children of close age. Active, participatory training on interpersonal skills and counseling techniques will be provided to health workers. BCC for use of LNS will also be carried out during CNS at health centers in a timely manner for caregivers of 6-23 months old children.

3.3 Programme Impact Evaluation

The evaluation of the program model in the Gourcy district will compare two groups:

1) control: current national CMAM protocol supported by HKI, with BCC related to ENA/IYCF/WASH in typical larger groups (as currently implemented) for children between 0-23 months of age and reinforcement of community-based CMAM activities by HKI.
2) intervention: same as control + integration to monthly MAM/SAM screening at the health facility of a) improved BCC related to ENA/IYCF/WASH in small groups for children between 0-17 months of age and b) SQ-LNS distribution with appropriate BCC to children 6-17 months of age

3.3.1 Study design

The study design entails a two-arm stratified cluster-randomized, non-masked, community-based, effectiveness trial. The unit of randomization will be the catchment area of a health center (CSPS).
Both arms will benefit from a reinforced CMAM program so that it fits the national CMAM protocol with enhanced community-based management of acute malnutrition. Children in the intervention arm will receive additional monthly SQ-LNS and LNS-use sensitization combined with monthly individual or small-group BCC counseling sessions during monthly MAM/SAM screening at the health facility.

Unlike the targeted child age range of the program, being 0-23 months, we opt to limit the evaluation to the first 18 months of life. This choice has been made out of practical reasons being a limited budget available for evaluation and constrained

calendar. However, it should be noted that most of child acute malnutrition in Sub-Saharan Africa is incident during the first 12 months of a child's life [12].

Because of the intended dual role of BCC/SQ-LNS on child undernutrition in this study – e.g. to help prevent child undernutrition and enhance the coverage of screening, referral and treatment of SAM/MAM, it is necessary to combine two study designs to rigorously evaluate the impact of the proposed intervention and to tease out the contribution of prevention and enhanced coverage/treatment to the overall impact on child malnutrition.

The proposed study will therefore use two types of study designs. The first one is a **repeated cross-sectional design** that will compare select study outcomes between intervention and control groups at endline, after 24 months of program implementation. A repeated cross-sectional study design among children 0-17 months, at baseline and after 18 months (on different children) will be used to assess the impact of the intervention on the *prevalence* of several outcomes, including the prevalence of MAM/SAM and stunting, the coverage of MAM/SAM screening and maternal ENA/IYCF/WASH knowledge and practices. The second proposed study design entails a **cohort design** whereby individual children will be recruited at birth and followed-up monthly until they reach 18 months of age. We anticipate needing approximately 5 months to recruit the required number of children (estimated at 2,040). This design will allow us to assess the intervention's effects on the *incidence*, recovery and recurrence rates of MAM/SAM.

3.3.2 Randomization

The study area encompasses 34 catchment areas of health centers in the Gourcy health district situated in the North region in Burkina Faso. Random allocation of these 34 clusters to intervention and control arms will be conducted during a community ceremony in the presence of local health authorities. Small identical papers with the cluster names will be mixed in a bag and a volunteer will draw one paper at a time. The allocation will alternate between both study groups. The first draw will be allocated to the control group, the next to the intervention group. This pattern will be followed until catchment areas have been exhausted.

3.3.3 Repeated cross-sectional study

- *Study outcomes*

The first primary study outcome is the prevalence of child acute malnutrition (MAM/SAM) at endline. An anticipated lower MAM/SAM prevalence after intervention could be the result of either a successful prevention of MAM (less new

cases) or a better case-finding, referral and treatment of MAM and SAM or a combination of both.
The second primary outcome is the coverage of screening and treatment for child acute malnutrition (MAM/SAM). This operational outcome is crucial in evaluating the new program dynamic by combining child acute malnutrition screening with a preventive component using the delivery platform of monthly well baby visits (CNS). Allowing at least one month of program exposure, we evaluate the intervention effect on a sample of children aged 1-17 months instead of 0-17 months.

Primary study outcomes include:

- Prevalence of MAM defined by WHZ<-2 or MUAC[1]<125mm or bilateral pitting edema at study endline in children between 1 and 17 months of age
- MAM and SAM screening coverage (proportion of children monthly screened / total number of eligible children) for children between 1-17 months of age.
- MAM and SAM treatment compliance for (number of MAM and SAM children adhering to weekly treatment until discharged / number of MAM and SAM children starting treatment)

Secondary study outcomes include:

- Prevalence of child stunting defined by HAZ<-2 for children between 1 and 17 months of age.
- Mean WHZ-score at endline for children between 1 and 17 months of age;
- Mean HAZ-score at endline for children between 1 and 17 months of age;
- Mean MUAC at endline in children between 0 and 17 months of age;
- Mean hemoglobin concentration at endline in children between 3 and 17 months of age;
- Prevalence of child anemia (Hb concentration<10g/dL) at endline in children between 3 and 17 months of age;
- Prevalence of SAM defined by a WHZ<-3 or bilateral pitting edema or a MUAC<115mm
- Prevalence of severe stunting defined by a HAZ<-3
- Caregiver’s knowledge of indicators of IYCF, ENA and WASH
- Caregiver’s practices related to IYCF, ENA and WASH

- *Study size*

[1] Throughout the study, MUAC will only be used among children aged ≥ 6 mo

In order to detect a 30% decrease in MAM/SAM prevalence (assuming 15.7% MAM/SAM prevalence for children 1-17 months of age at baseline) with a statistical power of 90%, assuming a type I error of 5%, a coefficient of inter-cluster variation k of 0.3 and a non-response rate of 15%, a total number of ≈34 clusters with an average cluster size of 70 children is required, which results in a total sample size of 2,380 children – 1,190 for the control arm (17 clusters) and 1,190 for the intervention arm (17 clusters) [13].

- *Sampling strategy and study enrollment*

Between October 2014 and January 2015 a first cross-sectional study will be organized before the program is implemented to document the pre-intervention situation. An identical endline cross-sectional study will be conducted during the same months in 2016-2017 to counter any monthly seasonal variations. From every health center catchment area 2 villages will be selected with a probability proportionate to their population size in terms of children 1-17 months of age. Thereafter, 35 households with at least one index child fulfilling the inclusion criteria will be randomly selected from a census list for the selected village, to obtain the minimally required 70 children that are necessary per health center catchment area.

Inclusion criteria for the cross-sectional surveys are:
- At least one index child 1-17 months of age in the household
- Mother should be living in the study area since the index child's delivery
- Singleton infants

Exclusion criteria for the cross-sectional surveys are:
- Index child should not present congenital deformations that hamper anthropometric measurements

Age is an important covariate for anthropometry and IYCFI, therefore we opt to stratify the sampling by age to ensure comparable samples from the control group and the intervention group as given in table 2.

Table 2 Stratification by age for control and intervention clusters

Stratification by age	17 control clusters	17 intervention clusters
1-5 months	397	397
6-11 months	397	397
12-17 months	397	397

3.3.4 Longitudinal study

- *Study outcomes*

Primary study outcomes include:

- Incidence of MAM/SAM defined by WHZ<-2 or MUAC<125mm in children followed-up monthly from 0 to 18 months of age
- Coverage of child screening for acute malnutrition (% of eligible children screened over the total follow-up period)
- Compliance to treatment of acute malnutrition (% of cases that complete treatment of MAM and SAM over the total follow-up period)

Secondary study outcomes include:

- Incidence of child stunting defined by HAZ<-2 in children followed-up monthly from 0 to 17 months of age
- Relapse rate after treatment of MAM/SAM (%WHZ<-2 or MUAC<125mm or bilateral pitting edema after discharge from MAM or SAM treatment program)
- Linear growth velocity (HAZ increment/month)
- Ponderal growth velocity (WHZ increment/month)
- Weight gain (weight increment/month)
- MUAC gain (MUAC increment/month)
- Infant morbidity (expressed as longitudinal prevalence): acute respiratory infections, fever, diarrhea, vomiting and malaria

- *Study Size*

To ensure maximal comparability between results of the repeated cross-sectional study and the longitudinal study we opt to work in the same 34 clusters of the Gourcy district. Randomizing 34 clusters with an average cluster size of 60, assuming a coefficient of inter-cluster coefficient of variation k of 0.3 and a dropout rate of 20% would allow to detect a 22 % difference in MAM/SAM incidence over a follow-up period of 18 months (from 0 to 17 months of age) with a statistical power of 90% and a type I error of 5% [13]. Hence the total sample size for the longitudinal study is 2,040 or 1,020 for the control group (17 clusters) and 1,020 for the intervention group (17 clusters).

- *Sampling strategy and study enrollment*

From December 2014 to April 2015 children 0-6 weeks of age and fulfilling the study's inclusion criteria will be enrolled in the cohort. For this purpose mass gatherings will be organized in every village of the study area. Community workers will be asked to gather all children of that age to be screened for study eligibility.

Inclusion criteria for children for the longitudinal study are:

- 0-6 weeks of age;
- Mother should be living in the study area since the index child's delivery
- Singleton infants

Exclusion criteria include:
- Congenital malformations that make anthropometric measurements impossible
- Mother planning to leave the study area in the coming year
- Children of 1 month of age or older at study inclusion
- WHZ<-2 both at enrollment and at the first follow-up.

3.3.5 Measurement and Procedures

3.3.5.1 Questionnaires

- Health center level: A module with questions will be administered to the health center's staff. Questions on past, current and future projects related to health and nutrition in the project area will be asked. Interviewers will conduct a facility assessment enlisting available protocols related to CMAM, but also any preventive strategies on child malnutrition.
  Health staff's IYCF/ENA and WASH knowledge will be assessed. Furthermore, available health statistics from the health information system will be annotated. Time allocation of staff to CMAM at health center and during outreach activities, overall job motivation and job satisfaction will be documented. Finally, perceived barriers and boosters on CMAM treatment will be recorded.

- CHV level: a model with questions on ENA/IYCF/WASH knowledge, time allocation, motivation, job supervision, and job satisfaction will be administered. Additionally, specific questions will focus on the screening, referral and follow-up of MAM/SAM cases under treatment in the outpatient CMAM part. Furthermore, questions on previous participation in BCC will be formulated.

- Household level: Questionnaires on household composition, socio-economic status, household expenditure, household food security, cattle/land/asset ownership and construction materials of the concession will be administered. In addition, there will be a module of questions on food hygiene, hygienic practices and sanitation. Furthermore, household participation in social or NGO or government-related programmes will be assessed.

- Caregiver and child level: Questions on father and mother's education level, primary profession, decision power, maternal post-partum stress and depression and the index child's health and nutrition will be administered.

Furthermore, specific questions on ENA/IYCFI/WASH knowledge and practices will be asked to the child's primary caregiver. Further topics include utilization of health care services and contact moments with CHV. Child's age will be obtained from a birth certificate or using a locally adapted event calendar in the case an objective source of date of birth is lacking.

3.3.5.2 Anthropometry

The child's weight will be recorded using an electronic scale (SECA 876, Germany) to the nearest 100g. Length will be recorded to the nearest 1 mm using a length board (SECA 417, Germany). Mid-upper arm circumference will be recorded by using non-stretchable tape with 0.1cm precision (SECA 201, Germany). All measurements will be taken in duplicate by an anthropometrist and an assistant. All measurements will be exercised before the study through standardization exercises. Standardization exercises recording measurements from 10 children between 0-23 months of age will be repeated bimonthly. From these standardization sessions inter- and intra-observer variation of measurement error will be documented. Maternal MUAC, weight and height will also be recorded using non-stretchable tape (SECA 201), scales (SECA 877) and stadiometers (SECA 217, Germany) respectively. WHZ and HAZ scores will be calculated using the 2006 WHO growth reference.

3.3.5.3 Morbidity and mortality

Data collectors from the evaluation team will conduct monthly home visits to recall infant morbidity (Acute Respiratory Infections, Diarrhea, Fever and Malaria) of the last 3 days. A diarrheal episode is defined by at least 3 liquid or semi-liquid stools in the last 24h. Fever will be recorded by a standard thermometer, but will also be recalled. The presence of acute respiratory infection (ARI) during the previous week will be assessed by recalling specific ARI-related symptoms (cough, difficult breathing, grunting, rapid breathing, nasal secretion). Malaria will be diagnosed with using a rapid diagnostic test by taking finger blood from the child with a lancet in case a body temperature of more than 37.5°C is measured or if the mother reports the occurrence of a child fever episode over the last 12 hours. Using a 3-day recall for morbidity often leads to only a small to moderate loss of statistical power compared with a 7-day recall period, especially if morbidity signs are common, if the number of measurements per individual exceeds 10 or 12 and in cluster randomized trials [14].
Morbidity outcomes will be expressed as a longitudinal prevalence by taking the proportion of the days with recalled symptoms over the observed period in days.

3.3.5.4 Hemoglobin in capillary blood

Hemoglobin concentration will be measured at baseline and endline of program. For this purpose finger blood will be sampled. Hemoglobin concentration will be measured by spectrophotometry using a HemoCue device (HemoCue Ltd, Dronfield, United Kingdom). The device will be calibrated on a daily basis using a HemoCue Control Cuvette.
Child anemia and severe anemia is defined by a hemoglobin concentration less than 11g.dL^{-1} and less than 7 g.dL^{-1} respectively.

3.3.5.5 Cost and cost-effectiveness

Costs will be estimated from the societal perspective, thereby including all relevant costs incurred by institutions and communities [15]. Costs will be calculated with a combination of accounting records and "ingredients" estimates using unit costs and quantities of inputs [16]. An activity-based costing (ABC) methodology will be employed, allowing categorization and allocation of all costs of the program to its primary activities. Financial costs as well as economic costs (in-kind donations, household costs, etc.) will be collected. To obtain this data, program documentation and expenditure information will be reviewed, and key informant interviews and focus group discussions (FGDs) will be conducted with relevant program implementation staff. Another output of these interviews and FGDs will be time allocation estimates; staff time allocation will be used according to ABC methodology to allocate personnel costs among activities. FGDs will be conducted with households to gain an understanding of the direct and indirect costs they incur while participating in the program. The effect of plausible variation in household cost estimates from FGDs will be assessed during sensitivity analysis.
Analysis will focus on both total program costs, and incremental costs of the additional activities in the intervention area. Cost Effectiveness ratios (CER) will be calculated using costs and outcomes of the program. Incremental cost-effectiveness ratios (ICER) will be calculated by dividing the additional costs in the intervention area by the number of cases of acute malnutrition averted in the intervention area compared to the control area. This ratio represents the additional cost to achieve an additional successful outcome in the intervention area relative to the control area. Comparison with other similar published works will be considered where appropriate.

All aforementioned measurements except for the cost-data will be part of the cross-sectional surveys. For the longitudinal study, monthly anthropometry, child morbidity, questions on age-specific IYCF/ENA/WASH practices will be included as well as a monthly recall of participation in either mass screening campaigns during SIAN, routine screening by HC, CHW or CHV or screening during monthly PROMIS counseling sessions. A separate module of questions related to referral and treatment will be foreseen in case a child was found acutely malnourished.

3.3.6 Data management and analysis

Enumerators will use small laptops with Computer Assisted Personal Interviews (CAPI) for this survey work (SurveyBe software).

Baseline data from the cross-sectional survey will be used to assess the quality of the cluster randomization and document the pre-intervention situation. For this purpose, cluster and individual level means and standard deviations will be presented if variables are normally distributed. Medians and interquartile distances will be given in case continuous variables do not follow a normal distribution. Proportions will be presented for categorical variables.

The intervention effect on outcomes from the repeated cross-sectional study will be analyzed using the double difference specification that calculates the difference between endline outcomes in the control and intervention arm and subtracts the difference between baseline outcomes in those same arms to construct an estimate of program impact. Although the randomization is expected to minimize average differences at baseline between groups, this approach accounts for any remaining small differences at baseline, improving the precision of estimates. Formally, estimating double-difference impacts translates to running the Ordinary Least Squares (OLS) regression shown in equation 1.

$$Y = \beta_0 + \beta_1 * \text{Intervention} + \beta_2 * \text{Time} + \beta_3 * \text{Intervention} * \text{Time} + \varepsilon \quad \text{(eq. 1)}$$

With ***Intervention*** being a binary variable designating either control or intervention group, ***Time*** being a binary variable that either designates the baseline or endline survey time point. The double difference is then given by the $\boldsymbol{\beta_3}$ coefficient.
These regression models will further be adjusted for clustering at health center and village level. Furthermore, the analysis will be adjusted for covariates that differ more than 5% at baseline. Linear and binary logistic regression models will be used for continuous and binary outcomes respectively.

Ponderal (weight, MUAC and WHZ increments) and linear growth (length and HAZ increments) obtained from the longitudinal study, will be modeled using mixed-effects model with restricted splines with knots at 6, 12 and 18 months of child age. Covariates include the intervention group allocation, linear and spline terms for time (in months), and interaction terms between intervention group allocation and time. Models will further be adjusted for sex, primiparity, the baseline value of the outcome, stratum of health centers and interaction terms between these variables and time. Interactions between intervention allocation with baseline covariates will be inspected. The intervention effect will be statistically tested using a likelihood ratio test comparing a model with and without the interaction terms between intervention allocation and linear and nonlinear terms for time.

For the analysis of incidences (wasting) we will use mixed-effects Poisson regression models, while binary outcomes (eg. longitudinal prevalence of disease, stunting) will be analyzed using mixed-effects logistic regression models.

The applied mixed-effects models will use random effects for health center catchment area (level-3), village (level-2), individual (level-1) to take into account the effect of clustering at these levels and to estimate the standard error in an unbiased manner.

In order to assess the robustness of the findings, we will impute for missing data for outcomes using a multiple imputation strategy with chained equations. Data will be analyzed on intention-to-treat basis.

Data management, data cleaning and statistical analyses will be conducted using Stata 13.1 (Statacorp, USA). The statistical significance for all tests will be set at 5% in case of testing main effects or 10% in case of interactions. All statistical tests will be two-sided.

4 Ethical approval

The study protocol will be submitted for revision to the IFPRI IRB (Washington DC, USA) and to the National Ethics Committee of Burkina Faso (Ouagadougou, Burkina Faso). The intervention study will be registered at clinicaltrials.gov.

Care will be taken to provide the best available treatment to address children needs. In case the evaluator team would encounter children that suffer from acute malnutrition or from severe anemia (Hb<7 $g.dL^{-1}$), the mother-child pair will be referred with a letter of referral.

Following principles will be adopted:

- Upon IRB approval of the protocol HKI Burkina Faso will inform local and regional health authorities and community representatives. In every village of the study area HKI representatives will present the study's objectives and procedures to village authorities (village leaders, religious leaders) and demand their authorization.
- Voluntary and informed participation: The study's objective and procedures will be explained to all caregivers and head of household of eligible children in their local language. All informants reserve the right to refuse to participate in the study. Each enumerator will be asked to read the consent statement in full form, slowly and in local language to the participant. They will then ask if the consent statement was understood and if there are any questions. Then the enumerator will ask the mother/caretaker if they would like to participate in the baseline survey questions. At this time the informant will be given the opportunity to refuse and understand that they may also be able to refuse the study at any time during the survey with no repercussions to them or their family. Consent for children will be provided by their mothers or legal guardians. An informed consent form will be signed by the primary caregiver of the child and, if present, the head of household.

- Risks and serious adverse events: there are no known risks related to the evaluation methods (questionnaires, anthropometry). Good clinical practices will be respected by drawing capillary blood to dose hemoglobin concentration.
- Emergencies: All study subjects of both control and intervention arms will benefit from medical care in case a medical emergency is encountered during the evaluation visits. Medical emergencies include cases of severe anemia or any danger sign that would make competent medical staff conclude that hospitalization is required (eg. Non-response to induced pain, persisted vomiting, unconsciousness, etc.). The program will carry any medical costs related to such interventions (consultation, prescriptions, hospitalization if decided by competent medical staff) until discharge. Chronic diseases are not covered by this engagement. The management of acute malnutrition is available in every health center free of charge.
- Anonymity: Care will also be taken to ensure anonymity of participants during data collection, data management, and data analysis and result dissemination. Data forms will be made anonymous by using identification codes.

5 Partnership and management

This study is a collaboration between HKI and IFPRI. HKI Burkina Faso country team under supervision of the HKI regional office will be the implementer of the PROMIS program. IFPRI is in charge of the evaluation of the study. Both organizations guarantee the adherence to the study protocol. Figure 3 enlists the responsibilities of all actors of the project. In table 3 an overview of all involved parties is provided.

Table 3 Overview of project's actors

Project stakeholder	Name	Role
Sponsor and Scientific Partner	**IFPRI**<br>2033 K St, NW<br>Washington, DC 20006-1002<br>USA<br>Phone: +1 202-862-5600<br>Fax: +1 202-467-4439<br>Email:<br>- Dr Marie Ruel (PI)<br>M.Ruel@cgiar.org<br>- Dr Lieven Huybregts<br>L.Huybregts@cgiar.org<br><br>**IFPRI Dakar**<br>Titre 3396, Lot #2<br>BP 24063 Dakar Almadies<br>Senegal<br>Phone: +221.33.869.9800	- Principal investigator<br>- Design of the of study<br>- Quality control of the data collection<br>- Dissemination of the results<br>- Guarantor of research protocol

	Email:<br>- Dr Rahul Rawat (PI)<br>R.Rawat@cgiar.org<br>- Dr Elodie Becquey<br>E.Becquey@cgiar.org<br>- Dr Agnes Le Port<br>A.Leport@cgiar.org	
**Implementing Agency**	**HKI West Africa**<br>28, Nord Liberté 6. VDN X<br>Route du Front de Terre à<br>Dakar. N° 1A. BP. 16<br>511 Dakar Fann, Senegal<br>Telephone: + 00 221 33 869<br>55 01 Fax: + 00 221 33 827<br>24 06<br>- Dr Jennifer Nielsen<br>jnielsen@hki.org<br>- Raphael Bajay Tchumah<br>(Program manager)<br>RTchumah@hki.org<br><br>**HKI Burkina Faso**<br>Zone du Bois, Rue Ganga,<br>Porte 330<br>06 BP 9515<br>Ouagadougou 06<br>Burkina Faso<br>Telephone: +226 50 36 00 23<br>- Dr Jean-Céleste Somda<br>Jsomda@hki.org<br>- Fanny Yago-Wienne<br>fyago-wienne@hki.org<br>- Laura Barett<br>lbarett@hki.org<br>- Dr Regina Khassanova<br>rkhassanova@hki.org	- Coordination of implementation of program<br>- Project management<br>- Training and formative supervision health staff and CHV<br>- Financial management<br>- Ensure logistics of SQ-LNS

6 Budget

The PROMIS project is funded by the Department of Foreign Affairs, Trade and Development from Canada. Table 4 gives an overview of he main budget posts.

Table 4 Overview Project budget

Type	Cost (US$)

Materials	352,517.00
Service Contracts	561,183.00
Reimbursement medical costs	30,000.00
Incidental (10%)	94,370.00
Overhead (20%)	207,614.00
**Total**	**1,245,684.00**

7 Timeline

In Table 5 an overview of the different components of the PROMIS program is given. The implementation of the study will be finalized at the end of 2016, where after data analysis and report writing will be conducted. Project results will be disseminated through scientific publications and technical reports that will be published on the partners' websites.

Table 5 Projected timeline of the activities of the PROMIS project

Activities	2014		2015				2016				2017			
	Q3	Q4	Q1	Q2	Q3	Q4	Q1	Q2	Q3	Q4	Q1	Q2	Q3	Q4
Obtaining Ethics approval	X													
Training enumerators	X	X												
Baseline survey		X												
Refresher training health staff and CHV		X												
Longitudinal survey		X	X	X	X	X	X	X	X	X	X			
Endline survey										X	X			
Data analysis											X	X		
Dissemination of results												X	X	

8 Reference list

1. Black RE, Victora CG, Walker SP, et al. Maternal and child undernutrition and overweight in low-income and middle-income countries. *Lancet*. 2013;382(9890):427-451.

2. WHO. *Technical Note: Supplementary Foods for the Management of Moderate Acute Malnutrition in Infants and Children 6–59 Months of Age*.; 2012:1-12. doi:10.1227/00006123-199112000-00028.

3. Nyirandutiye DH, Ag Iknane A, Fofana A, Brown KH. Screening for acute childhood malnutrition during the National Nutrition Week in mali increases treatment referrals. *PLoS One*. 2011;6(6):e14818. doi:10.1371/journal.pone.0014818.

4. De-Regil LM, Suchdev PS, Vist GE, Walleser S, Peña-Rosas JP. Home fortification of foods with multiple micronutrient powders for health and nutrition in children under two years of age (Review). *Evid Based Child Health*. 2013;8(1):112-201. doi:10.1002/ebch.1895.

5. Bhutta ZA, Das JK, Rizvi A, et al. Evidence-based interventions for improvement of maternal and child nutrition: What can be done and at what cost? *Lancet*. 2013;382(9890):452-477.

6. Bhutta Z, Rivzi A, Raza F, et al. A comparative evaluation of multiple micrnonutrient and iron-folic acid supplementation during pregnancy in Pakistan: impact on pregnancy outcomes. *Food Nutr Bull*. 2009;(30):S496-S505.

7. Huybregts L, Houngbé F, Salpéteur C, et al. The effect of adding ready-to-use supplementary food to a general food distribution on child nutritional status and morbidity: a cluster-randomized controlled trial. *PLoS Med*. 2012;9(9):e1001313. doi:10.1371/journal.pmed.1001313.

8. Iannotti LL, Jean S, Dulience L, et al. Linear growth increased in young children in an urban slum of Haiti : a randomized controlled trial of a lipid-based nutrient supplement 1 – 3. 2014:198-208. doi:10.3945/ajcn.113.063883.198.

9. Dewey KG, Yang Z, Boy E. Systematic review and meta-analysis of home fortification of complementary foods. *Matern Child Nutr*. 2009;5:283-321. doi:10.1111/j.1740-8709.2009.00190.x.

10. Lewin S, Munabi-Babigumira S, Glenton C, et al. Lay health workers in primary and community health care for maternal and child health and the management of infectious diseases: a review synopsis. *Cochrane Database Syst Rev*. 2010;28(3):243-5. doi:10.1111/j.1525-1446.2011.00950.x.

11. Macro ORC. Enquête Démographique et de Santé et à Indicateurs Multiples (EDSBF-MICS IV). 2010.

12. Victora CG, de Onis M, Hallal PC, Blössner M, Shrimpton R. Worldwide timing of growth faltering: revisiting implications for interventions. *Pediatrics*. 2010;125(3):e473-80. doi:10.1542/peds.2009-1519.

13. Hayes RJ, Bennett S. Simple sample size calculation for cluster-randomized trials. 1999:319-326.

14. Schmidt W-P, Genser B, Barreto ML, et al. Sampling strategies to measure the prevalence of common recurrent infections in longitudinal studies. *Emerg Themes Epidemiol*. 2010;7(1):5. doi:10.1186/1742-7622-7-5.

15. Weinstein MC, Siegel JE, Gold MR, Kamlet MS, Russell LB. Recommendations of the Panel on Cost-effectiveness... [JAMA. 1996] - PubMed result. *JAMA*. 1996;276(15):1253-8. Available at: http://www.ncbi.nlm.nih.gov/pubmed/8849754.

16. Tan-Torres Edejer T, Baltussen R, Adam T, Hutubessy R, Acharya A, Evans DB and Murray C. WHO GUIDE TO COST-EFFECTIVENESS ANALYSIS. In: *Global Programme on Evidence for Health Policy ,World Health Organization, Geneva*.; 2003.
