# Supplementary material for: Impact on child acute malnutrition of integrating a preventive nutrition package into facility-based screening for acute malnutrition during well-baby consultation: A cluster-randomized controlled trial in Burkina Faso
Source: PLoS Med. 2019 Aug 27;16(8):e1002877. doi: 10.1371/journal.pmed.1002877 (PMC6711504; doi:10.1371/journal.pmed.1002877)
Supplement: S2 Text — PROMIS, Innovative Approaches for the Prevention of Childhood Undernutrition. (DOCX) [file pmed.1002877.s018.docx]

**Data analysis plan for:**

PROMIS Burkina Faso- Impact of integrating small-quantity lipid-based nutrient supplements into screening for child acute malnutrition at the well-baby consultation in health center: A cluster randomized controlled trial in Burkina Faso

Amended: June 2019

## Primary study outcomes

### *Repeated cross-sectional study*

Primary study outcomes for the cross-sectional study design are:

1. the prevalence of AM defined by a WLZ<-2 or a MUAC< 125mm (only in children older than 6 months) or the presence of bilateral pitting edema;
2. AM screening coverage defined as the number of children screened in the month preceding the survey (as reported by the caregiver) over the total number of eligible study children;
3. AM treatment compliance defined as the number of AM children under appropriate treatment at the time of the survey over the total number of AM cases identified in the study sample.

### *Longitudinal study*

For the longitudinal study, primary outcomes are:

1. Incidence of AM (same definition of AM as above);
2. monthly AM screening coverage (the number of children screened each month over the total number of eligible study children);
3. AM treatment compliance (the number of AM children adhering to weekly or bi-weekly treatment until discharged over the total number of AM children that were scheduled for treatment).

## Secondary study outcomes

Secondary study outcomes for the cross-sectional study are:

- Mean HAZ-score at endline (using WHO 2006 growth reference);

- Mean height at endline;

- Mean WHZ-score at endline (using WHO 2006 growth reference);

- Mean MUAC at endline;

- Mean hemoglobin concentration at endline;

- Prevalence of child anemia (Hb concentration<11g.dL-1) at endline;

- Prevalence of child severe anemia (Hb concentration<7g.dL-1) at endline;

- Prevalence of child stunting defined by HAZ<-2 (using WHO 2006 growth reference);

- Prevalence of MAM defined by a -3≤WHZ<-2 (using WHO 2006 growth reference) or a 115mm≤MUAC<125mm (only in children older than 6 months);

- Prevalence of SAM defined by a WHZ<-3 (using WHO 2006 growth reference) or bilateral pitting edema or a MUAC<115mm (only in children older than 6 months);

- Prevalence of severe stunting defined by a HAZ<-3 (using WHO 2006 growth reference);

- Caregiver’s knowledge of indicators of WHO recommended IYCF, CMAM and WASH;

- Caregiver’s practices related to IYCF, ENA and WASH;

- Immunization coverage (defined as the recommended immunization contacts by child age at which these vaccines should be received, following the national guidelines in Burkina Faso allowing for a tolerance 4 weeks after the recommended age point).
- Weight-for-age Z-score (added on June 20, 2019)
- Prevalence of underweight (added on June 20, 2019)

Secondary study outcomes for the longitudinal study are:

- Incidence of child stunting defined by HAZ<-2 (using WHO 2006 growth reference) in children followed-up monthly from 0 to 18 months of age;
- Longitudinal prevalence of AM, MAM and SAM defined by time the child was AM, MAM, SAM over the total follow-up time respectively;
- Change in mean AM prevalence over time;
- Treatment compliance of MAM and SAM (the number of MAM or SAM children adhering to weekly or bi-weekly treatment until discharged over the total number of MAM or SAM children that were scheduled for treatment);
- Enrollment into CMAM of AM, MAM or SAM children (caregiver report);
- Recovery rates of AM, MAM or SAM (defined as the proportion of children who were free from AM for at least one monthly measurement over the total number of children suffering from AM, MAM, and SAM respectively);
- Relapse rate after successful treatment of AM (%WHZ<-2 or MUAC<125mm (only in children older than 6 months) or bilateral pitting edema after discharge from MAM or SAM treatment program);
- Mean episode length for AM (an episode of AM was defined as starting from the moment a child was found to be acutely malnourished at the monthly survey visit until the moment the child was free from AM for at least one monthly measurement);
- Mean episode length for MAM or SAM (a MAM or SAM episode was defined as starting from the moment a child was found to be MAM or SAM at the monthly survey visit until the moment the child was free from AM for at least one monthly measurement);
- Linear growth velocity (HAZ increment/month);
- Ponderal growth velocity (WHZ increment/month);
- Weight gain (weight increment/month);
  - MUAC gain (MUAC increment/month);
- Longitudinal prevalence of infant morbidity: acute respiratory infections, fever, diarrhea, vomiting and malaria;
- Change in IYCF practices and caregiver knowledge;
- Immunization coverage (defined as the recommended immunization contacts by child age at which these vaccines should be received, following the national guidelines in Burkina Faso allowing for a tolerance 4 weeks after the recommended age point).

## Sample size calculation for primary study outcomes

We used Hayes and Bennet’s formulas^[[1]](#footnote-1)^ to calculate the necessary sample sizes for the repeated cross-sectional and the longitudinal study.

### *Cross-sectional study*

### Assuming a coefficient of inter-cluster (i.e. between health center catchment areas) variation k of 0.25, a non-response rate of 15%, a type I error of 5% and a statistical power of 80%, we calculate that an average cluster (i.e. health center catchment area) size of 72 children, 32 clusters (i.e. an overall sample size of 2,304 children) is needed for each survey round to detect a decrease in the prevalence of AM of 5.4 percentage points assuming a baseline prevalence of 16%. This sample size allows detecting a difference in AM screening coverage of 7.5 percentage points and a difference in AM treatment coverage of 20 percentage points between study arms assuming baseline values of 25% for both outcomes.

### *Longitudinal study*

Assuming a coefficient of inter-cluster variation k of 0.2, a dropout rate of 20%, a type I error of 5% and a statistical power of 80%, we needed to recruit 66 children in each of the 32 clusters (i.e. an overall sample size of 2,112 children total) to detect a 23.5% reduction in the incidence of AM over 18 months of follow-up, assuming a baseline incidence of 0.52 case^[[2]](#footnote-2)^ per child-year. This sample size allows us to detect a difference in AM screening coverage of 5.9 percentage points and a difference in treatment coverage of 9.0 percentage points during the 18 months of follow-up assuming an incidence of 0.52 cases per child-year and baseline values of 25% for both outcomes.

# Randomization procedure

In Burkina Faso, simple (i.e. non-stratified) random allocation will be used. Randomization will take place at a community event in Gourcy with local health authorities. Thirty-two identical pieces of paper with either ‘control’ (n = 16) or ‘intervention’ (n = 16) written on them will be mixed in a bag for randomization of the 32 rural health centers. The allocation of the urban health center will be conducted separately by drawing a piece of paper from a bag containing 2 pieces of paper (1 ‘control’ and 1 ‘intervention’). The primary impact analysis in Burkina Faso will be limited to the 32 rural health center catchment areas since the services offered and the population served in the urban health center are not comparable to those at the rural health centers.

# Sampling

A census to identify infants will be organized prior to the start of the cross-sectional surveys and prior to the beginning of the longitudinal study. When the health center catchment area include more than 3 villages, we will randomly select 3 villages (using probability proportional to population size sampling) and then randomly select an equal number of households with an eligible child using Stata. Since age is an important predictor of AM and nutrition and health related practices, we will stratify children in three equal age groups (0–5 m; 6–11 m; 12–17 m) and will draw a random sample from each age group for the cross-sectional study.

## Statistical analysis

Data will be analyzed on intention-to-treat basis. To allow for an analysis “as randomized” in the presence of missing data, we will impute missing data of the longitudinal study using an appropriate multiple imputation strategy under the missing-at-random assumption. Data management, data cleaning and statistical analyses will be done using Stata 14 or 15 (Statacorp, USA). The statistical significance will be set at 5%. All statistical tests will be two-sided. The analysis of primary study outcomes will be adjusted for multiple testing (n=6 primary study outcomes).

Any imbalance in covariates at baseline will be assessed in absolute terms (e.g. is the difference in means or prevalence larger than 5%?). Baseline differences will not be subject to statistical testing.

### *Repeated cross-sectional study*

The repeated cross-sectional study design will be used to estimate program impact after 2 years of program implementation. Linear and linear probability mixed-effect regression models will be used for continuous and binary outcomes respectively. In case of linear probability models we will use a robust estimation of standard errors to account for heteroscedasticity of the residuals (added on June 20, 2019). Although the randomization is expected to minimize average differences at baseline between groups, we will adjust regression models for baseline values of the outcome of interest and covariates that should not have changed as a consequence of the intervention to improve the precision of the estimates. Regression models will further be adjusted for clustering at health center catchment area level. Exploratory analysis will assess effect modification by health center characteristics by testing interaction terms and if statistically significant at 10% level considered for sub-group analysis.

### *Longitudinal study*

Ponderal (weight, MUAC and WHZ increments), linear growth (length and HAZ increments), screening and treatment coverage obtained from the longitudinal study, will be modeled using mixed-effects model with restricted splines to account for a likely non-linear character. For binary outcomes we will use linear probability spline models; we will use a robust estimation of standard errors to account for heteroscedasticity of the residuals (added on June 20, 2019). Covariates include the intervention group allocation, spline terms for time (in months), and interaction terms between intervention group allocation and spline term time. Models will further be adjusted for sex, first live birth (yes/no), the baseline value of the outcome. The intervention effect will be statistically tested using a likelihood ratio test (or by an equivalent omnibus test) comparing a model with and without the interaction terms between intervention allocation and spline terms for time. Interactions between intervention allocation and baseline covariates will be inspected assess possible effect modification.

For the analysis of AM incidence and the longitudinal prevalence of morbidity, we will use mixed-effects Poisson regression models with robust estimation of standard errors. The mixed-effects models will be adjusted for clustering by health center catchment area and individual to estimate the correct standard errors. Models will be further adjusted for child age, first live born (yes/no), child sex.

1. Hayes RJ, Bennett S. (1999) Simple sample size calculation for cluster-randomized trials. International Journal of Epidemiology; 319–26 [↑](#footnote-ref-1)
2. We estimated a baseline AM incidence of 0.52 cases per child-year assuming a 16% AM prevalence (~14% MAM prevalence) and an incidence conversion factor K of 3.2 (Isanaka et al. 2011) [↑](#footnote-ref-2)
