# Supplementary material for: Impact on child acute malnutrition of integrating a preventive nutrition package into facility-based screening for acute malnutrition during well-baby consultation: A cluster-randomized controlled trial in Burkina Faso
Source: PLoS Med. 2019 Aug 27;16(8):e1002877. doi: 10.1371/journal.pmed.1002877 (PMC6711504; doi:10.1371/journal.pmed.1002877)
Supplement: S3 Text — (DOCX) [file pmed.1002877.s019.docx]

**Impact sur la malnutrition aiguë infantile de l’intégration d’un paquet préventif au dépistage de la malnutrition aiguë au centre de santé pendant la consultation du nourrisson sain : un essai contrôlé randomisé par grappes au Burkina Faso**

**Résumé scientifique**

**Contexte**

La prise en charge de la malnutrition aiguë (PECMA) est une approche très efficace pour traiter les enfants souffrant de malnutrition aiguë (MA), dont le risque de mortalité serait sinon considérablement supérieur. Pourtant, en contexte programmatique, l’efficacité de la PECMA est limitée par une couverture faible du dépistage de la MA, en partie à cause d’un manque de bénéfice perçu par les responsables des enfants.

Au Burkina Faso, le dépistage de la MA est conduit mensuellement chez les enfants de moins de deux ans lors de la consultation du nourrisson sain au centre de santé. Nous avons émis l’hypothèse que l’intégration au dépistage mensuel d’un paquet préventif, incluant une communication pour le changement de comportement (CCC) appropriée par rapport à l’âge de l’enfant et portant sur les pratiques de nutrition, santé et hygiène, et l’approvisionnement pour un mois en supplément nutritionnel préventif à base lipidique (SQ-LNS) pour les enfants de 6 mois ou plus, augmenterait les couvertures du dépistage et du traitement de la MA, et diminuerait l’incidence et la prévalence de la MA.

**Méthodes et résultats**

Nous avons eu recours à un essai contrôlé randomisé par grappes, et avons alloué 16 centres de santé au groupe d’intervention, et 16 au groupe de comparaison. Les deux groupes avaient accès aux services standards de PECMA et de CNS ; dans le groupe d’intervention, les responsables des enfants recevaient également la CCC appropriée pour l’âge et le SQ-LNS pour les enfants de 6 mois ou plus.

Nous avons utilisé deux schémas d’étude : 1) une étude transversale répétée sur des enfants de 0-17 mois (n=2.318 et 2.317 lors de l’étude de base et de l’étude finale après deux ans) pour évaluer les impacts sur les couvertures du dépistage et du traitement et la prévalence de la MA ; 2) une étude longitudinale sur 2.113 enfants enrôlés peu après la naissance et suivis chaque mois pendant 18 mois, pour évaluer les impacts sur les couvertures du dépistage et du traitement et l’incidence de la MA. Les données ont été analysées en intention-de-traiter. Le niveau de signification statistique était égal à α=0.016 après ajustement pour tests statistiques multiples.

L’âge moyen des enfants était de 8.8 ± 4.9 mois dans le groupe d’intervention et 8.9 ± 5.0 mois dans le groupe de comparaison lors de l’étude de base ; et de respectivement 0.66 ± 0.32 et 0.67 ± 0.33 mois à l’enrôlement dans l’étude longitudinale. Par rapport au groupe de comparaison, le groupe d’intervention présentait une couverture du dépistage significativement plus élevée (*étude transversale* : +18 points de pourcentage (pp) ; IC95% : 10, 26 ; P<0.001; *étude longitudinale* : +23 pp ; IC95% : 17, 29 ; P<0.001). L’intervention n’a pas eu d’impact sur la couverture du traitement de la MA (*étude transversale* : +8.0 pp ; IC95% : 0.09, 16 ; P=0.047 ; *étude longitudinale* : +7.7 pp ; IC95% : -1.2, 17 ; P=0.090), l’incidence de la MA (*étude longitudinale* : IRR=0.98 ; IC95% : 0.75,1.3 ; P=0.88), ou la prévalence de la MA (*étude transversale* : -0.46 pp ; IC95% : -4.4, 3.5 ; P=0.82).

Une limite de l’étude est la référence des cas de MA (pour raisons éthiques) selon les mesures mensuelles prises par les enquêteurs de l’étude longitudinale, qui pourrait avoir atténué l’impact sur la couverture du traitement de la MA.

**Conclusions**

L’ajout d’un paquet préventif aux services de PECMA délivrés au centre de santé au Burkina Faso a augmenté la participation au dépistage mensuel de la MA, permettant ainsi de dépasser un obstacle majeur à l’efficacité de la PECMA. Le manque d’impact sur la couverture du traitement et sur la prévalence et l’incidence de la MA appelle à plus de recherche sur la réduction des autres barrières qui limitent encore l’adhésion aux services de prévention et de traitement délivrés dans les centres de santé ; et sur l’identification d’approches complémentaires pour amener des services intégrés de prévention et de PECMA plus près des communautés, tout en assurant une haute qualité de mise en œuvre et de livraison de service.

**Enregistrement de l’essai**

ClinicalTrials.gov NCT02245152
